# FAIMS-GPF XL-MS: crosslinking-mass spectrometry based on gas-phase fractionation

**DOI:** 10.1038/s41467-026-75736-9

**Published:** 2026-07-23

**Authors:** Hugo Gizardin-Fredon, Salvatore Terrosu, Simon Pichard, Zinedine Loukili, Dursun Korkut, Cathy Braun, Arnaud Poterszman, Clément Charenton, Sarah Cianférani

**Affiliations:** 1 Laboratoire de Spectrométrie de Masse BioOrganique, IPHC UMR 7178, Université de Strasbourg, CNRS, Strasbourg, France; 2 Infrastructure Nationale de Protéomique ProFI – UAR2048, Strasbourg, France; 3 Université de Strasbourg, Institut de Génétique et de Biologie Moléculaire et Cellulaire (IGBMC), Illkirch, France; 4 CNRS UMR 7104, Illkirch, France; 5 Inserm, U 1258, Illkirch, France; 6 Biostructure (CBI/IGBMC), CNRS UAR 2061, Inserm US 67, Université de Strasbourg, Illkirch, France; 7 Equipe Labellisée Ligue Contre le Cancer, Institut de Génétique et de Biologie Moléculaire et Cellulaire (IGBMC), Illkirch, France

**Keywords:** Mass spectrometry, Protein-protein interaction networks

## Abstract

Protein-protein interactions (PPIs) underpin nearly all cellular processes; therefore, mapping PPI and protein-protein association networks is critical for understanding how biological systems function in health and disease. However, many functionally relevant PPIs are transient and mediated by weak affinity interactions, making them challenging to detect. Using chemical cross-linking together with mass spectrometry (XL-MS) has emerged as an important category of methods for mapping PPIs, including those that are more dynamic and transient. However, XL-MS workflows face limitations which hinder their routine use, especially in proteomic platforms. Here, to address these limitations, we develop a XL-MS workflow based on gas-phase fractionation (GPF) using high-field asymmetric waveform ion mobility spectrometry (FAIMS). Our optimized FAIMS-GPF XL-MS protocols exhibit improved performance when handling both low-complexity samples, such as purified Cdk7-Activating Kinase (CAK) heterotrimer, and high-complexity cross-linked HeLa lysates. In HeLa lysate, we identify 1269 cross-links accounting for 213 PPIs without any in-solution fractionation, starting from less than 20 µg of sample. The method also proves effective for analyzing low-abundance, high-complexity samples such as spliceosomes. Here we show that FAIMS-GPF enhances the detection of cross-linked peptides and improves PPI coverage, thus offering a scalable, high-sensitivity solution for XL-MS integration with structural biology and functional proteomics applications.

## Abstract (web summary)

Brief summary for “FAIMS-GPF XL-MS: crosslinking-mass spectrometry based on gas-phase fractionation”. The need for large amounts of starting material remains a major challenge for large-scale cross-linking mass spectrometry (XL-MS) experiments. Here, the authors introduce FAIMS-GPF, a gas-phase fractionation strategy that eliminates the need for solution-based fractionation in XL-MS. This approach provides a sensitive, fast and adaptable workflow.

## Introduction

Protein-protein interactions (PPIs) are essential drivers of cellular processes and function, including signal transduction, gene expression, cellular metabolism, cell growth, proliferation, and apoptosis. In general, proteins rarely function in isolation; their ability to interact with other proteins to form specific complexes and PPI networks is essential for ensuring coordinated execution of cellular tasks^1–4^. Therefore, identifying and characterizing PPIs is of critical importance for understanding fundamental cellular processes and biological pathways, as well as how their dysregulation contributes to disease mechanisms and pathogenesis. Although the exact number of PPIs formed in any given human cell remains unknown, several recent efforts have yielded information on tens of thousands of PPIs^5^. For example, Human Reference Interactome (HuRI) includes approximately 53,000 high-quality binary PPIs^6^, and hu.MAP 2.0 contains information on more than 57,000 predicted PPIs encompassing close to 10,000 human proteins^7^. However, based on some estimates, even these resources cover only a small fraction of all the PPIs that govern cellular function^8^. Therefore, deciphering PPIs and their networks remains of high interest and importance.

Currently, the most powerful methods for mapping PPIs and the human interactome (*i.e*., the complete set of PPIs within a cell) are based on the use of mass spectrometry (MS). In this context, the application of affinity-purification (AP) MS^9^, whereby high-affinity baits are used to capture PPIs, has been especially powerful in identifying interactions between human proteins, most recently resulting in capturing more than 100,000 interactions among approximately 14,500 proteins^10^. However, AP-MS-based strategies underperform when detecting transient and low-affinity interactions, which are key determinants of cell signaling. On the other hand, although methods such as enzyme-catalyzed proximity labeling strategies (e.g. BioID, TurboID, APEX)^11–14^ coupled with MS are able to capture more transient interactions, they also have notable limitations, including the need to fuse proteins of interest with enzymes that generate reactive species needed to label the interaction partners. Additionally, the labeling radius in these experiments varies between 10–20 nm^9,15^, which may lead to off-target reactivity and false positives.

Cross-linking mass spectrometry (XL-MS) represents a distinct set of strategies for identifying PPIs and defining their interfaces, that uses chemical cross-linkers to form covalent linkages between interacting proteins before subjecting them to MS analysis^16^. The method is versatile and can be used with purified protein complexes^17^, organelles^18^, cells^4^, and even tissues^19^. XL-MS provides several advantages over the methods mentioned above, such as the ability to directly capture PPIs in their native environment without a priori, thus preserving physiologically relevant interactions. Additionally, XL-MS does not require the use of affinity reagents or fusion proteins and tags, and the availability of different cross-linkers with a wide range of chemical reactivity profiles makes these methods versatile and compatible with a wide range of samples^16^. However, the cross-linking reactions give low final yields, which leads to low intensity for cross-linked peptides in MS scans and limits PPI detection to high abundance proteins and stable interactions^20,21^. These effects amplify in the case of higher complexity samples such as organelles or whole cell lysates.

In general, the performance of XL-MS for PPI mapping and interface definition in complex samples can be improved by using different strategies to reduce sample complexity, such as the use of MS-cleavable cross-linking reagents, such as DSBU^22^ and DSSO^23^ or enrichable reagents, such as Protein Interaction Reporters (PIR)^24^, Azide-A-DSBSO^25^ or phosphonate-based PhoX^26^. The most extensive studies, although essential for uncovering a high number of PPIs, often rely on the use of off-line chromatographic fractionation^22,27–29^ with the collection of numerous fractions (> 20), which requires substantial amounts of starting material (>500 µg) and significantly increases instrument time. During past years, ion mobility techniques such as high-field asymmetric-waveform ion-mobility spectrometry (FAIMS) and trapped ion mobility spectrometry (TIMS) also emerged as a key strategy to improve peptides identification across various fields of proteomics. These techniques offer an additional separation dimension in gas-phase separation that is strongly influenced by peptides charges. In this later context, given that cross-linked peptides typically carry higher charge (≥3+) compared to linear peptides (+2), the use of FAIMS (High-field asymmetric-waveform ion-mobility spectrometry)^30,31^ in XL-MS workflows either with MS-cleavable reagents^30–32^ or enrichable PhoX^33^ resulted in significant improvement of cross-link identifications (XL-IDs) when combined with in-solution fractionation strategies. However, the use of FAIMS as a standalone gas-phase fractionation (GPF) method to fully replace in-solution fractionation has not yet been evaluated and may further enhance our ability to harness the power of XL-MS for identifying protein–protein interactions (PPIs) in complex systems.

Here, we develop a workflow that employs FAIMS-GPF, coupled to a fast and sensitive sample preparation using DSSO^23^ and automated peptide cleanup for improved XL-MS-based identification of PPIs. As a proof-of-concept, we apply this method for several case studies of different systems, a structural biology project focusing on Cdk7-Activating Kinase (CAK) trimer, a proteome-wide study of cross-linked HeLa lysate, and a study of PPIs in a challenging spliceosome sample. We demonstrate that the FAIMS-GPF XL-MS approach we describe achieves unprecedented sensitivity and throughput for all sample complexities. Furthermore, we show that eliminating in-solution fractionation enables the use of a consistent, adaptable and streamlined workflow across all sample types, providing smart protocols for performing routine XL-MS analysis.

## Results

### General description of the FAIMS-GPF XL-MS workflow

**Fig. 1:**
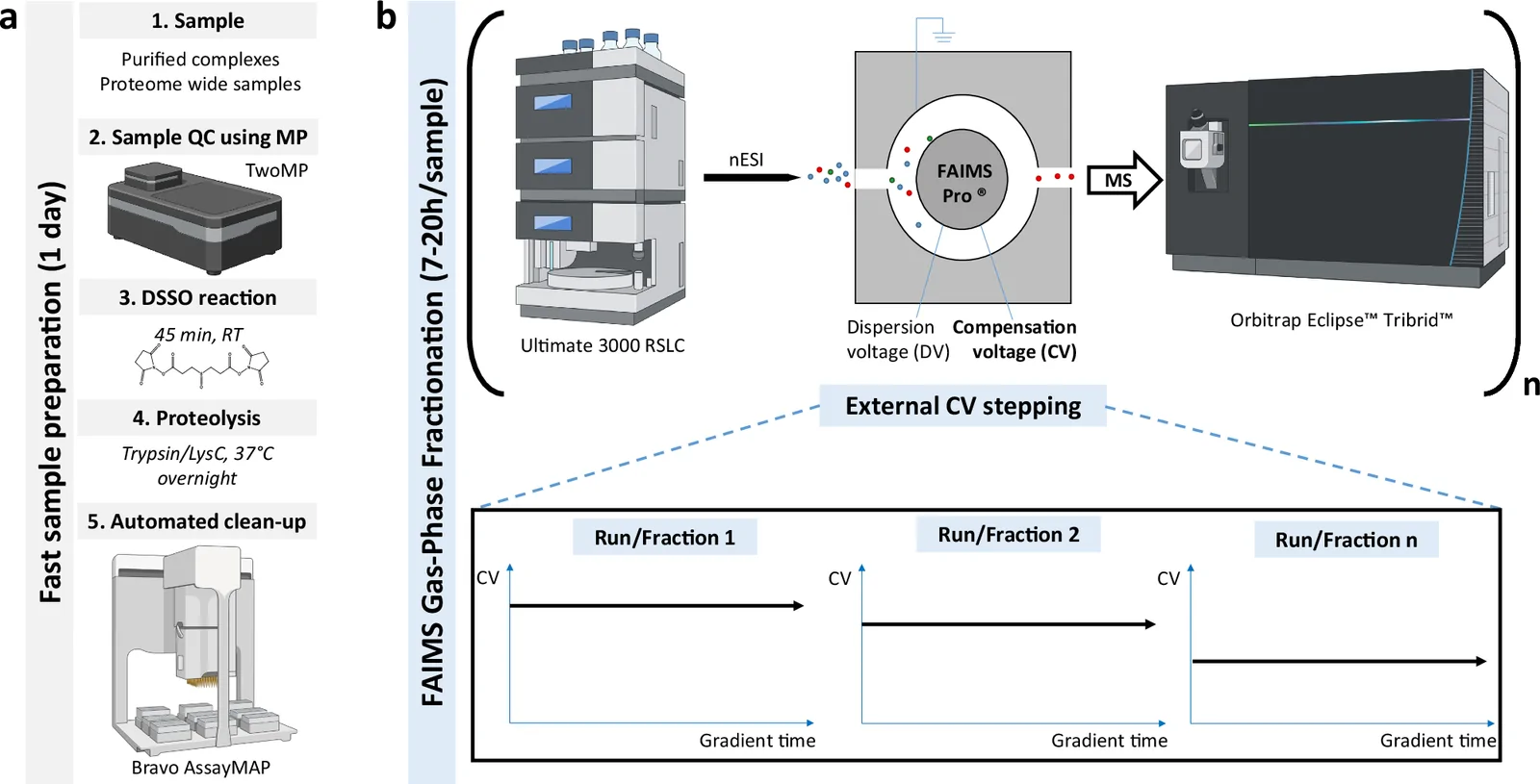
Experimental design for the evaluation of FAIMS-GPF in XL-MS workflows. **a** We applied a fast sample preparation step (1 day). After a mass photometry QC, samples are submitted to cross-linking reactions using DSSO, overnight digested with Trypsin/LysC and cleaned up using an automated solid-phase extraction (SPE) method on the Bravo AssayMAP robot. **b** For FAIMS-GPF, 400 ng are injected in successive LC-FAIMS-MS/MS runs, each time with a different compensation voltage (CV) in a so-called ‘external stepping’ fashion. We started by screening different CV values before selecting the best associations for our optimized methods on each sample. Created in BioRender. Hoernel, C. (2026) https://BioRender.com/h1juany.

To streamline and increase efficacy of the workflow, we optimized our sample preparation step to a 1-day process that uses mass photometry (MP) for quality control prior to a 45-min cross-linking reaction with DSSO, overnight Trypsin/LysC digestion and an automated solid-phase extraction (SPE) sample clean-up (Fig. 1a). The samples were then subjected to nano-scale liquid chromatography (nLC) prior to FAIMS filtering and tandem mass spectrometry (MS/MS) (Fig. 1b). Briefly, FAIMS uses an asymmetric waveform that alternates between a high-field voltage (called dispersion voltage, DV) of one polarity and a low field voltage of the opposite polarity^34,35^. If the ion has a different mobility between high and low field (due to charge, for example), it collides on one electrode and is not detected in MS. An additional direct current called “Compensation Voltage” (CV) allows compensating this ion displacement to bias the transmission of a subset of ions of interest^34,35^, which aims in our case at enriching cross-linked peptides in gas-phase^30,31,35^.

As the first step in developing our workflow, we evaluated two different FAIMS-based GPF modes using either (i) “internal CV stepping”, where a single LC gradient is followed by different successive compensation voltage (CV) values that alternate with a defined cycle time; or (ii) “external CV stepping” where analysis is done using several successive LC-FAIMS-MS/MS runs each with a single CV value^36^ (Supplementary Fig. 1a, b). We showed that external stepping is always superior to internal stepping, for both similar gradient length or similar total analysis time (Supplementary Figs. 1c, 2). We also showed that external CV stepping with a single tailored CV provides higher numbers of unique cross-links (XLs, corresponding to unique cross-link sites) than internal stepping, in agreement with published data^36,37^. Therefore, our optimized FAIMS-GPF XL-MS workflow includes a cross-linking step using MS-cleavable DSSO, a liquid digestion (Trypsin/LysC), and a fast automated peptide cleanup (*i.e*., fast sample preparation process; Fig. 1a), followed by an nLC-FAIMS-GPF-MS/MS with external CV stepping (Fig. 1b). This workflow was then evaluated and optimized on two samples of increasing complexity, the ∼110 kDa trimeric CAK complex and ‘native’ HeLa cell lysate, as described below.

### Using FAIMS-GPF XL-MS to extend structural biology insights into CAK complex

**Fig. 2:**
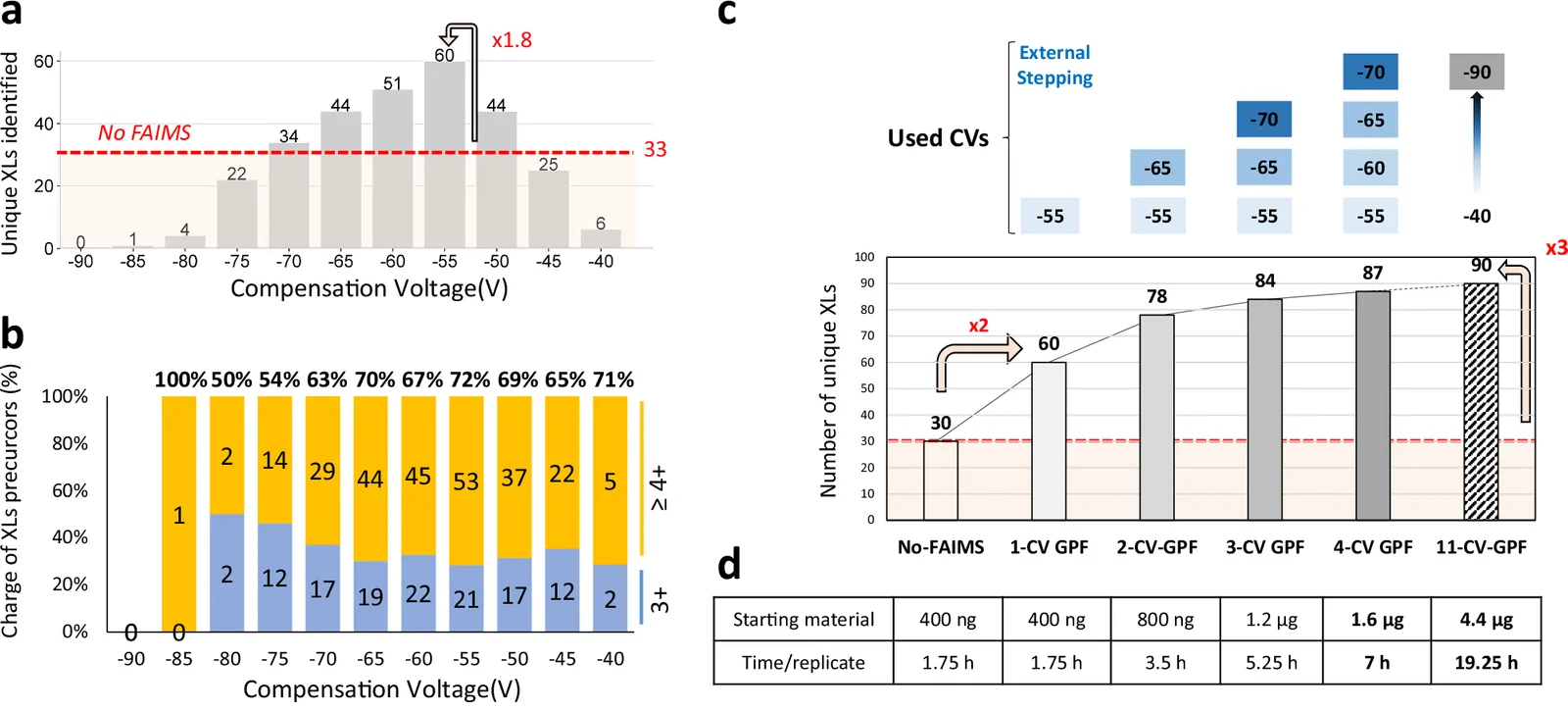
Screening of Compensation Voltage values on cross-linked CAK (5 V external stepping, *n* = 1 injection per CV). **a** Number of identified unique cross-links per CV value. **b** Charge repartition of cross-link spectrum matches (CSM) precursors per CV value. 3+ charges are shown in blue, and ≥ 4 + in orange, with the corresponding number of precursors indicated inside bars. The percentage of ≥ 4+ charges is indicated on top of the columns. **c** Number of unique XLs identified as a function of the number of CV-values chosen. **d** The table summarizes the total amount of CAK complex consumed and the associated time of LC-MS/MS analysis. Source data are provided as a Source Data file.

To evaluate the performance of FAIMS-GPF when applied to a low complexity system, *i.e*., a purified protein complex, we used the ∼110 kDa trimeric CAK (CdK7-Activating Kinase), composed of CDK7, MAT1 and Cyclin H. CAK is involved in the regulation of cell cycle and transcription processes either alone or as a component of the general transcription factor II H (TFIIH)^38–40^. As the first step, we screened different CV conditions to identify voltage values that yield the largest number of unique XLs. We pooled three independent XL reactions, each starting from 20 µg of CAK complex before digestion and peptide cleanup steps. We then directly analyzed the pooled sample using nanoLC-FAIMS-MS/MS (400 ng injected per CV run) with a gradient length of 105 min (90 min separation) to afford reasonable throughputs. The screening of CV values was done using external CV-stepping with steps of 5 V from −40–−90 V (11 CV values, Fig. 2a) and was compared to unfractionated XL-CAK. The FAIMS CV screening showed that single nanoLC-FAIMS-MS/MS runs with voltages ranging from −50–−70 V were all able to increase the total number of XL identifications. The highest number was obtained at −55 V (Fig. 2a), with 60 unique XLs identified (compared to 33 unique XLs for unfractionated CAK). This is most likely due to the “gas-phase enrichment” of ≥ 4+ charge states in the CV range −50–−70 V, with CV of −55 V yielding up to 72% of ≥ 4+ precursors (Fig. 2b). We evaluated the potential of cumulative external FAIMS-GPF steps using successive runs (n-CV GPF) and observed that although increase in unique XL pairs identified was evident even with 1-CV GPF, 4-CV GPF method allowed 97% coverage of all unique XL pairs, with that number changing insignificantly with further increase to 11-CV GPF (Fig. 2c). Thus, 4-CV GPF setup provides almost complete coverage of unique XL pairs while only requiring 7 h of instrument time and a total of 1.6 µg of starting material; it also almost triples the number of unique XLs identifications (x2.6) compared to analysis without FAIMS (Fig. 2d).

**Fig. 3:**
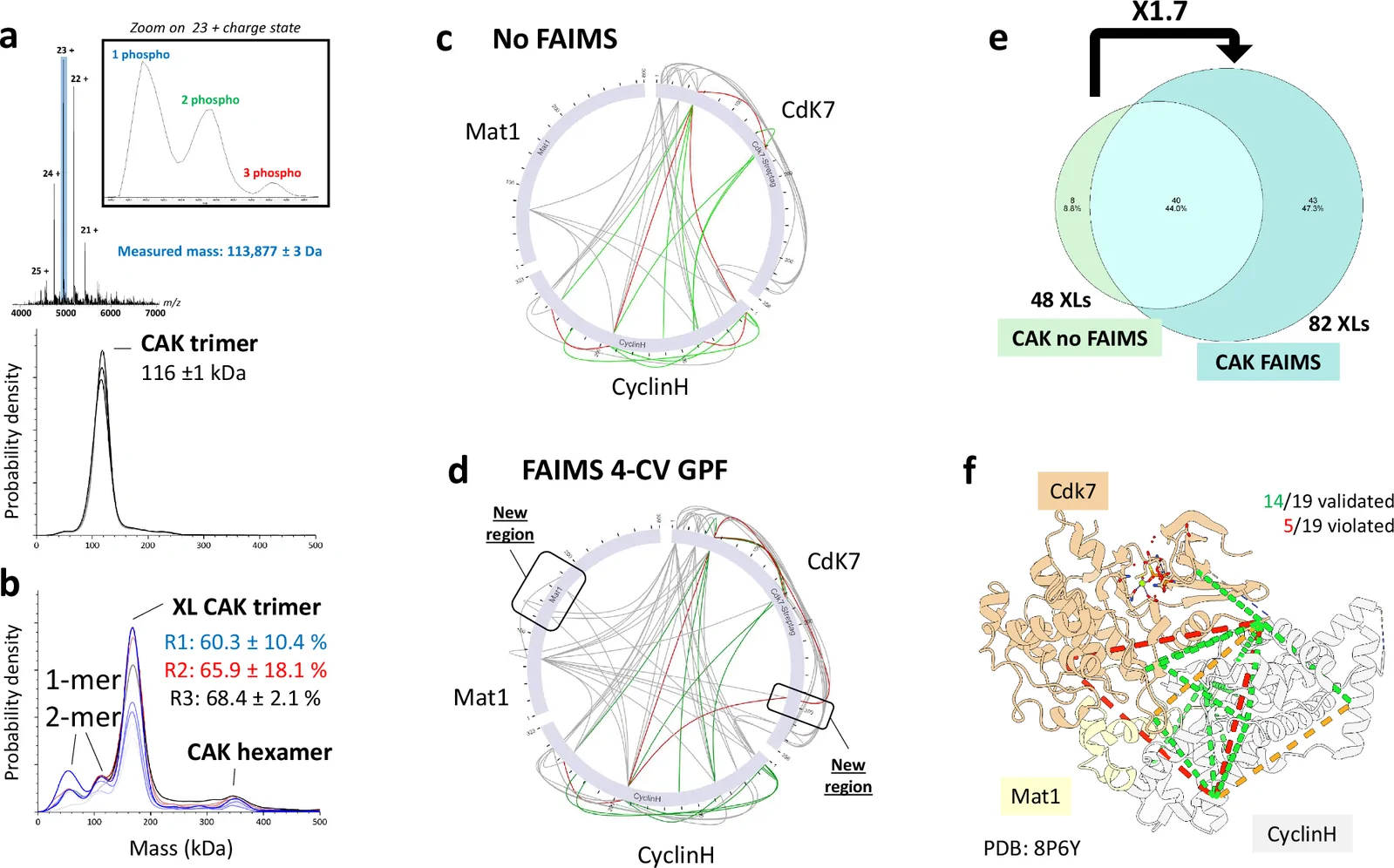
Comparison of XL-MS results in triplicate on CAK complex using classical method and FAIMS-4-CV GPF. **a** Quality control of the CAK sample before cross-linking reaction using native MS confirms the presence of the CAK trimer with 1 (113,877 ± 3 Da), 2 or 3 phosphorylations. nMP allows to quantify > 95% of intact CAK in the sample, with a measured mass of 116 ± 1 kDa. **b** dMP control of cross-linked CAK sample triplicate shows the stabilization of trimers, the co-existence of free monomers and sub-complexes, as well as a minor population of dimers of CAK heterotrimers. Measurement repeats of XL replicates 1, 2, 3 are color-coded respectively in shades of blue, black and red. Mass shift between theory and XL-CAK measured mass in dMP corresponds to the binding of mono-links and cross-links. Standard deviations are calculated from measurement repeats (*n* = 3). **c** Circular plot displaying the identified XLs on CAK partners’ sequences for CAK XL-MS experiments without FAIMS and **d** CAK XL-MS experiments with 4-CV FAIMS-GPF. Additional regions of interaction between are covered thanks to FAIMS-GPF in Mat1 and CdK7. **e** A Venn diagram shows the overlap of validated unique XLs (file threshold 2/3) in experiments without and with FAIMS. This allows increasing by 70% the number of XLs identified and identifying 43 new XLs. **f** We could map 19 XLs on the cryo-EM structure of CAK in complex with ATPgS (PDB: 8P6Y), out of which 14 were distance validated (74%) with a strict Cα-Cα threshold of 30 Å (highlighted in green). Five XLs were distance-violated (orange for 30–35 Å range and red for >35 Å).

We applied the 4-CV GPF protocol to a “real life” XL-MS experiment on the CAK complex in three independent XL replicates. We first performed native mass photometry (nMP) and native mass spectrometry (nMS) to assess the quality of our sample and checked the integrity of the CAK heterotrimer, before XL-MS experiments. Native MS revealed the presence of up to three phosphorylations on the CAK trimer (Fig. 3a), with the proteoform bearing a single phosphorylation as a predominant form (MW_exp_ = 113,877 ± 3 Da, MW_theo_ = 113,879 Da). nMP allowed us to further confirm the main species ( > 95% of sample) as intact CAK trimer (116.1 ± 1.0 kDa, Fig. 3a). To monitor the outcome of the DSSO XL reaction, we further used denaturing MP^41^ and confirmed that between 60 and 70% of CAK trimer was covalently stabilized in each XL replicates (Fig. 3b, Supplementary Fig. 3). Additional species were also identified corresponding to free monomers (16 ± 8%, 10 ± 8%, 7. ± 1% for XL replicates 1, 2, 3), dimers (14 ± 3%, 13 ± 11%, 16 ± 2% for XL replicates 1, 2, 3), as well as a low abundance of dimers of CAK trimer (5 ± 2%, 11 ± 2%, and 8 ± 1% for XL replicates 1, 2, 3). We next analyzed the XL replicates with the optimized 4-CV GPF method and compared it to analyses without FAIMS. Considering only unique XL pairs present in at least 2 out of 3 replicates (Supplementary Fig. 4), a final validated dataset of 48 XLs was obtained without FAIMS (Supplementary Table 1). The 4-CV GPF protocol identified 82 XLs (Supplementary Table 2). When comparing the two datasets, 40 XL pairs (44%) were found in common, while 43 XLs (47%) were specific to the 4-CV GPF protocol and only 8 XLs (9%) were uniquely found in the unfractionated run (Fig. 3c–e). Importantly, the 4-CV FAIMS-GPF protocol allowed us to identify additional interacting regions that have not been detected without FAIMS, such as interactions in the coiled coil domain of MAT1 (binding to ARCH) and the C-terminal of the kinase domain of CDK7 (Fig. 3d, Supplementary Fig. 5).

We also examined how well the XLs we identified using the 4-CV FAIMS-GPF protocol agree with the recently published cryo-EM structure of CAK (PDB: 8P6Y, Fig. 3f). Given that the density in the deposited CAK structure was incomplete, we could only map 19 (out of 82) XLs; nonetheless, the majority of the XLs we could map were distance validated with a strict Cα-Cα threshold of 30 Å (14 out of 19; 74%), while only five were distance-violated. Of these, the K52-S164 XL can be explained by the flexibility of the T-loop of Cdk7, which can adopt an alternative (inactive) conformation observed in the crystal structure of the isolated kinase^42^ and predicted in the AlphaFold model. The four remaining distance violations (Cdk7/K52-Cdk7/K291K; Cdk7/K291-Cyclin H/K189; Cdk7/K52-Cyclin H/K189; Cyclin H/K189-Cyclin H/K274) involve residues in regions well defined in the cryo-EM structure of CAK. As they cannot be explained by domain flexibility, they probably arise from alternative and transient conformations captured in XL-MS experiments, which were not selected during EM image selection and model building. Looking beyond the cryo-EM structure, in particular, we identified 24 interactions between Cdk7 and Cyclin H, which involve residues scattered over the entire cyclin H sequence and mostly in the N-terminal region of Cdk7. We also observed 11 XLs between pairs of residues that could be modeled from the X-ray structure of Cyclin H and Cdk7^42,43^. Interestingly, the XLs between Cdk7 and Cyclin H involve residues scattered over the entire Cyclin H sequence and mostly in the N-terminal region of Cdk7. These XLs are compatible with an AlphaFold-derived model^44,45^ obtained by docking the AlphaFold models of Cdk7 and Cyclin H (Supplementary Fig. 6) onto the cryo-EM structure of CAK (Supplementary Fig. 7). The XL dataset also contains 17 XL-pairs composed of one peptide from either Cdk7 or Cyclin H and one from MAT1. All detected XLs involve a residue from MAT1 located in the N-terminal part of the protein, mainly in the helical bundle, which shows that this region is able to fold back on the Cdk7/Cyclin H dimer (Supplementary Fig. 8). Therefore, although the cryo-EM structures of CAK^46,47^ does not include density for the N-terminal part of MAT1 due to the intrinsic flexibility of this region with respect to the rest of the CAK complex, our XL data were able to capture the transients interactions this domain makes with Cdk7/Cyclin H dimer.

Taken together, our 4-CV FAIMS-GPF workflow allowed us to map additional interactions and interfaces involved in the CAK complex. Importantly, the complete XL-MS experiment (done in triplicate) was carried out in only 3.5 days (21 h of instrument time) with only 4.8 µg of sample. Thus, FAIMS-GPF XL-MS provides a significant increase in sensitivity without requiring an increase in the amount of starting material consumed or instrument time.

### Proteome-wide FAIMS-GPF XL-MS experiments in whole cell lysates

**Fig. 4:**
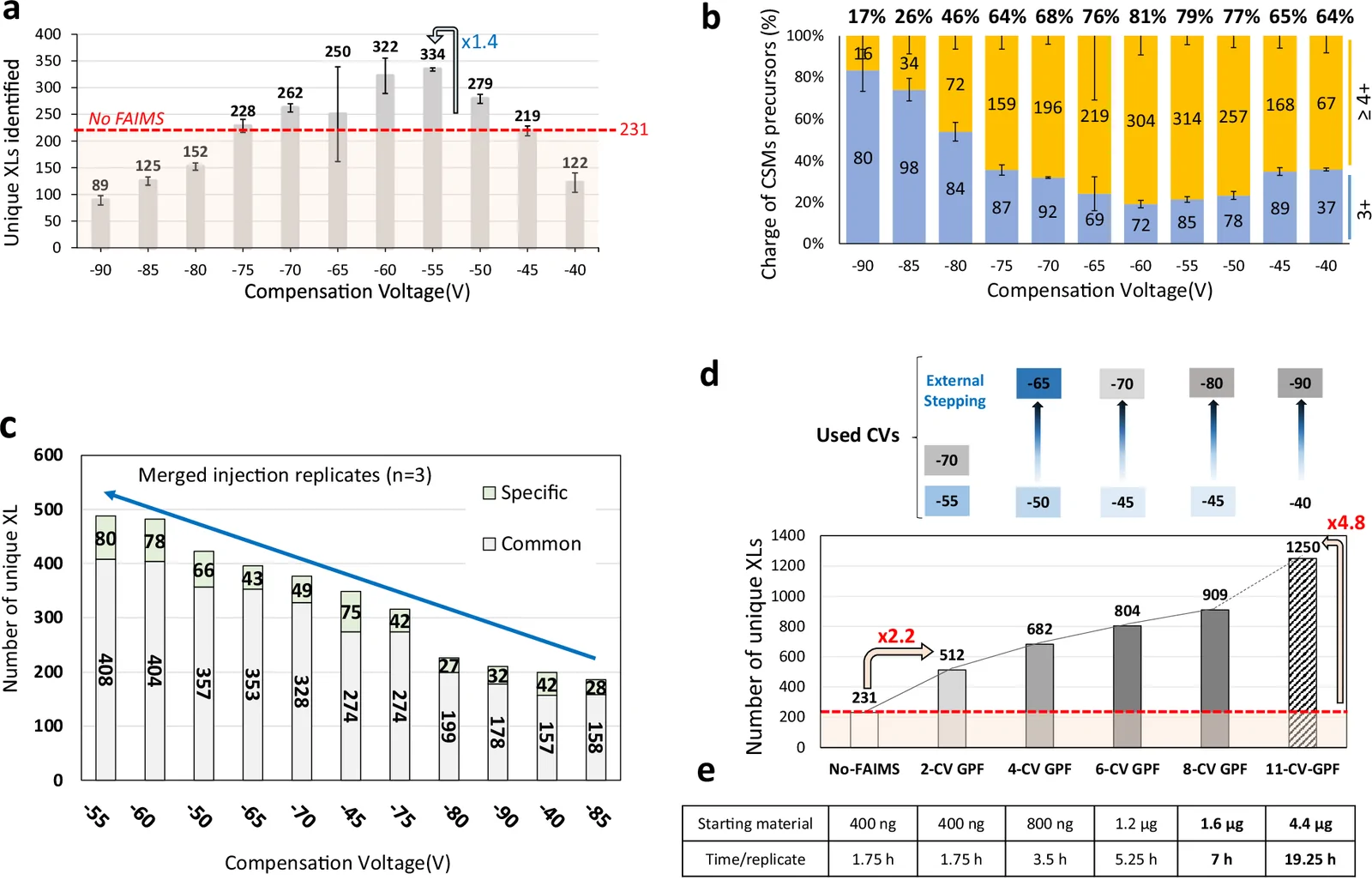
Evaluation of the FAIMS-GPF approach on cross-linked HeLa lysates. **a** Screening of CV values showing the number of identified unique cross-links per CV value. Average ± SD corresponds to injection replicates (*n* = 3). **b** Charge repartition of CSM precursors per CV value (*n* = 3 injections per CV) with the corresponding number of precursors indicated inside bars. Blue bars represent respectively 3+ and orange bars ≥ 4 + charge states. The percentage of ≥ 4 + charges states are written on top of bars. Values are shown as an average ± SD from injection replicates (*n* = 3). **c** According to the number of fractions needed (‘n-CV GPF’), CV values with the highest absolute number of unique XLs identified were successively associated, following the order indicated with a blue arrow (from merged injection replicates, *n* = 3). This order is closely related to the number of XLs specific to the CV value. **d** Evaluation of the number of gas-phase fractions needed for XL-HeLa lysate and **e** the potential throughput and sensitivity reached, using the first injection replicates. Source data are provided as a Source Data file.

We next evaluated the potential of using FAIMS-GPF XL-MS on a higher complexity sample, *i.e*., a whole-cell native HeLa lysate. As in the previous case study, we first optimized FAIMS-GPF conditions by testing a wide range of CV voltage values, displaying a similar charge-related filtering, and evaluating the optimal number of successive FAIMS-GPF runs (Fig. 4a–c, Supplementary Fig. 9). Conversely to the low complexity CAK sample, the number of unique XLs identified does not plateau after 4-CV GPF, and the increase is almost proportional to the number of gas-phase fractions used (*R*^*2*^ = 0.97, Supplementary Figs. 10, 11). Ultimately, we determined that a protocol with 11-CV FAIMS-GPF allowed identification of 1250 unique XLs, which is close to five times the number of unique XLs identified without FAIMS (Fig. 4d). To ensure the impact of our approach, we compared 11 injection replicates with 11-CV GPF, highlighting that improvement in identification comes mainly from GPF and not from replicate injections (Supplementary Fig. 12). This significant increase in XLs identifications was achieved using less than 1 day of instrument time and consuming as low as 4.4 µg of sample per sample to analyze (*e.g*., per XL replicates).

**Fig. 5:**
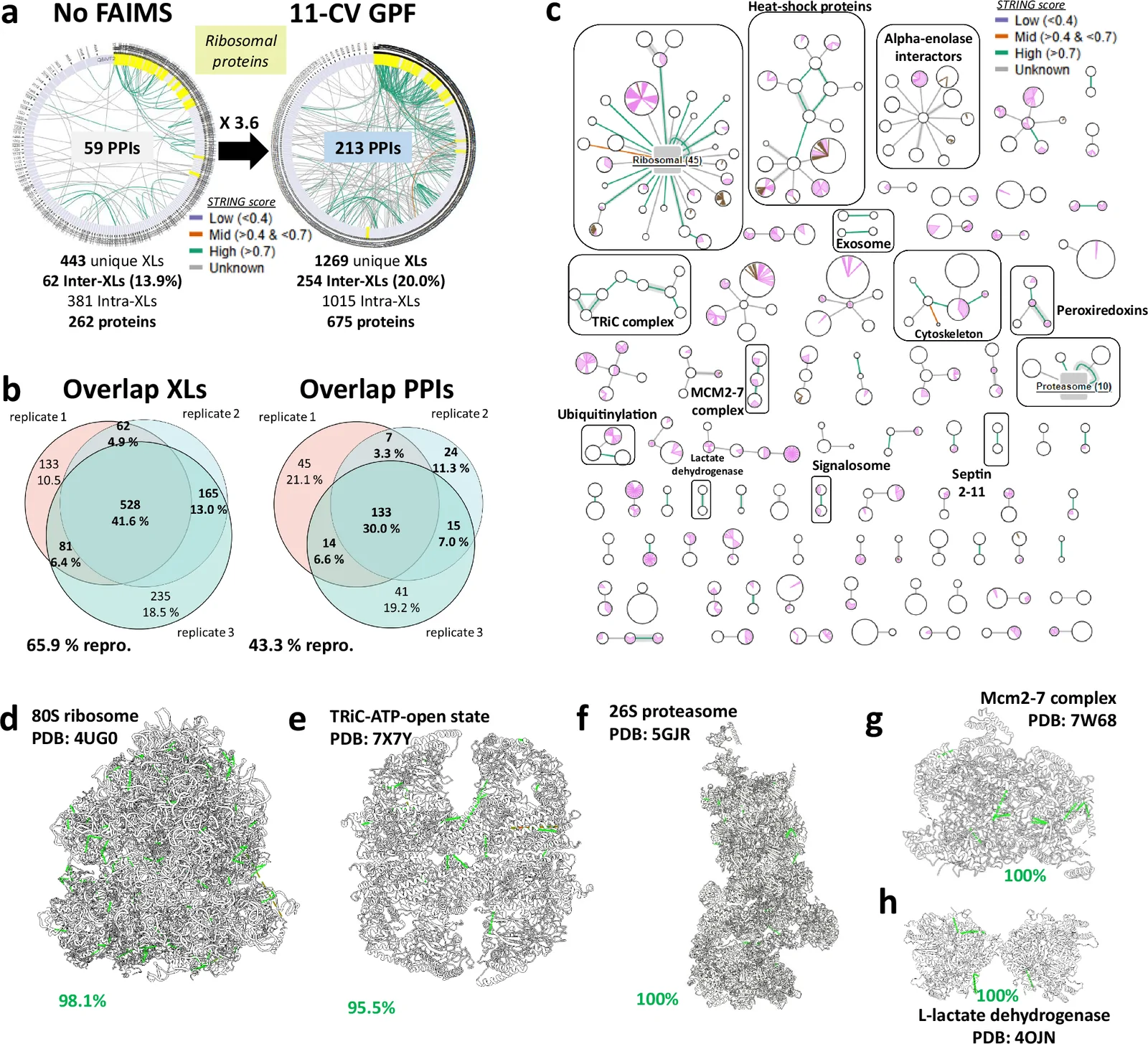
Results of XL-MS experiments using 11-CV FAIMS-GPF method on native HeLa Lysate cross-linked with DSSO in triplicate. **a** Circular plots comparing the inter-protein XLs identified from three independent XL replicates without FAIMS or with the 11-CV GPF method. A 3.6-fold increase in XLs identification is observed with FAIMS-GPF, leading to the identification of 1269 XLs in total, accounting for 213 PPIs. Ribosomal proteins, highlighted in yellow, are known to be a hot spot for PPIs. Interactions retrieved in the STRING database are highlighted in green if highly confident ( > 0.7) or orange if of lower confidence ( < 0.7). **b** Reproducibility (in ≥ 2/3 replicates) of unique XLs and PPIs identified in replicates (*n* = 3) is high, with respectively 65.9% and 43.3%. **c** PPI network obtained after 11-CV GPF displays 213 PPIs, where ribosome and proteasome are hotspots for interactions (both collapsed). PPIs from major complexes or important biological functions are circled in black. The size of nodes is proportional to protein length, with known domains (from Uniprot) highlighted in pink, as well as transmembrane domains highlighted in brown. Thickness of edges is proportional to the XL number for each PPI and color-coded according to their STRING score (green > 0.7, orange <0.7). Identified intra-protein and inter-protein XLs could be mapped on known 3D structures of different complexes with different sizes: **d** 80S Ribosome, **e** TRiC-ATP-open state, **f** 26S proteasome, **g** Mcm2-7 complex and **h** L-Lactate dehydrogenase. Distance-validated XLs falling within the 30 Å Cα-Cα range are displayed in green, violated XLs are displayed in red. Overall, 97.5% of XLs were distance-validated.

To highlight the benefits of our FAIMS-GPF approach, we carried out a complete XL-MS experiment by cross-linking three HeLa lysate replicates with 2 mM DSSO as a cross-linking reagent (Fig. 5). We compared the results of the 11-CV FAIMS-GPF protocol with the results obtained using unfractionated HeLa lysate. While our benchmark experiment performed without FAIMS identified 443 unique XLs pairs (381 intra-molecular, and 62 inter-molecular XLs representing 59 PPIs), the 11-CV FAIMS-GPF method allowed us to identify 1269 XL pairs (1015 intra-molecular and 254 inter-molecular XLs, representing 213 PPIs) (Fig. 5a). This represents a 2.8-fold increase in number of unique XLs identifications, with 915 new XLs, and a 3.6-fold increase in PPIs with 178 new PPIs identified compared to FAIMS-free analysis (Fig. 5a, Supplementary Fig. 13). Importantly, the reproducibility of the FAIMS-GPF was also higher than without FAIMS (65.9% vs. 43.3% without FAIMS, Fig. 5b, Supplementary Fig. 14).

To functionally annotate the unique XLs we identified, we performed a Gene Ontology (GO) enrichment analysis of the cross-linked proteins uniquely identified in FAIMS-GPF experiments (Supplementary Fig. 15). The enriched GO terms (*p* < 10E-20) corresponded to ‘cellular response to a stimuli’, ‘VEGFA/VEGFR signaling’, ‘60S ribosomal subunits’, ‘protein-RNA complex organization’, ‘regulation of transcription’, ‘neutrophil degranulation’, ‘carbon metabolism’, and ‘protein folding’. The highest number of PPIs was identified for ribosomal proteins (Fig. 5c; Supplementary Fig. 16a), 31 between ribosomal proteins and 21 between ribosomal proteins and other interactors, among which 8 were known interactions reported in the STRING database^48^ (*e.g*., RL23-EF2, RS12-SEBP1, RL31-BTF3) and 13 not previously known ones (*e.g*., RL4-FAM47E, RS7-PIGG, RS6-PEPD). We also observed several PPIs for proteasome subunits (Fig. 5c; Supplementary Fig. 16b). Additionally, we retrieved PPIs from other important complexes such TRiC (8 PPIs), MCM 2-7 complex (2), lactate dehydrogenase (1), signalosome (1), or Septin 2-11 (1), and interactions related to different biological processes or functions as ubiquitination (2), heat-shock proteins (15) or cytoskeleton (6) (Fig. 5c). Despite using a whole cell lysate, no interactions were found between “non-compatible” cell compartments. Among identified PPIs, 136 were present in the STRING database, and 86.7% of them were of very high confidence (scores > 0.9) (Supplementary Fig. 17b), highlighting the high degree of agreement between our data and previously reported PPIs. As a further quality check, we mapped MS-detected intra- and inter- XLs on available 3D structures for five selected complexes of different sizes (Fig. 5d–h). In total, 159 XLs were distance-validated at 97.5% across all complexes (Supplementary Fig. 17a).

Importantly, a significant portion of the PPIs we identified (118 out of 254) are not currently in the STRING database, suggesting that FAIMS-GPF XL-MS has the potential to discover direct interactions. For example, we identified a set of previously undocumented interactions involving alpha-enolase (ENO1) and major heat-shock proteins (Fig. 5c, Supplementary Fig. 18). ENO1, a glycolytic enzyme overexpressed in various cancers, is known to support tumor progression through metabolic reprogramming, proliferation, and immune evasion^49,50^. Our data revealed potential ENO1 interactions with ZNF521^51,52^ (a transcriptional regulator linked to cell differentiation), CKAP2^53^ and Coronin-2B^54^ (involved in cytoskeleton and actin dynamics), SNUT1^55^ (an RNA splicing factor), and membrane-associated proteins such as MYO1A^56^ and APLD1^57^. This suggests that ENO1 functions as a multifunctional hub bridging metabolism, gene regulation, and signaling in oncogenic contexts. In parallel, we identified potentially unknown interactions between HSPA1B, HSPA8, HSP90AB1, and HSPD1 with proteins like U2SURP^58^ (splicing), DIAPH1^59^ (cytoskeleton), and DNAJC13^60^ (vesicle trafficking). This highlights an expanded chaperone network that integrates proteostasis with broader cellular processes. Together, these findings reveal additional layers of functional connectivity for both ENO1 and heat shock proteins, providing a valuable resource for exploring their roles in cancer and cellular homeostasis. Although identified PPIs remain to be experimentally validated and functionally characterized in future studies, these results highlight the potential of FAIMS-GPF XL-MS as a tool for discovering biologically relevant interactions. One possible reason why we were able to identify uncharacterized PPIs may be the increased sensitivity of FAIMS-GPF compared to FAIMS-free experiments, as highlighted by label-free quantification (LFQ) experiments (Supplementary Fig. 19).

Taken together, the nanoLC-FAIMS-MS/MS workflow we developed was able to identify 1269 XLs and 213 PPIs overall (almost 3 times the number of XLs obtained without FAIMS) in less than 3 days of instrument time and using only 5.5 µg per XL replicate (for a total <20 µg). In comparison, similar performance using existing methods based on MS-cleavable reagents usually requires extensive in-solution fractionations ( > 20 fractions) using high amounts of starting material ( > 100 µg per sample, usually around 500 µg)^27,28,61–64^. Notably, our approach avoids in-solution fractionation in proteome-wide protocols while outperforming by fourfold the most sensitive workflow published up to now^65^. Therefore, the 11-CV FAIMS-GPF protocol achieved an unprecedented sensitivity and high reproducibility in a shorter time and using small amounts of starting material (as low as a few micrograms). This improved performance allowed us to identify potentially uncharacterized PPIs between important cellular proteins, suggesting that our method can be used to discover new aspects of cellular function.

### The 11-CV FAIMS-GPF protocol enables the study of challenging spliceosome complexes

Finally, we used the 11-CV FAIMS-GPF XL-MS method combined with MP on a challenging biological ribonucleoprotein sample, the spliceosome. During pre-mRNA splicing, the spliceosome removes non-coding introns and joins coding exons together to produce mature messenger RNAs (mRNAs)^66–69^. This megadalton-protein-RNA machinery ( > 200 partners) forms de novo on each intron and is subjected to extensive compositional and conformational transitions during the splicing process^35–40^. Although several studies have highlighted the value of XL-MS, especially when combined with cryo-EM, in providing critical insights into the assembly and function of the spliceosome^70–74^, purifying sufficient amounts of material for informative XL-MS studies remains a challenge. For example, our procedures routinely yield <40 µg of pre-catalytic spliceosome, which is not enough to perform extensive fractionations^28^ described for such samples and even worse to replicate XL reaction as recommended for XL-MS of purified complexes^75,76^. Thus, we examined whether the 11-CV FAIMS-GPF XL-MS method overcomes these limitations.

**Fig. 6:**
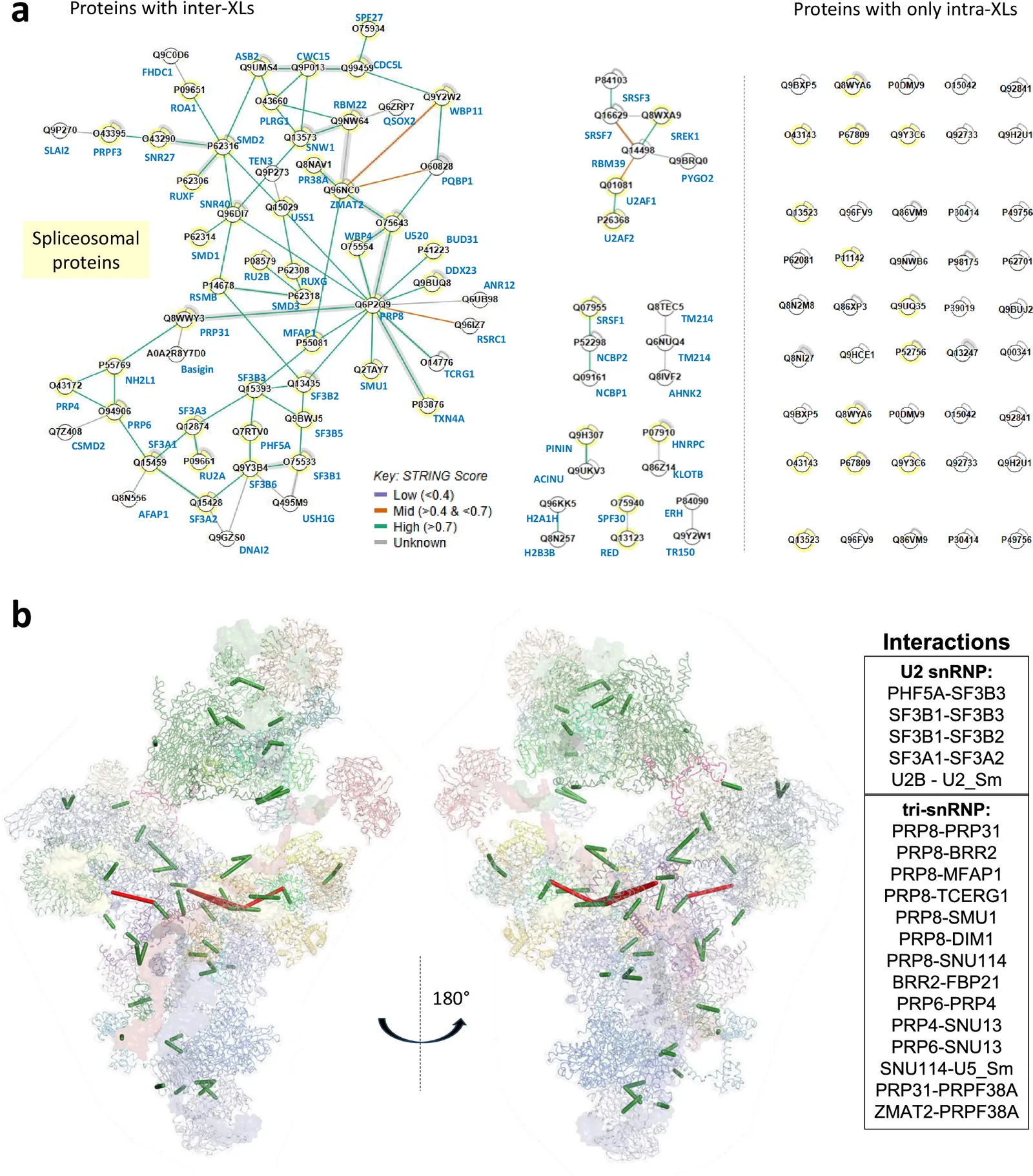
Results of XL-MS workflow with 11-CV FAIMS-GPF method to study spliceosomal protein interactions. **a** xiView network shows the 283 reproducible XLs identified in at least 2/3 replicates after 11-CV-GPF experiments. Nodes represent cross-linked proteins among which the ones from the spliceosome (Gene Ontology) are highlighted in yellow. Line thickness is proportional to the number of XLs and interactions found in the STRING database, and is color-coded in green for scores > 0.7 (confident) and in orange for scores <0.7. **b** Two different views of the human B-complex (PDB: 8QO9) show the spatial distribution of cross-links across the assembly. Intermolecular cross-links are mapped on the structure and highlighted as green (distance-validated) or red (distance-violated) lines according to Cα-Cα distance between cross-linked residues (<30 Å). Proteins involved in intermolecular cross-linking are shown in ribbon and colored according to the color scheme shown in the legend, snRNA are shown as a colored surface: U2 in green, U4 in yellow, U5 in blue and U6 in red respectively.

We verified sample integrity using nMP^77,78^, confirming the presence of intact RNA-bound spliceosomes (1749 ± 43 kDa) and two sub-complexes (1024 ± 124 kDa and 487 ± 13 kDa, Supplementary Fig. 20a, b). We split the sample into three and carried out the XL reaction in triplicate (11 µg each per reaction) using 1 mM DSSO. After a dMP^41^ control of XL reaction that confirmed the XL-stabilization of both intact spliceosome and sub-complexes (Supplementary Fig. 20c), samples were digested and cleaned-up as described in our general workflow overview (Fig. 1). Using the 11-CV FAIMS-GPF protocol, we could identify 231, 283 and 335 unique XL in replicates 1, 2 and 3, respectively, for a total of 405 XLs, *i.e*. a four-fold increase compared to the analysis without FAIMS (100 XLs in total; Supplementary Fig. 20d). Among them, we validated 283 unique XLs (file threshold 2/3, 4.6 times increase, 72% reproducibility) accounting for 91 PPIs including 110 proteins (Fig. 6a). We could map 55 XLs on the B complex structure (PDB: 8QO9), for which 93% of XLs were distance-validated (Fig. 6b, Supplementary Fig. 21). The cross-links that could not be distance-validated may be due to the co-existence of other intermediates, such as pre-B complex in the sample (Supplementary Fig. 22). We also identified several PPIs that are likely involved in interactions mediated by fragments of the B complex components that were not present in the cryo-EM density. Closer inspection of the putative position of such fragments further supports the presence of an additional 21 intermolecular cross-links on the B complex. Others detected PPIs occur between partners known to enter the splicing cycle later in the activation process (*e.g*., NineTeen Complex (NTC); also known as Prp19C)^79,80^, suggesting the presence of more activation-advanced spliceosome intermediates in this sample. Altogether, our fast and sensitive multiple-CV FAIMS-GPF XL-MS workflow is particularly well-suited for characterizing PPIs in complex spliceosome samples, enabling the detection of transient PPIs as well as providing a powerful tool to study sample heterogeneity.

## Discussion

All cellular processes depend on interactions between biomolecules, particularly proteins. Thus, hundreds of thousands of PPIs drive most aspects of cellular function, and deepening our ability to identify and study PPIs is essential for advancing biological research. Therefore, developing improved methods for detecting and identifying PPIs, especially transient PPIs formed by low-abundance proteins, remains important. In general, chemical crosslinking strategies are able to capture PPIs in the native environment and preserve physiologically relevant interactions, including those involving low-affinity complexes and low-abundance proteins. However, analyzing complex samples (such as cell lysates) that contain a mix of cross-linked and non-cross-linked peptides, with cross-linked peptides typically present in much lower concentrations, remains a major challenge and requires large amounts of starting material (>500 µg), extensive in-solution fractionation (>20 fractions), and increased instrument acquisition times (days to weeks). This can make XL-MS experiments impractical for samples like isolated organelles, nuclear extracts, specific cell lines or patient samples that are not available in such quantities. While several recent protocols achieve impressive depth in proteome-wide XL-MS, their applicability remains limited by several key factors: (i) the large amounts of starting material required, (ii) complex and highly sample-specific preparation procedures that demand significant expertise and optimization, (iii) reliance on specialized enrichable reagents that are not always commercially available or easily adaptable, and (iv) extended instrument times due to extensive in-solution fractionation and long chromatographic gradients.

Here, we addressed these challenges by developing an optimized XL-MS workflow based on FAIMS-GPF that reduces sample complexity at the MS level. This method represents a compromise between the amount of starting material required, the MS acquisition time, and the level of PPI detection. Overall, in all three case studies described above (purified trimeric CAK complex, HeLa cell lysate, and spliceosome), our optimized FAIMS-GPF XL-MS workflow resulted in increased sensitivity while starting with a few µg of sample and completing the triplicate experiments within 1 to 3 days. More specifically, applying 4-CV FAIMS-GPF on XL-MS CAK complex (1.6 µg per replicate) allowed deepening our understanding of PPIs by almost doubling the number of XL-IDs. At a proteome-wide level, the 11-CV FAIMS-GPF protocol using a total of 20 µg of starting material was able to provide 1269 XLs and 213 PPIs in less than 3 days, numbers that were previously only reached after extensive in-solution fractionation (*e.g*., using ≥10 to 20 chromatography fractions for a total of >100–500 µg of starting material and requiring days to weeks of sample prep and instrument time)^27,28,61,62,81^. Finally, our approach also outperforms by four-fold the most sensitive proteome-wide XL-MS protocol published up to now, which identified 307 XL-peptides from 5 µg of HeLa lysate using O-MICROFASP and tips-based SCX^65^. Therefore, our method represents an alternative to classical XL-MS workflows and provides a dramatic decrease in the amount of sample and instrument time needed, while retaining high sensitivity and reproducibility.

The improved sensitivity allowed us to make several important discoveries. For example, our analysis of the CAK complex led to the discovery that the N-terminal part of MAT1, mainly in the helical bundle, is able to fold back on the Cdk7/Cyclin H dimer. This interface mediated by the MAT1 N-terminal was not observed in reported cryo-EM structures of CAK because these structures were not able to capture this region of MAT1, given its high flexibility^46,47^. Although the interface and its functional role(s) remain to be analyzed and validated via orthogonal methods in future studies, these results indicate the effectiveness of our FAIMS-GPF XL-MS workflow to capture transient interactions involving flexible domains. Additionally, by analyzing the interactome of HeLa cell lysates, we were able to identify 118 PPIs that are not documented in the STRING database^48^ (*e.g*., interactions between ENO1 and different heat-shock proteins) that need further biological validation (out of the scope of this study).

We also benchmarked the improved speed and sensitivity of FAIMS-GPF for the XL-MS analysis of challenging samples, such as the spliceosome. Spliceosome is a large macromolecular assembly composed of hundreds of proteins and RNAs that are often purified in low quantities and with high heterogeneity. Here, we applied 11-CV FAIMS-GPF XL-MS with MP-based quality control steps of very high sensitivity. Using our protocol, we could perform an XL-MS experiment in triplicate from less than 40 µg of starting material within one week, which is almost impossible with more conventional XL-MS protocols. This is important because these amounts of starting materials (less than 40 µg) make the use of XL-MS fully compatible with modern structural biology (*e.g*., cryo-EM) workflows^75,76^.

Here, we show the efficiency and versatility of FAIMS-GPF XL-MS workflows. This versatile and easily tunable method uses low sample amounts and reduces instrument time, while ensuring compatibility with a broad range of samples. We thus expect the FAIMS-GPF method to facilitate a more straightforward integration of XL-MS workflows within structural biology efforts aiming at analyzing complex and dynamic systems (such as the spliceosome), and in routine use with proteomics platforms. Ultimately, this further boosts our ability to characterize the roles of protein-protein association networks interconnection in biological and cellular processes.

## Methods

### Sample preparation and cross-linking reactions of CAK complex

For production of the CAK ternary complex, the genes coding for the CDK7 kinase fused to a C-terminal Strep-tag II sequence, cyclin H, and MAT1 were cloned into pAC8_MF as described in^82^. Viruses were generated by co-transfection of the transfer vectors with the AcMNPV BAC10:KO1629, Δv-cath/chiA, mCherry bacmid and amplified using standard procedures^83^. Sf21 cells grown in suspension were infected with the corresponding virus, harvested after 3 days incubation at 27 °C, washed in PBS containing 10% v/v glycerol and flash frozen in liquid nitrogen. Typically, cells are grown in 2 L flasks containing 500 ml of medium and are infected at a density of 1.10^6^ cells/ml using 20 ml of P1 virus stock (assuming a titer of 5 × 10^7^ pfu/ml, it corresponds to a multiplicity of infection of 2).

For purification, the cell pellet from an infected suspension culture is disrupted by sonication in 20 mM Hepes-KOH, pH 8, 250 mM KCl, 0.1% Nonidet P40, 1 mM DTT and EDTA-free protease inhibitor cocktail (Roche). After centrifugation at 25,000 *g* for 20 min, the clarified lysate was incubated with Strep-Tactin®XT affinity chromatography (IBA-Lifesciences, 2-4030-010). After extensive washing with 20 mM Hepes pH8, 150 mM KCl, 1 mM EDTA, 1 mM DTT, bound proteins were eluted in the same buffer supplemented with 50 mM biotin. The CAK complex at 0.7 mg/mL in its native buffer was cross-linked in three independent replicates. 20 µg per replicate were incubated with DSSO freshly diluted in anhydrous DMSO to reach 200 molar excesses (45 min, RT). Reaction was quenched with 20 mM Tris-HCl for 20 min.

CAK complex at 0.7 mg/mL in its native buffer (20 mM Hepes pH 8, 150 mM KCl, 1 mM EDTA, 1 mM DTT, 50 mM Biotin) was cross-linked in three independent replicates. 20 µg per replicate were incubated with DSSO freshly diluted in anhydrous DMSO to reach 200 molar excesses (45 min, RT). Reaction was quenched with 20 mM Tris-HCl for 20 min.

### Sample preparation and cross-linking reactions of spliceosome

The spliceosome sample was obtained by assembling spliceosomes from a nuclear extract previously treated 10 min at 30 °C with 1 μM cyclin-dependant kinase inhibitor OTS964^84^ onto an AdML-3xMS2 substrate for 1 h at 30 °C essentially as described previously^66^. Assembled spliceosomes were purified by glycerol cushion, amylose affinity and glycerol gradient ultracentrifugation. In summary, 60 mL of splicing reaction was diluted by the addition of 60 mL of dilution buffer K150 (20 mM HEPES-KOH pH 7.9, 40 mM KCl, 0.25 mM EDTA, 1% glycerol, 1 mM DTT, 0.03% NP40). The resulting diluted splicing reactions were loaded onto 9 ml of 40% glycerol cushion and ultra-centrifuged at 32,000 rpm in SW32 Beckmann (20 mM HEPES-KOH pH 7.9, 50 mM KCl, 0.25 mM EDTA, 40% glycerol, 1 mM DTT, 0.02% NP40). Cushions were then recovered, diluted in 3.0 mL of D150K (20 mM HEPES-KOH pH 7.9, 150 mM KCl, 0.25 mM EDTA, 1% glycerol, 1 mM DTT, 0.03% NP40) and incubated with amylose resin high flow (NEB) overnight at 4 °C. After extensive washing with WK150 (20 mM HEPES- KOH pH 7.9, 150 mM KCl, 0.25 mM EDTA, 1% glycerol, 1 mM DTT, 0.01% NP40) the MS2-containing complexes were eluted using WK150 supplemented with 12 mM of maltose. The fractions containing the complexes, as judged by fluorescein signal, were pooled, precipitated with PEG20K, resuspended in buffer W150K and loaded onto 10–30% glycerol gradients (20 mM HEPES- KOH pH 7.9, 150 mM KCl, 0.25 mM EDTA, 10% or 30% glycerol, 1 mM DTT, 0.01% NP40) and spun for 3 h at 50000 rpm in SW60 rotor, gradients were fractionated from the bottom to the top and the fluorescent signal measured and plotted against the number of fractions. The B-like complex peak fractions were pulled together and concentrated in 100 KDa to reach the concentration of roughly 1 mg/ml (500 nM).

Three 11 µg aliquots of spliceosome were independently cross-linked with 1 mM DSSO (45 min, RT), and quenched with 20 mM Tris HCl for 20 min.

### Native mass spectrometry measurements of CAK complex

CAK trimer was buffer-exchanged against 150 mM ammonium acetate using Zeba Spin desalting columns (Thermo Fisher Scientific, Rockford, IL, USA). The sample was analyzed in positive ion mode on an Orbitrap Exactive Plus EMR (Thermo Fisher Scientific, Bremen, Germany) coupled to a nano-electrospray source (TriVersa NanoMate, Advion Biosciences, Ithaca, U.S.A.), piloted by Xcalibur. The following parameters were used: resolution of 17500, in-source collision-induced dissociation (CID) of 150 eV, Collision Energy of 70 eV, trapping pressure of 7 (u.a.). The injection, inter- and bent-flat apoles were set to 4 V. Data were analyzed using Xcalibur Qual Browser and UniDec.

### Native and denaturing mass photometry measurements of CAK and spliceosome complexes

MP measurements were performed with a TWO^MP^ (Refeyn Ltd, Oxford, UK) at room temperature (18 °C). Microscope slides (24 × 50 mm, 170** ±** 5 µm, No. 1.5H, Paul Marienfeld GmbH & Co. KG, Germany) were cleaned with milli-Q water, isopropanol, milli-Q water and dried with a clean nitrogen stream. Six-well reusable silicone gaskets (CultureWell^TM^, 50–3 mm DIA x 1 mm Depth, 3–10 µL, Grace Bio-Labs, Inc., Oregon, USA) were carefully cut and assembled on the cover slide center. After being placed in the mass photometer and before each acquisition, an 18 µL droplet of PBS was put in a well to enable focusing on the glass surface.

For native MP, samples were analyzed at 50 nM in a PBS droplet. Denaturing MP experiments were carried out as described previously^41^. Denaturation was done as described previously^41^, by incubating samples in 5.4 M Urea (Sigma, Saint-Louis, USA) for 5 min at 90 °C. Samples were drop-diluted to 50 nM and measured in a PBS droplet. Three movies of 3000 frames were recorded (60 s) for each sample using the AcquireMP software (Refeyn Ltd, Oxford, UK). A contrast-to-mass calibration was performed twice a day by measuring a mix of Bovine Serum Albumin (66 kDa), Bevacizumab (149 kDa), and Glutamate Dehydrogenase (318 kDa) in PBS buffer, pH 7.4. Data were finally processed using the DiscoverMP software (Refeyn Ltd, Oxford, UK).

### Sample preparation and cross-linking reactions of HeLa Lysate

For ‘native’ cell lysis, live HeLa cells were washed with PBS, pelleted with ultracentrifugation and frozen at −80 °C overnight. Pellet was unfrozen on ice in 500 µL of a non-denaturing buffer containing 20 mM Hepes, 150 mM NaCl, 1.5 mM MgCl2 and 0.5 mM DTT, adjusted at pH 7.8. A tablet of EDTA-free protease inhibitor cocktail was added per 10 mL of buffer. Cells were mechanically lysed on ice with 40 quick pushes using a 27¾ Gauge syringe. Cell debris was further removed using a 13,800 G centrifugation for 10 min at 4 °C. Supernatant protein concentration was measured at 1.4 mg/mL using the Pierce assay. Concentration was adjusted at 1 mg/mL, and 3 aliquots of 100 µg of lysate were cross-linked with a final concentration of 2 mM of DSSO (45 min, RT), followed by a quenching step with 20 mM TrisHCl. For FAIMS-GPF CV screening 50 µg of each XL replicate were pooled. For final FAIMS-GPF XL-MS experiments a 20 µg aliquot was taken for each independant XL replicate.

### SDS-PAGE separation of cross-linked samples

All cross-linked proteins and complexes were migrated to check the efficiency of XL reaction on in-house 12% acrylamide denaturing SDS-PAGE gels (1.5 mm thickness). Volume corresponding to 1.5 µg of each XL sample (and non-XL controls) was diluted (1:1) with 2x concentrated Læmmli buffer (4% SDS, 20% glycerol, 10% 2-mercaptoethanol, 0.01% bromphenol blue and 0.125 M Tris HCl) and incubated 5 min at 95 °C. After sample loading, gels were migrated at 50 V for 20 min, 100 V for 2/3 of the gel and 120 V until the end. After migration, gels were fixated for 20 min (3% phosphoric acid, 50% ethanol), washed 3 x 20min with milli-Q water and stained overnight with Coomasie Brilliant Blue (G250, Sigma, Saint-Louis, USA). They were finally rinsed 3 × 20 min with milli-Q water.

### Proteolysis of samples and automated cleanup of peptides

All samples were denatured (6 M Urea), reduced (5 mM DTT, 30 min at 37 °C) and alkylated (15 mM Iodoacetamide, 1 hin the dark, RT). After dilution to <2 M Urea using Ammonium Bicarbonate, aliquots were digested overnight at 37 °C with Trypsin/LysC (Promega, Madison, USA) at a 25:1 enzyme:protein (w/w) ratio for XL-lysate and 50:1 for CAK. Digestions were quenched with 1% TFA. Peptides generated by proteolysis were cleaned up using the AssayMAP Bravo platform (Agilent Technologies; Santa Clara, California) with 5 μL C18 cartridges (Agilent). Briefly, cartridges were primed (100 μl 0.1% TFA in 80% CAN), equilibrated (50 μl 0.1% TFA in water), and 180 μl of digested peptides diluted in equilibration buffer were loaded on the cartridges, and finally cleaned with 50 μl of equilibration buffer. Peptides were eluted with 50 µl 0.1% TFA in 80% ACN. Eluted peptides were frozen and kept at −80 °C until analysis.

### nLC-MS/MS proteomics experiments on HeLa lysates digests

Analysis were done using a Dionex UltiMate 3000 RSLC nano system (Thermo Scientific) coupled to an Orbitrap Eclipse Tribrid (Thermo Scientific). We used a mobile phase A (0.1% FA in water) and mobile phase B (0.1% FA in 80% ACN/20% Water). 400 ng of peptides were injected and first trapped on an Acclaim PepMap 100 C18 20 mm × 0.1 mm, 5 µm diameter particles pre-column (Thermo Scientific) at 10 µL/min for 3 min. Separation was done at 300 nL/min on an Aurora Series C18 UHPLC column (250 mm × 75 µm, 1.6 µm diameter particles from IonOpticks) with the following gradient: 2.5% B for 3 min, 32.2% B at 53 min, 50% B at 63 min, 98.0% B at 65 min, 98.0% B kept until 70 min, 2.5% B at 71 min. Analysis were carried out in Data Dependent Acquisition (DDA) with a 2 s cycle, full MS spectra were acquired over a 300–1800 m/z range at a resolution of 120000 in the Orbitrap, with a standard AGC target and an Auto max. injection time. For MS/MS, the intensity threshold was set at 5e3, and 2–7+ charges were selected for Higher Collisional Dissociation (HCD) using respectively 30% Normalized Collision Energy (NCE). MS/MS spectra were acquired in the Ion Trap with a Rapid scan rate and normal AGC target and an Auto max. injection time of 200 ms. MaxQuant was used to process proteomics experiments on lysate using standard parameters. To compare protein abundances, the mean of the LFQ value was calculated between replicates, ignoring missing values.

### nLC-MS/MS of cross-linked samples without FAIMS

For nanoLC-MS/MS/ analysis, cross-linked peptides were dried in a SpeedVac concentrator and resuspended in 2% ACN/0.1% formic acid. Analysis were done using a Dionex UltiMate 3000 RSLC nano system (Thermo Scientific) coupled to an Orbitrap Eclipse Tribrid (Thermo Scientific). We used a mobile phase A (0.1 FA in water) and mobile phase B (0.1% FA in 80%ACN/20%Water). 400 ng of peptides were injected and first trapped on an Acclaim PepMap 100 C18 20 mm × 0.1 mm, 5 µm diameter particles precolumn (Thermo Scientific) at 10 µL/min for 3 min. Separation was done at 300 nL/min on an Aurora Series C18 UHPLC column (250 mm × 75 µm, 1.6 µm diameter particles from IonOpticks) with the following gradient for CAK: 2.5% B for 3 min, 43.8% B at 93 min, 98.0% B at 95 min, 98.0% B kept until 100 min, 2.5% B at 102 min. The following gradient was used for XL-lysates: 2.5% B for 3 min, 43.8% B at 123 min, 98.0% B at 125 min, 98.0% B kept until 130 min, 2.5% B at 132 min. Analysis were carried in Top10 Data-Dependent Acquisition (DDA), full MS spectra were acquired over a 300–1800 m/z range at a resolution of 120000 in the Orbitrap, with an AGC target of 750% and a 100 ms max. injection time. For MS/MS, the intensity threshold was set at 2.0e5, and only 3–7+ charges were selected for Higher Collisional Dissociation (HCD). HCD was applied in Stepped Collision Energy mode using respectively 21, 26, 31% Normalized Collision Energy (NCE). MS/MS spectra were acquired at 60000 resolution (1.6 m/z isolation window) in the Orbitrap using an AGC target of 400% and a max. injection time of 200 ms.

### nLC-FAIMS-MS/MS gas-phase fractionation experiments for cross-linked samples

For all samples, nanoLC separation 90 min separation/105 min gradient was used (see above for CAK complex). MS analyses were carried out in Top10 DDA, with full MS spectra acquired over a 300–1800 m/z range at a resolution of 120000 in the orbitrap, with an AGC target of 250% and a 100 ms max. injection time. For MS/MS, the intensity threshold was set at 2.5e4, and 3+ to 7+ charge states were selected for Higher Collisional Dissociation (HCD). HCD was applied in Stepped Collision Energy mode using 21, 26 and 31% Normalized Collision Energy (NCE). MS/MS spectra were acquired at 60000 resolution (1.6 m/z isolation window) in the Orbitrap using a standard AGC target, an auto max. injection time and a dynamic exclusion time of 60 s. For the FAIMS setting, gas mode was set as static with a total carrier gas flow of 4.6 L/min and a standard FAIMS resolution. A single Compensation Voltage was used per single nLC-FAIMS-MS/MS run. For CV screening CV values used were: −40 V, −45 V, −50 V, −55 V, −60 V, −65 V, −70 V, −75 V, −80 V, −85 V, −90 V. For 4-CV GPF analysis of XL-CAK, −55 V, −60 V, −65 V, and −70 V were used; for 11-CV GPF analysis of XL-lysates and spliceosome, all CVs from -40–−90 V were used.

### Data processing

For data processing,.raw data were directly processed with Thermo Proteome Discoverer 2.5.0.400 (Thermo Scientific) using the Sequest HT node for the identification of peptides and the XlinkX node for identification of crosslinks. For CAK, a database containing only proteins of interest was used, and for proteome-wide samples, a database containing the full human proteome was used (retrieved on 2023/07/09). Cysteine carbamidomethylation was set as a fixed modification, and Methionine oxidation, N-term acetylation, tris-quenched mono-links and water-quenched mono-links were set as dynamic modifications. Trypsin was set as the cleavage enzyme with a minimal length of 7 amino acids, 2 (linear peptides) and 3 (cross-linked peptides) missed cleavages were allowed, respectively, for proteomics and XL identifications. For CAK, we considered K, S, T and Y as DSSO linkage sites due to the relatively low number of spectra allowing manual validation. As recommended^28,63^, due to the high number of spectra and to avoid false positives^85,86^, only K was considered for spliceosome and proteome-wide samples. Mass accuracies for both XL and linear peptides search were set to 10 ppm for MS1 and 20 ppm for MS2^28,63^. To increase confidence, identifications were only accepted for Maximal XlinkX scores > 40 and Δ_score_  >  4. A 1% false discovery rate was applied for both linear and XL-peptides at CSM (separated FDR between intra and inter-XLs), at the peptides and proteins level. All FAIMS-GPF XL-MS experiments on CAK, HeLa lysate and Spliceosome, including CV screening, have been deposited to the ProteomeXchange Consortium via the PRIDE^87^ partner repository with the dataset identifier PXD064228.

GO enrichment analysis was done by analyzing cross-linked proteins only identified in proteome-wide FAIMS-GPF experiments in the Metascape online server^88^. XL-MS data were visualized using the xiVIEW webserver (www.xiview.org)^21^, to produce circular interaction networks and represent XL sites on protein sequences. Finally, validated cross-links were plotted against different structures using the PyMol Molecular Graphics System (version 2.5.4, Schrödinger, LLC), ChimeraX as well as the xiVIEW server to visualize and measure XLs Cα-Cα distances on the structure. Corresponding distances were validated if satisfying a cutoff of 30 Å^28,62,89^.

### Reporting summary

Further information on research design is available in the Nature Portfolio Reporting Summary linked to this article.

## Supplementary information

Supplementary Information

Reporting Summary

Transparent Peer Review file

## Source data

Source Data

## Data Availability

Complete FAIMS-GPF XL-MS dataset including CV screening on CAK and HeLa lysate, as well as the triplicate experiments on CAK, HeLa and Spliceosome have been deposited to the ProteomeXchange Consortium via the PRIDE^87^ partner repository with the dataset identifier PXD064228. Processed dMP results have been included in the ProteomeXchange submission. Source data are provided with this paper.
