# Supplementary material for: FAIMS-GPF XL-MS: crosslinking-mass spectrometry based on gas-phase fractionation
Source: Nat Commun. 2026 Jul 23;17:8985. doi: 10.1038/s41467-026-75736-9 (PMC13507205; doi:10.1038/s41467-026-75736-9)
Supplement: Supplementary file 1 [file 41467_2026_75736_MOESM1_ESM.pdf]

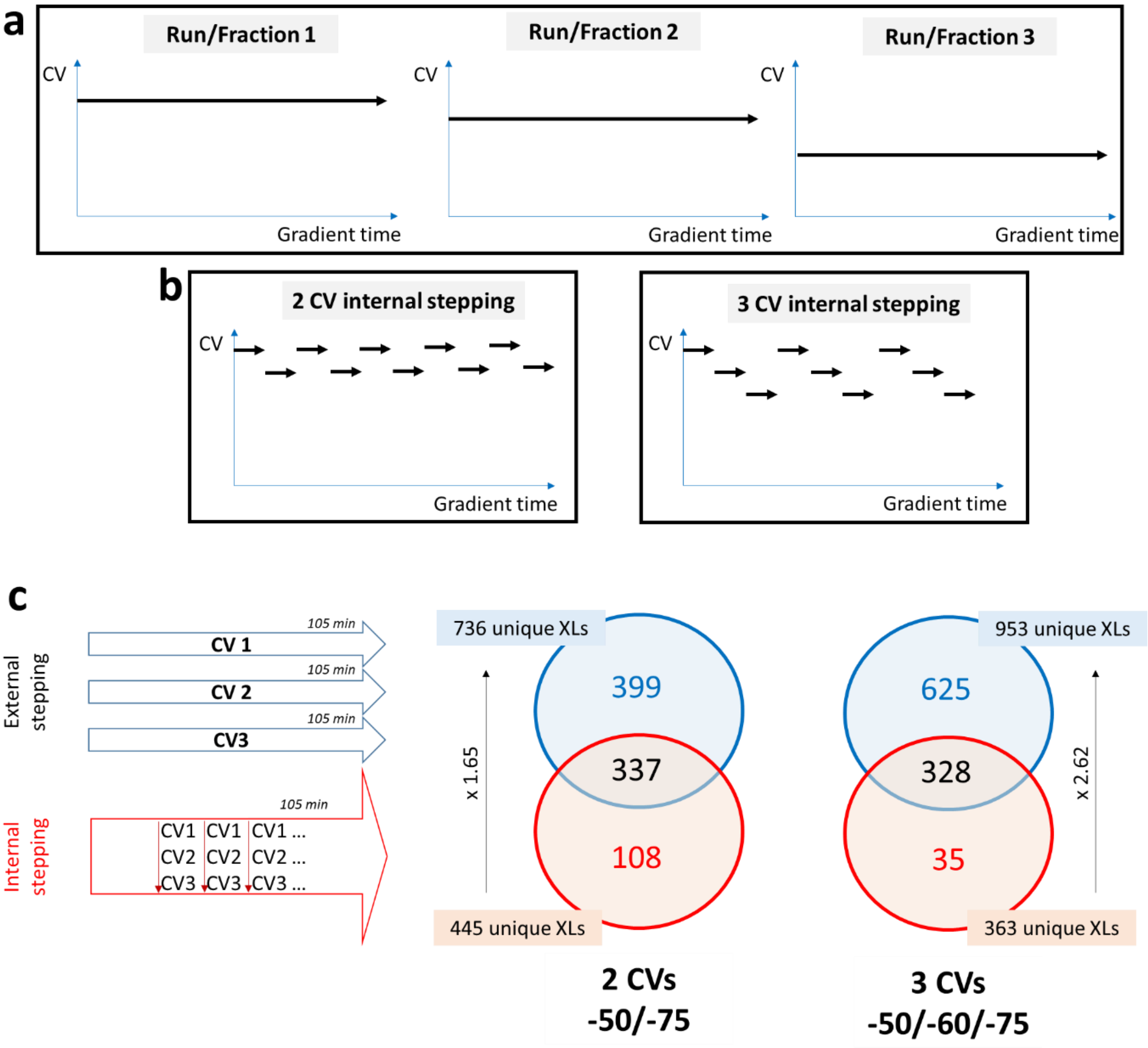

Supplementary Figure 1. Comparison of FAIMS external and internal stepping on an strong cation exchange (SCX)-enriched XL-Hela lysate (fraction number 6/6). (a) Description of external stepping GPF experiments. **(b)** Description of internal stepping GPF experiments. **(c)** We first compared internal and external stepping for 2 (-50/-75V) and 3 (-50/-60/-75) CV values on a DSSO cross-linked HeLa lysate. We used a 105 min gradient and found that external stepping allowed 1.6 and 2.6 times more unique XLs identification for 2 and 3 CVs respectively, than internal stepping. As the sampling of precursors across gradients is poor with external stepping, many precursors will only be submitted to 1 CV value.

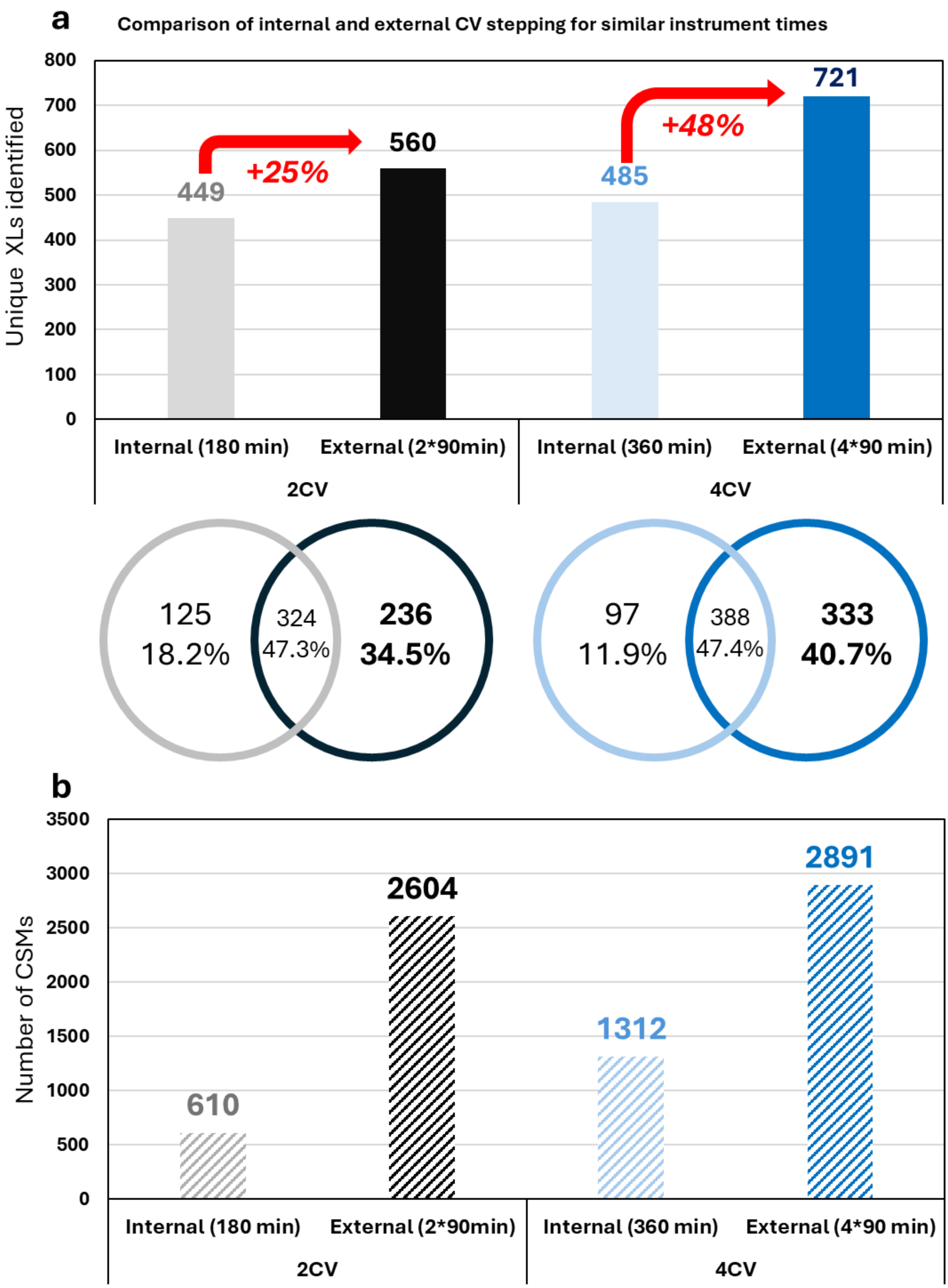

Supplementary Figure 2. Comparison of FAIMS external and internal stepping on a strong cation exchange (SCX)-enriched XL-Hela lysate (fraction number 6/6). (a) Comparison of unique XLs identified, **(b)** Comparison of CSMs number.

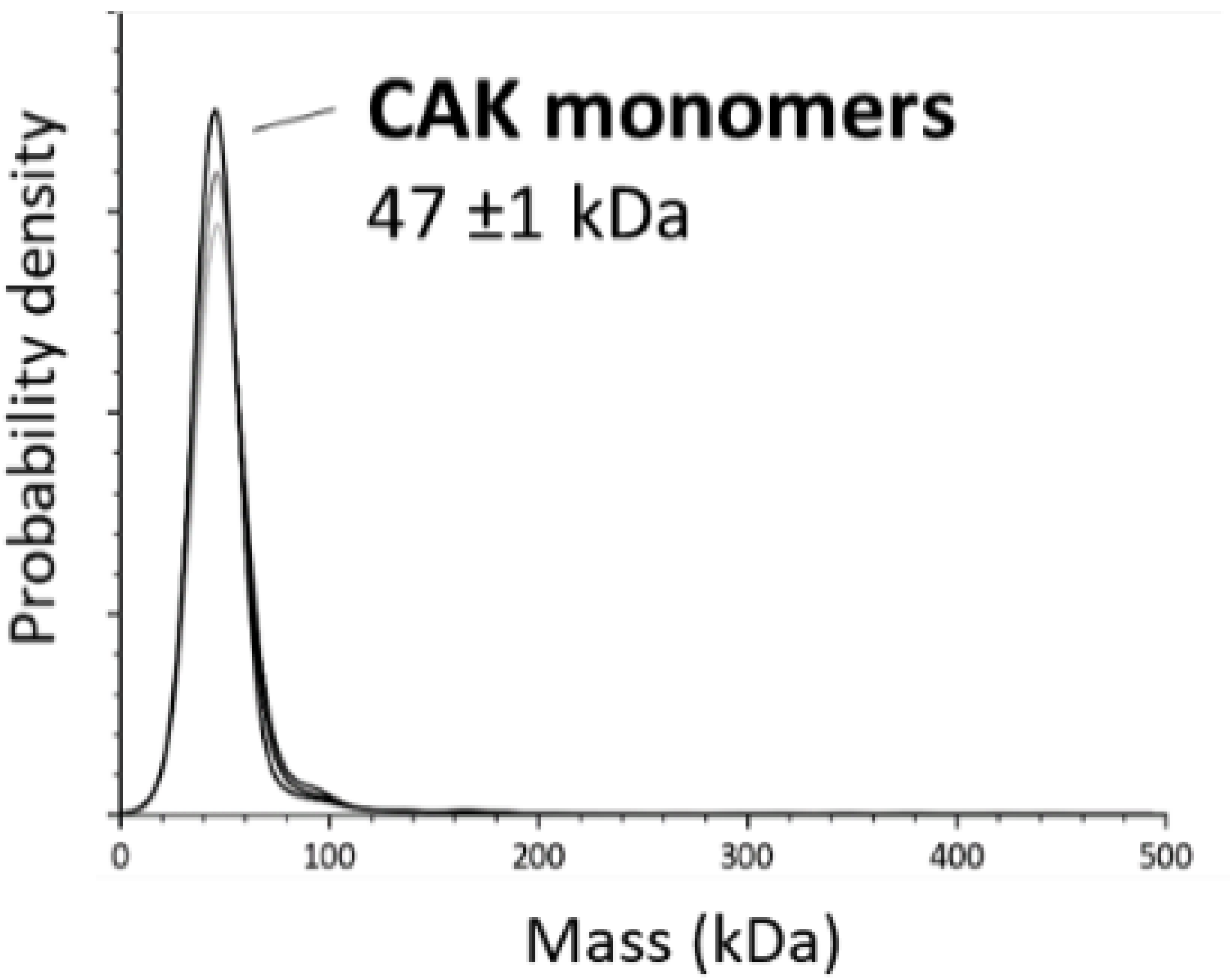

Supplementary Figure 3. Denaturing MP of non-XL CAK control. Mass shift between theory and XL-CAK measured mass in dMP corresponds to binding of mono-links and cross-links. SD comes from independent measurement (n=3).

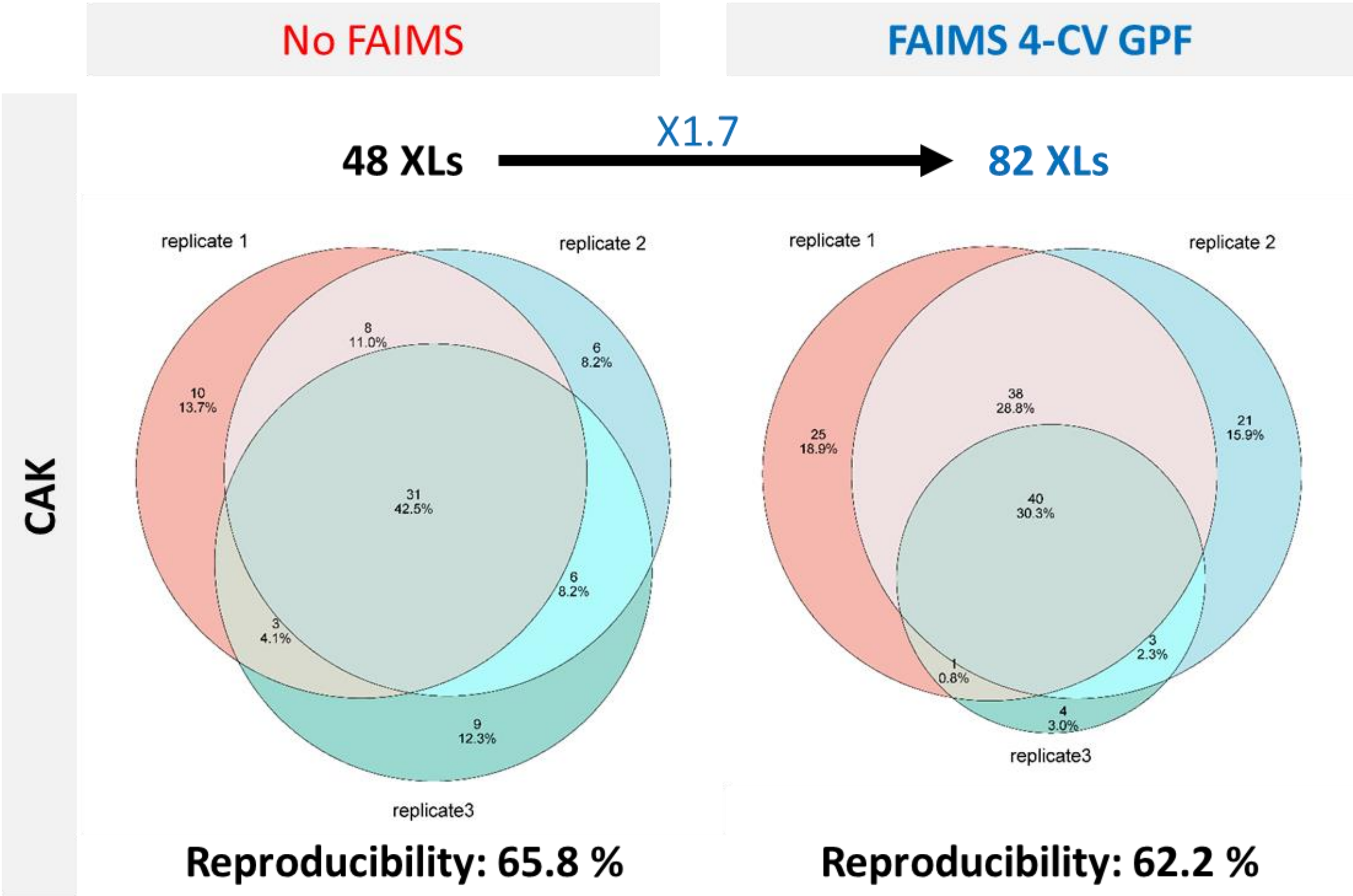

Supplementary Figure 4. Reproducibility between independent XL replicates for XL-MS experiments on CAK without and with FAIMS.

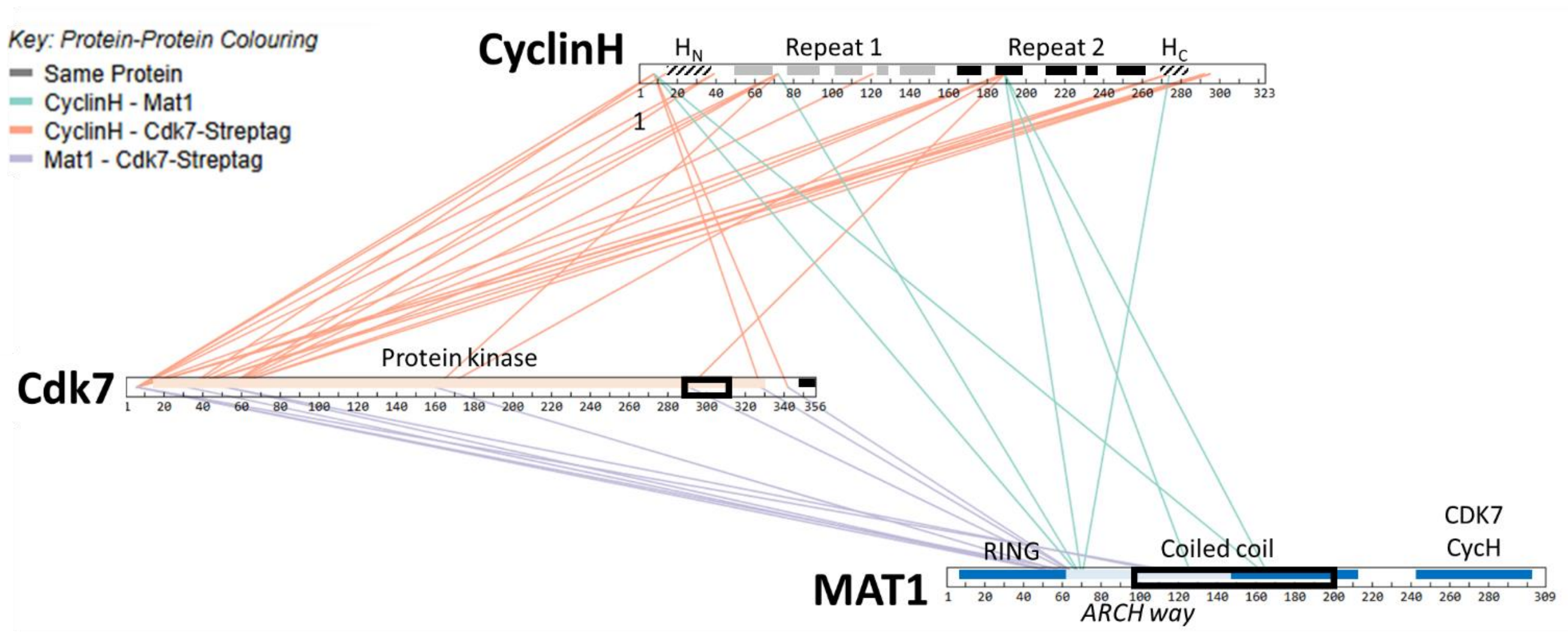

Supplementary Figure 5. Validated inter-protein XLs from the 4-CV GPF experiments mapped on the CAK partners sequences. The different domains of protein partners are indicated. Regions of Cdk7 and MAT1 only covered using FAIMS-GPF are circled with black rectangles.

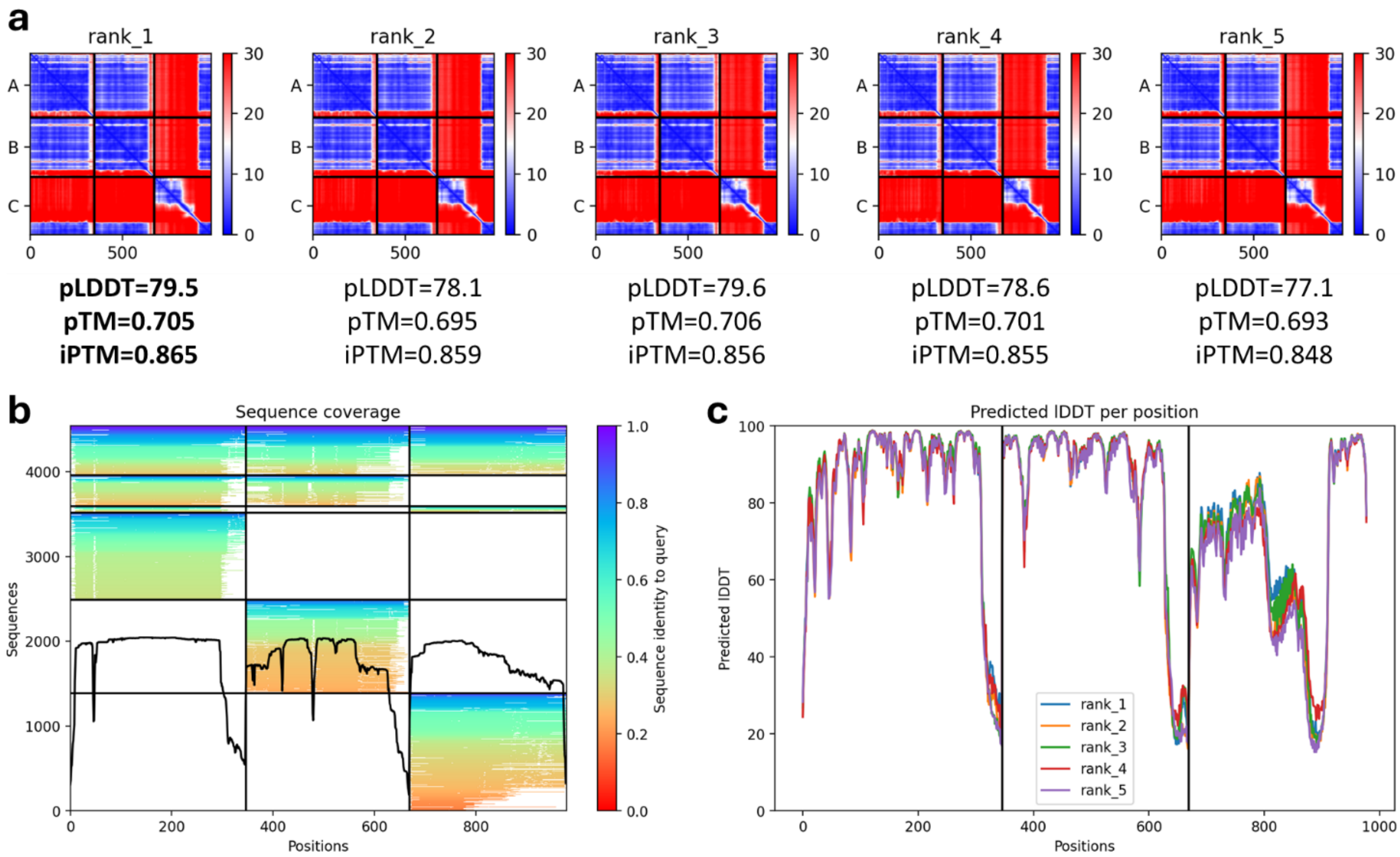

Supplementary Figure 6. AlphaFold prediction of CAK complex using CollabFold2 (alphafold2_multimer_v3). **(a)** PAE of ranked predictions, ranked 1 was selected and further used. **(b)** Coverage map and **(c)** pLDDT per position of ranked 1 model.

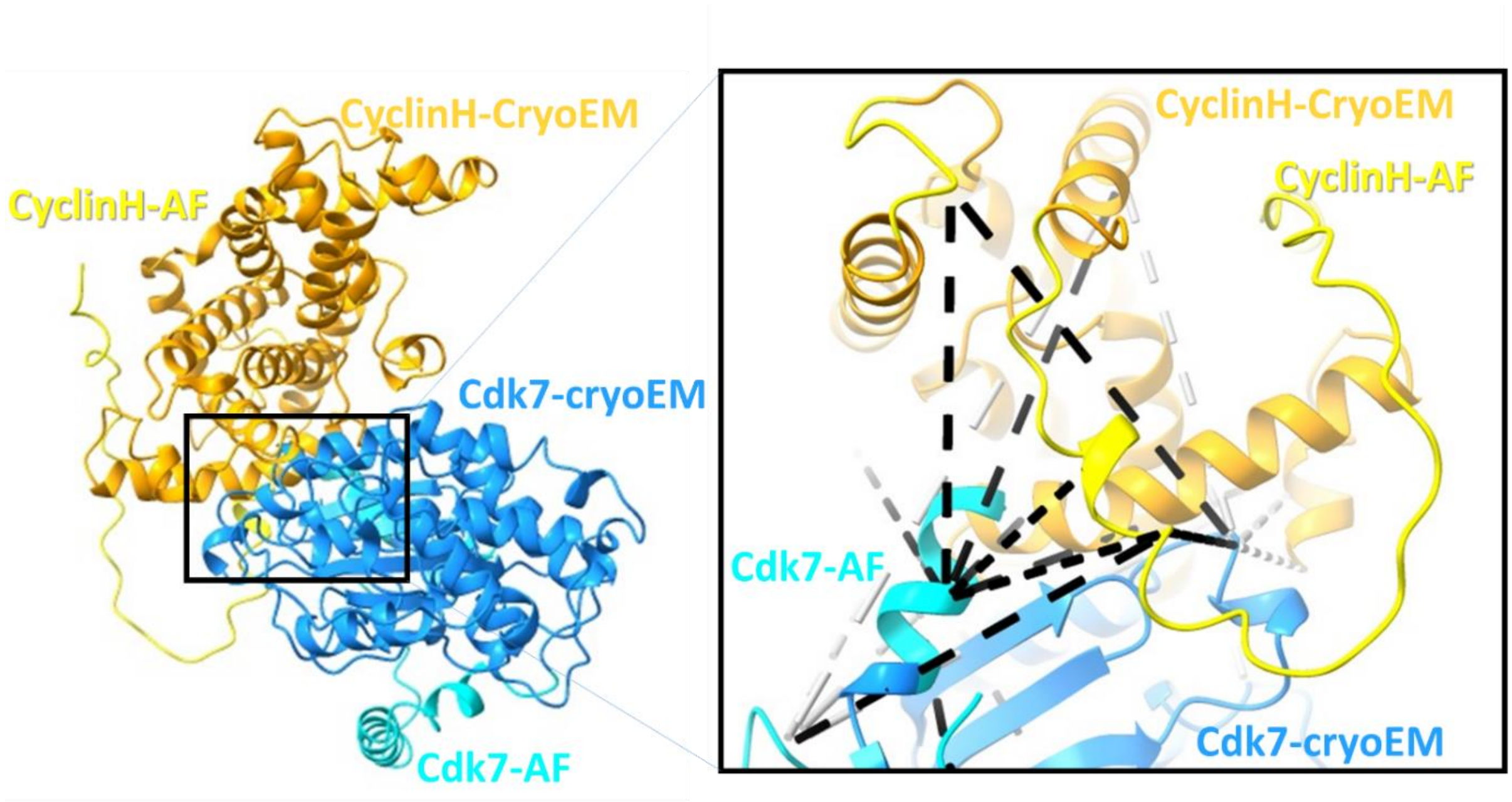

Supplementary Figure 7. Mapping of XLs of the CAK dataset on the structure of the model of the Cdk7/cyclin H binary complex (insert) obtained by superimposing the AF models of Cdk7 and Cyclin H on the cryo-101 EM structure of CAK (PDB: 8P6Y). Residues modelled from the cryo-EM map are shown in blue (CdK7) and gold (cyclin H) while those that could not be located in the experimental map and are modelled from AF prediction are reprinted in cyan (Cdk7) and yellow (cyclin H). XLs between CAs of two residues are represented by grey (in the case of 2 amino acids modeled from experimental data) or back (when 1 or the 2 amino acids modelled from AF predictions).

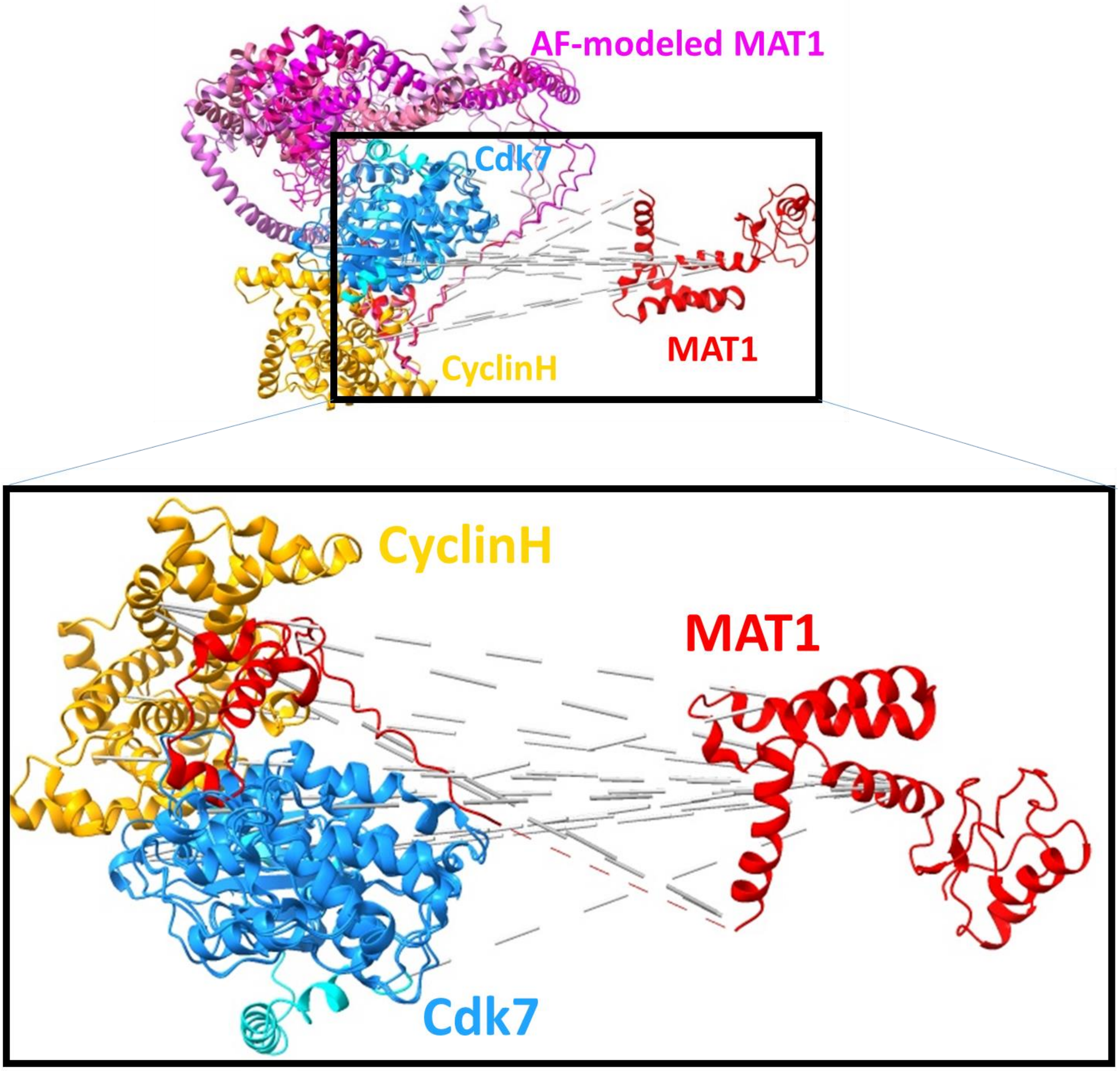

Supplementary Figure 8. Mapping of XLs of the CAK dataset on structure of CAK extracted from that human mediator–RNA polymerase II pre-initiation complex (PDB: 7NRV). Cdk7 is shown in blue, cyclin H in gold and MAT1 in red. XLs between are represented as dashed lines drawn between the Cα's of the two residues. In the insert the ribbon models MAT1 (in magenta) obtained of from 5 AF predictions of CAK MAT1 are superimposed.

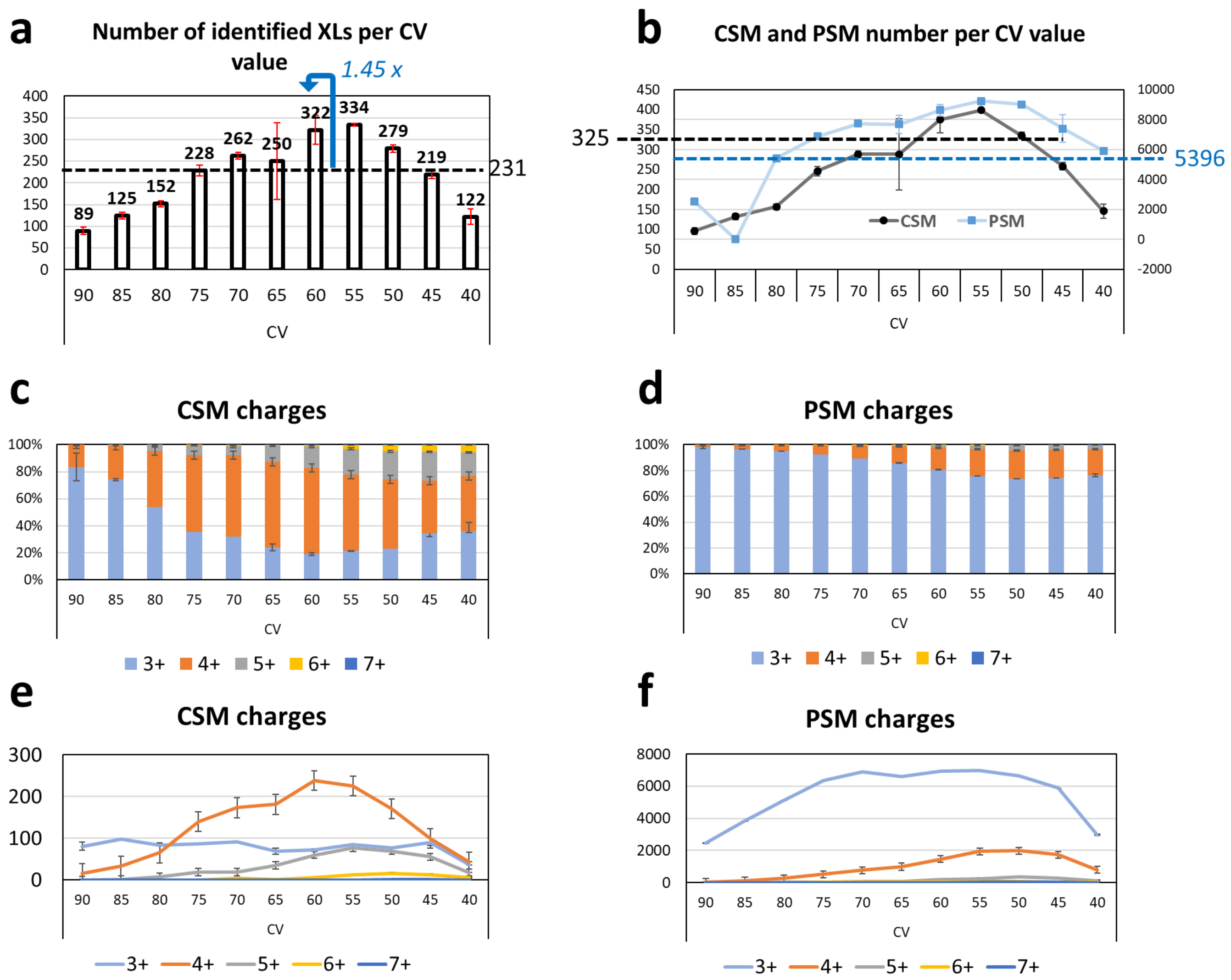

Supplementary Figure 9. Details of CV screening on XL-Hela lysate, using injection replicates (n=3). (a) Number ofunique XLs identified per CV value. All CSMs' SD are below 5 % apart from -65 V. (**b**) Number of CSM and PSM number per CV value identified with values obtained without FAIMS represented in dashed lines. (**c**) Detail of XLs charges states filtered, showed as proportion of CSM precursor charges for each CV value, (**d**) Detail of peptides charges states filtered, showed as proportion of PSM precursor charges for each CV value. (**e**) Detail of XLs charges states filtered, showed as absolute numbers of CSM precursor charges for each CV value. (**e**) Detail of peptides charges states filtered, showed as absolute numbers of PSM precursor charges for each CV value

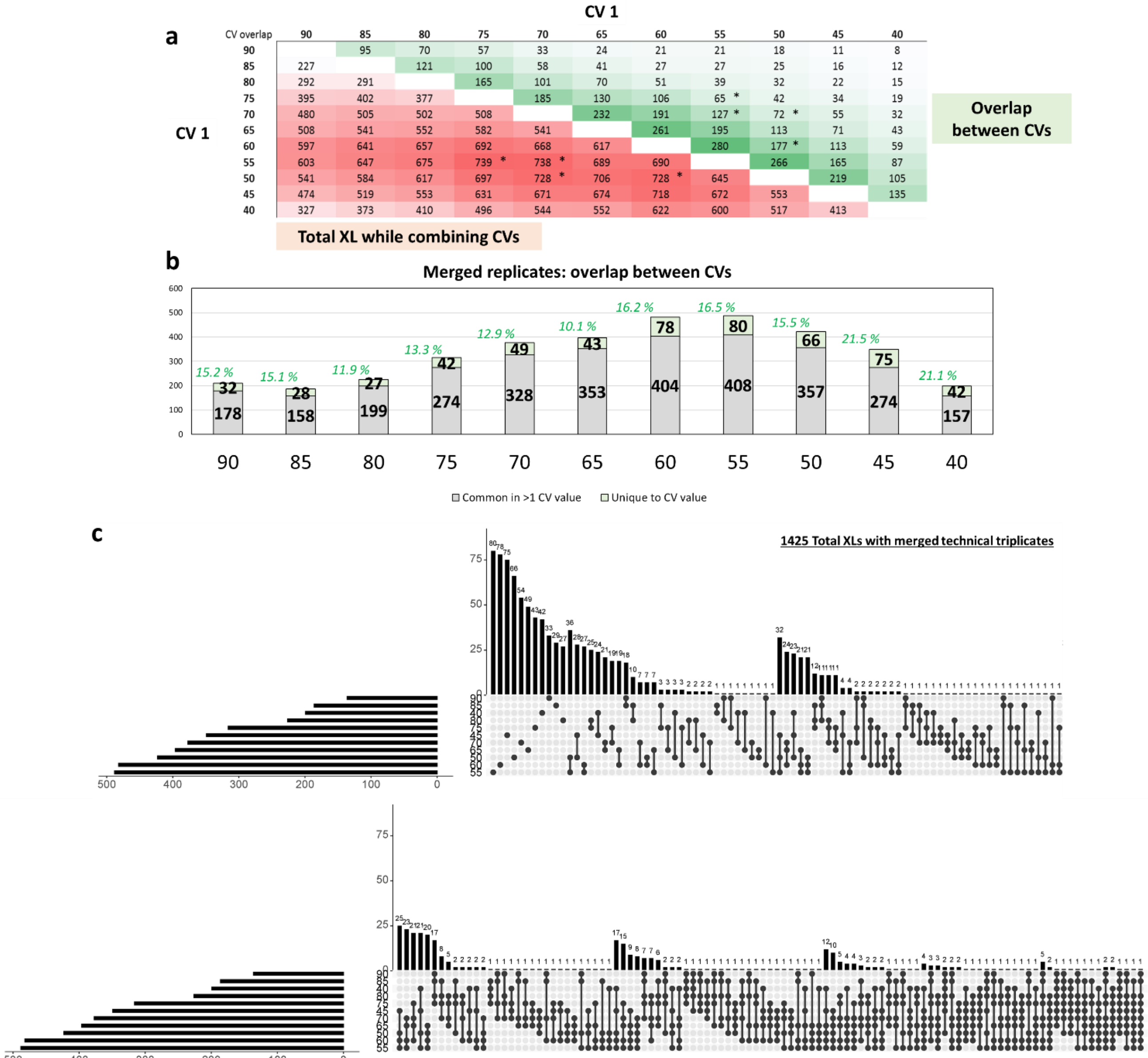

Supplementary Figure 10. Choice of best CV associations using merged technical triplicates. (a) Matrix showing associations of CV pairs with in red number XLs identified when combining the 2 CV and in green number of XLs found in common in both CVs. Best combinations of CV pairs (maximizing red values and minimizing green values) are highlighted with stars. **(b)** Number of XLs identified per CV divided in XLs uniquely found to in the CV value as well as XLs present in at least CV value, from merged technical replicates (n=3)**. (c)** upsetR plot showing the overlap between all CV values**.**

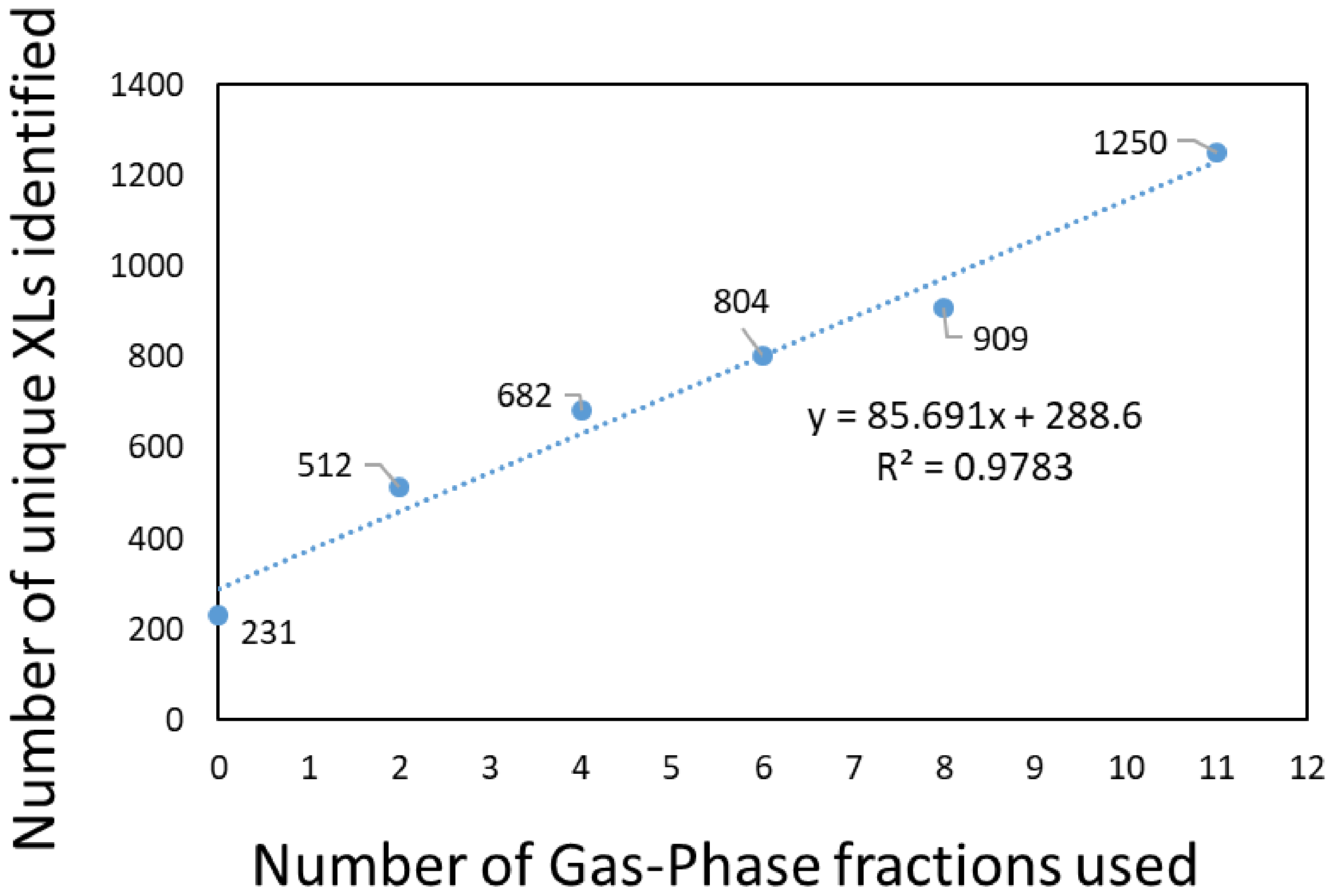

Supplementary Figure 11. Relation (linear fitting) between number of gas-phase fractions used and number of unique XLs identified for XL-HeLa lysate.

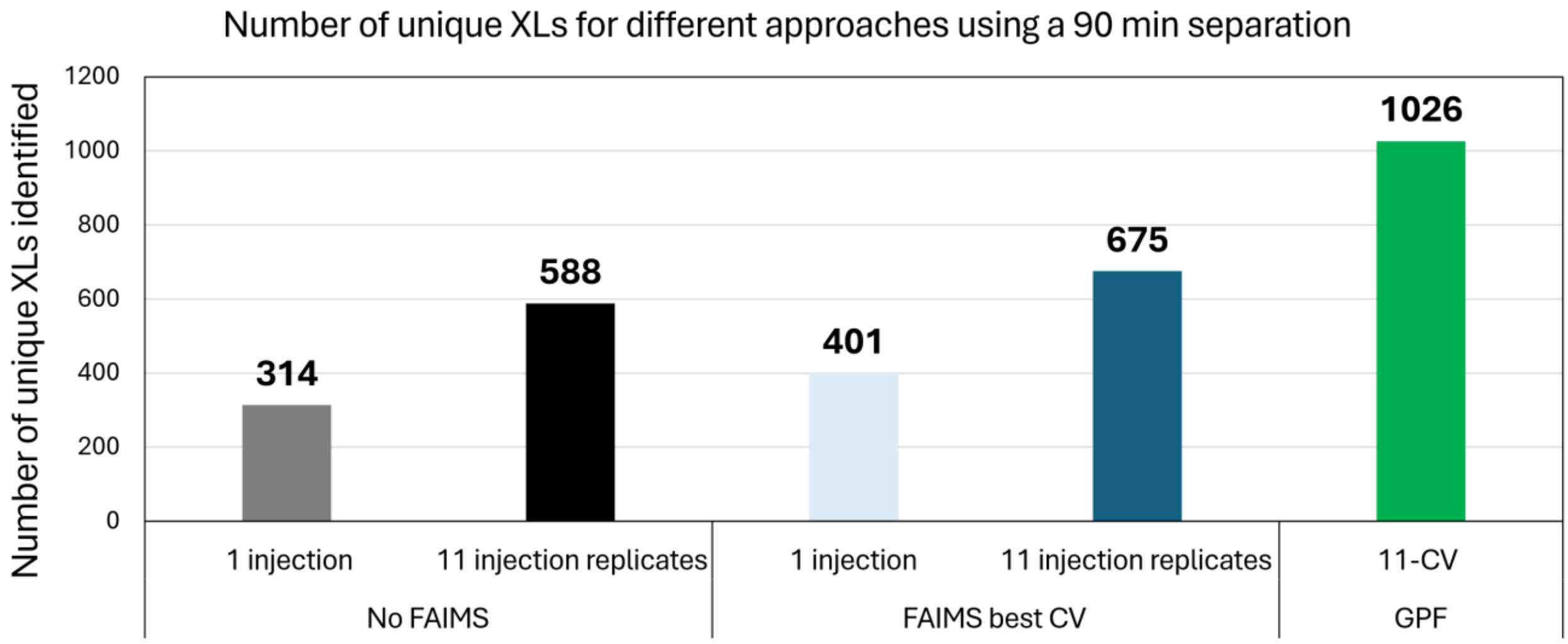

Supplementary Figure 12. Comparison of FAIMS-GPF with injection replicates

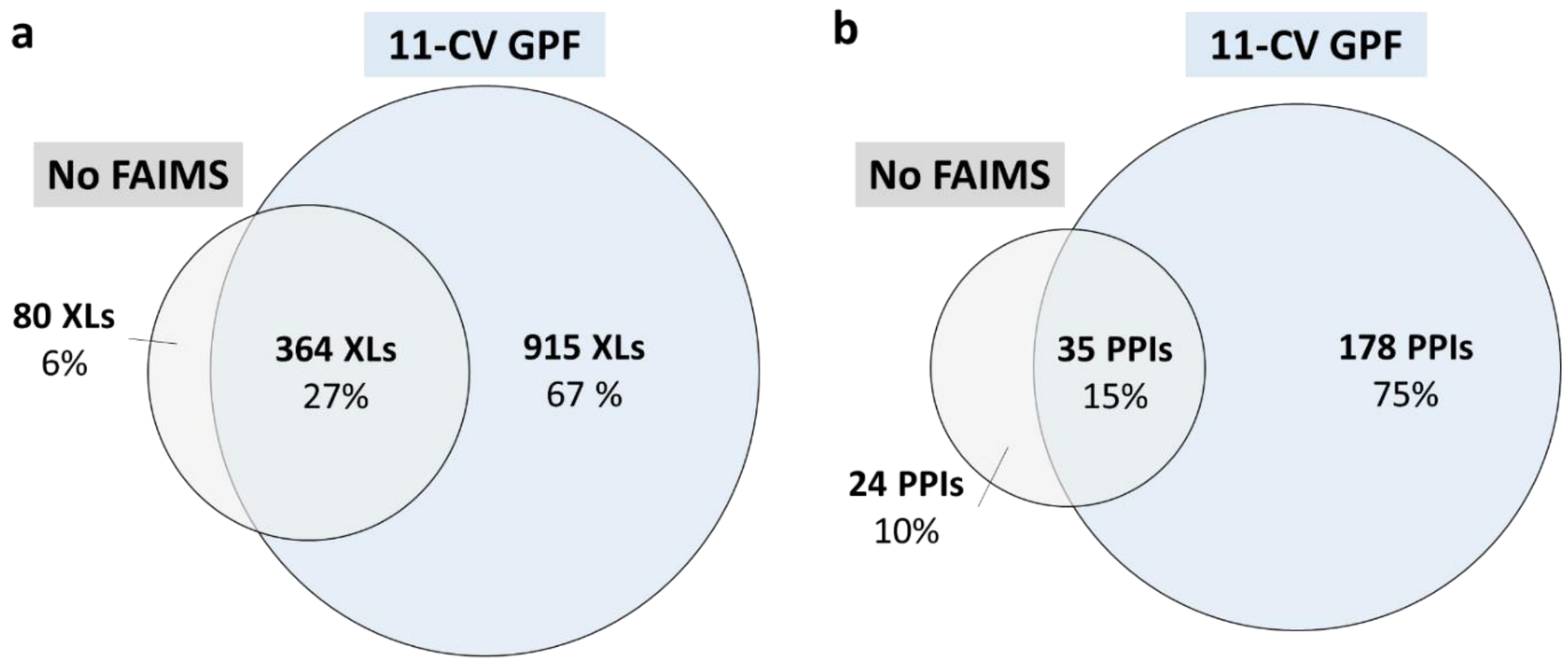

Supplementary Figure 13. Overlap between (a) XLs identifications and **(b)** PPIs between FAIMS-free and 11-CV GPF experiments.

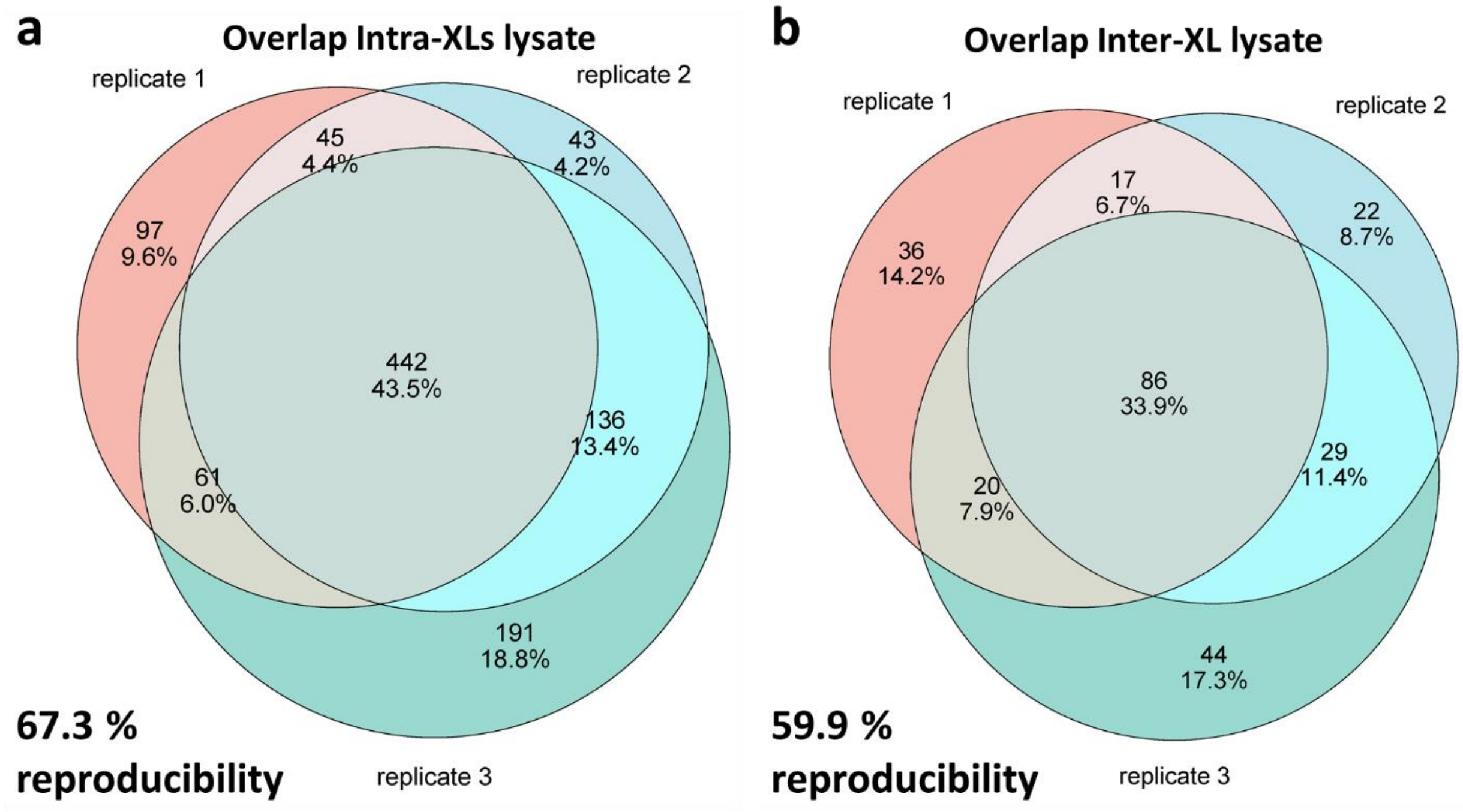

Supplementary Figure 14. Reproducibility of independent XL replicates of FAIMS-11-CV experiments at a proteome wide level for (a) intra- and **(b)** inter-XLs. Overlap between FAIMS-free and 11-CV GPF experiments.

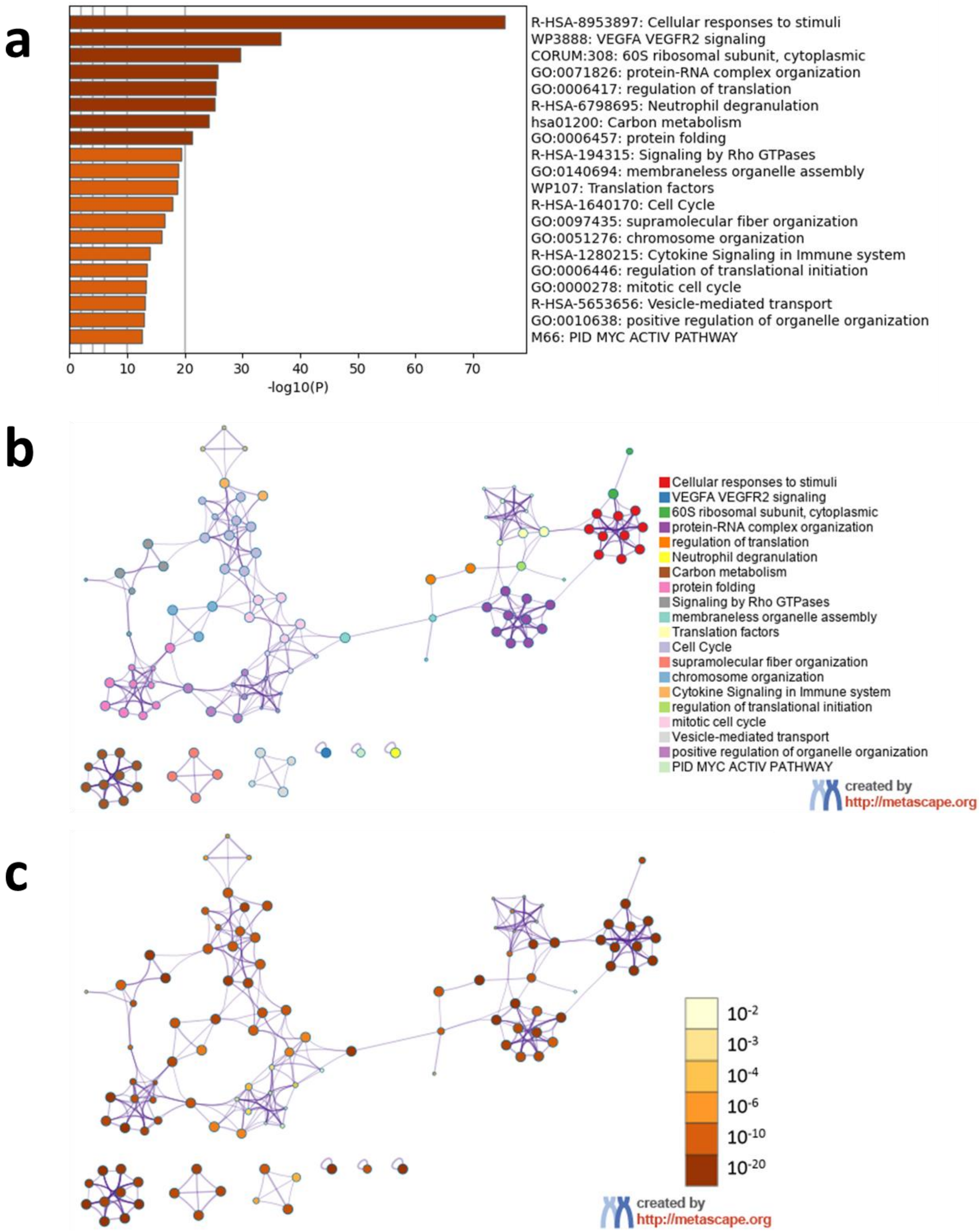

Supplementary Figure 15. Metascape[86] Gene Ontology enrichment analysis of cross-linked proteins only identified using FAIMS-GPF. (a) Bar graph of enriched terms across input gene lists, colored by p-values**.** Network of enriched terms: **(b)** colored by cluster ID, where nodes that share the same cluster ID are typically close to each other; **(c)** colored by p-value, where terms containing more genes tend to have a more significant p-value**.**

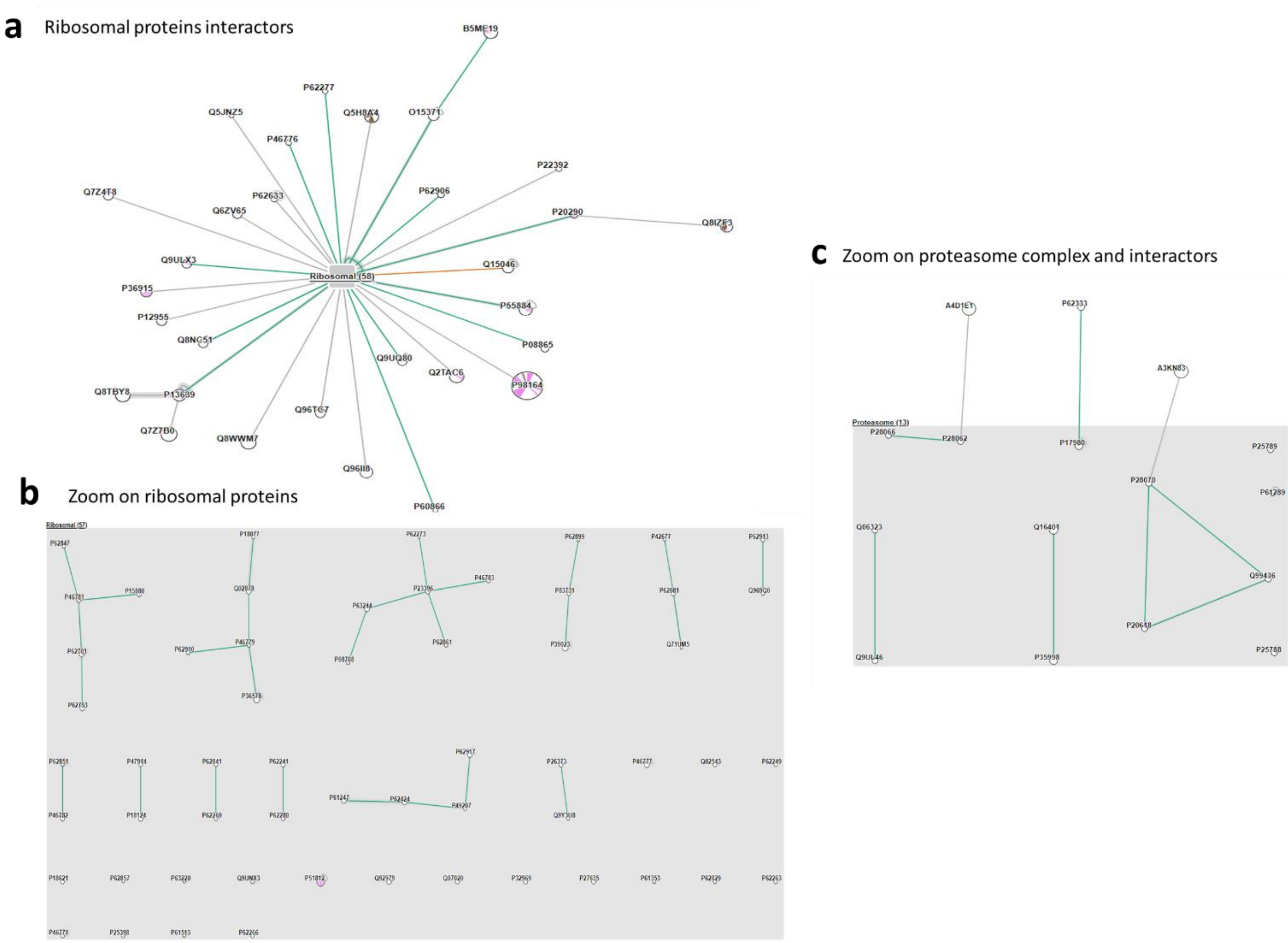

Supplementary Figure 16. (a) Zoom on ribosomal interactors identified**. (b)** Zoom on inter-ribosomal proteins interactions, **(c)** Zoom on proteasome proteins interactions.

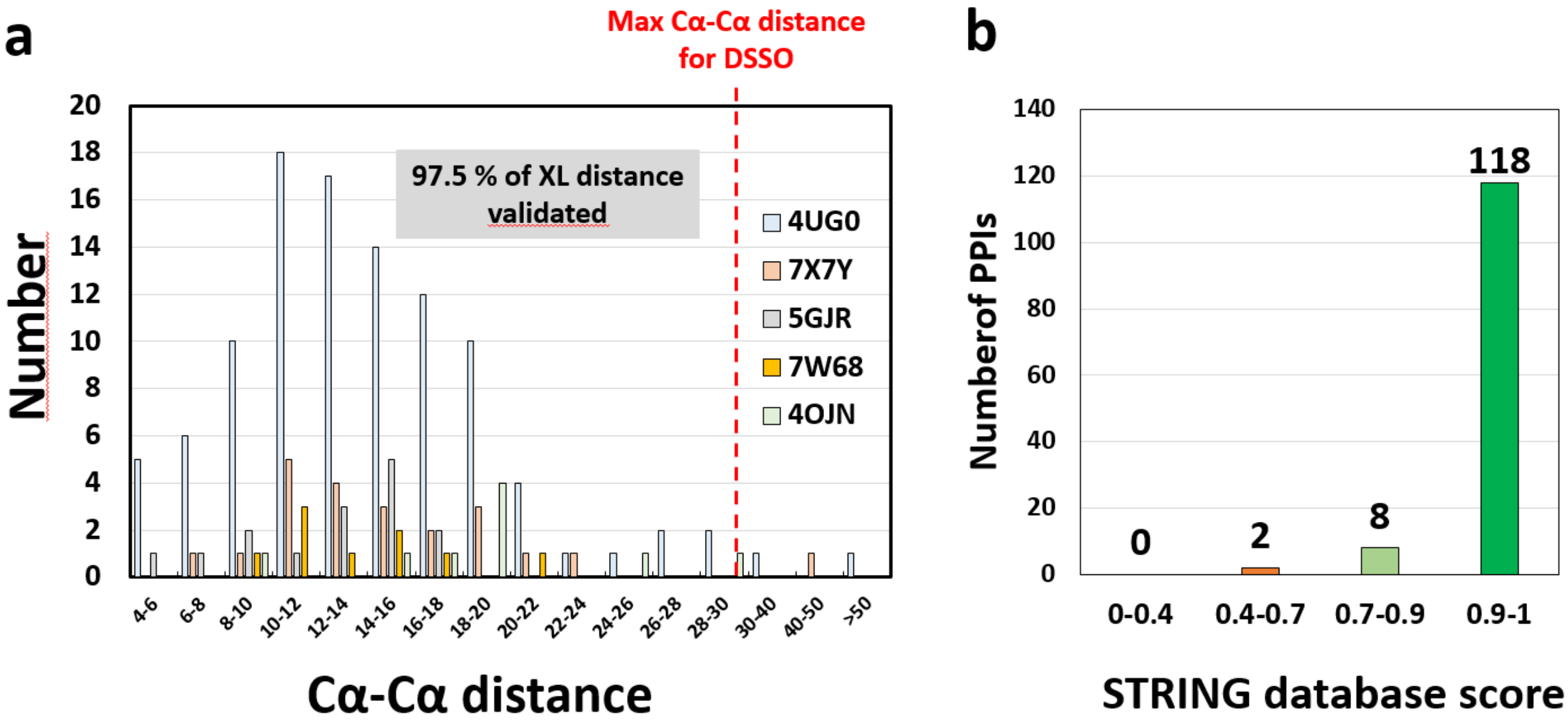

Supplementary Figure 17. Specificity of FAIMS 11-CV GPF dataset. (a) Ca-Ca distances measured after mapping of intra- and inter-XLs on 5 known structures exhibit a high percentage of validation (97.5 %), **(b)** Scores of PPIs identified present in STRING database.

a

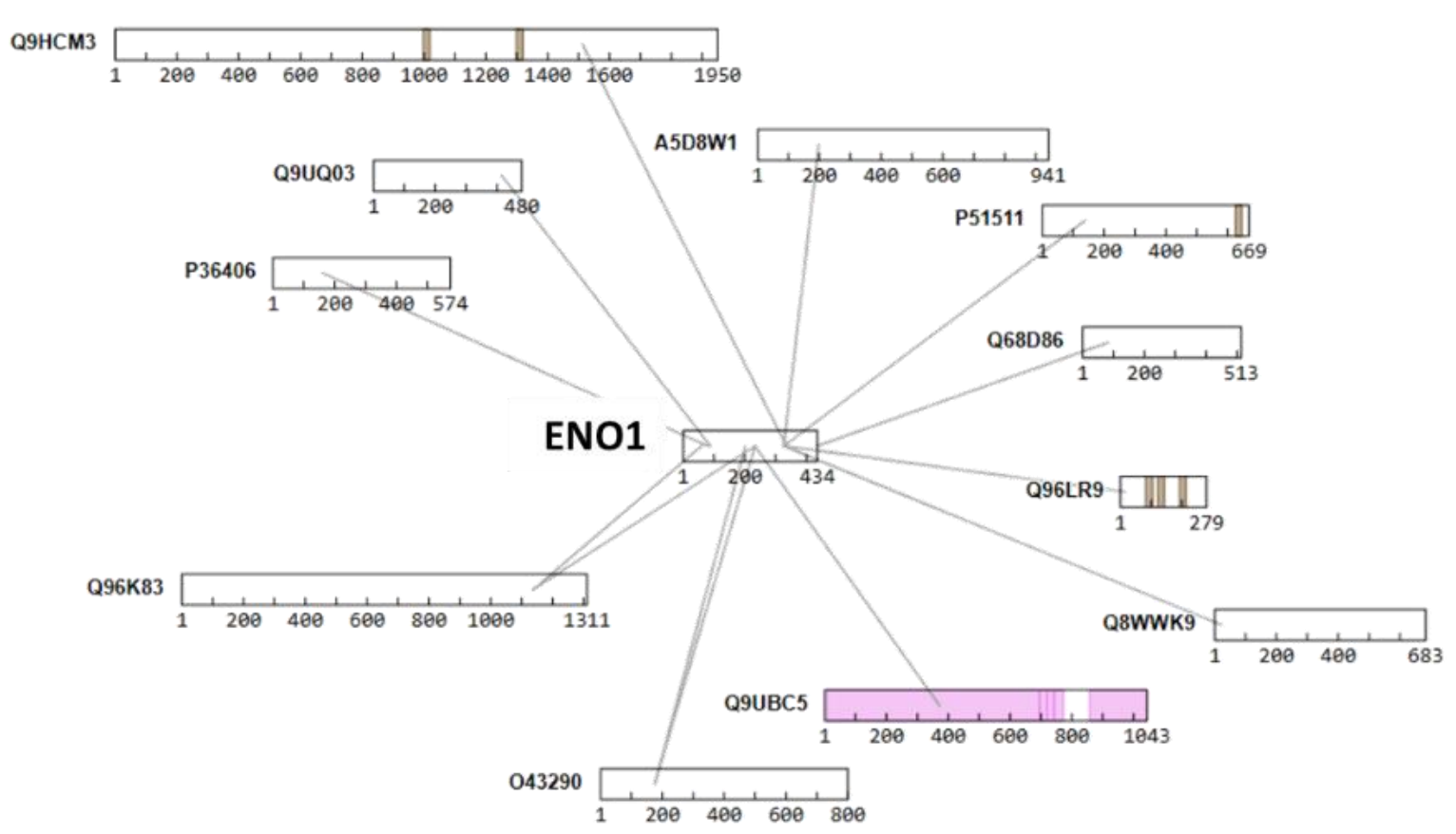

Protein 1	Protein 2	Potential biological Implications
ENO1	ZNF521	May suggest a role in nuclear functions or transcriptional regulation.
ENO1	CKAP2	May imply involvement in microtubule-associated functions and cell cycle regulation.
ENO1	Coronin-2B	Could reinforce ENO1's link to actin dynamics and cell motility.
ENO1	SNUT1	SNUT1 is a spliceosomal component. Interaction with ENO1 may suggest a nuclear role in RNA processing.
ENO1	MYO1A	This could hint at ENO1's involvement in membrane remodeling or vesicular transport.
ENO1	PLD1	May link ENO1 to signaling cascades or membrane-associated functions.
ENO1	KIAA1549	Could represent an unexplored function or tissue-specific interaction.
ENO1	C10orf2B	Might indicate a novel or tissue-specific function.

b

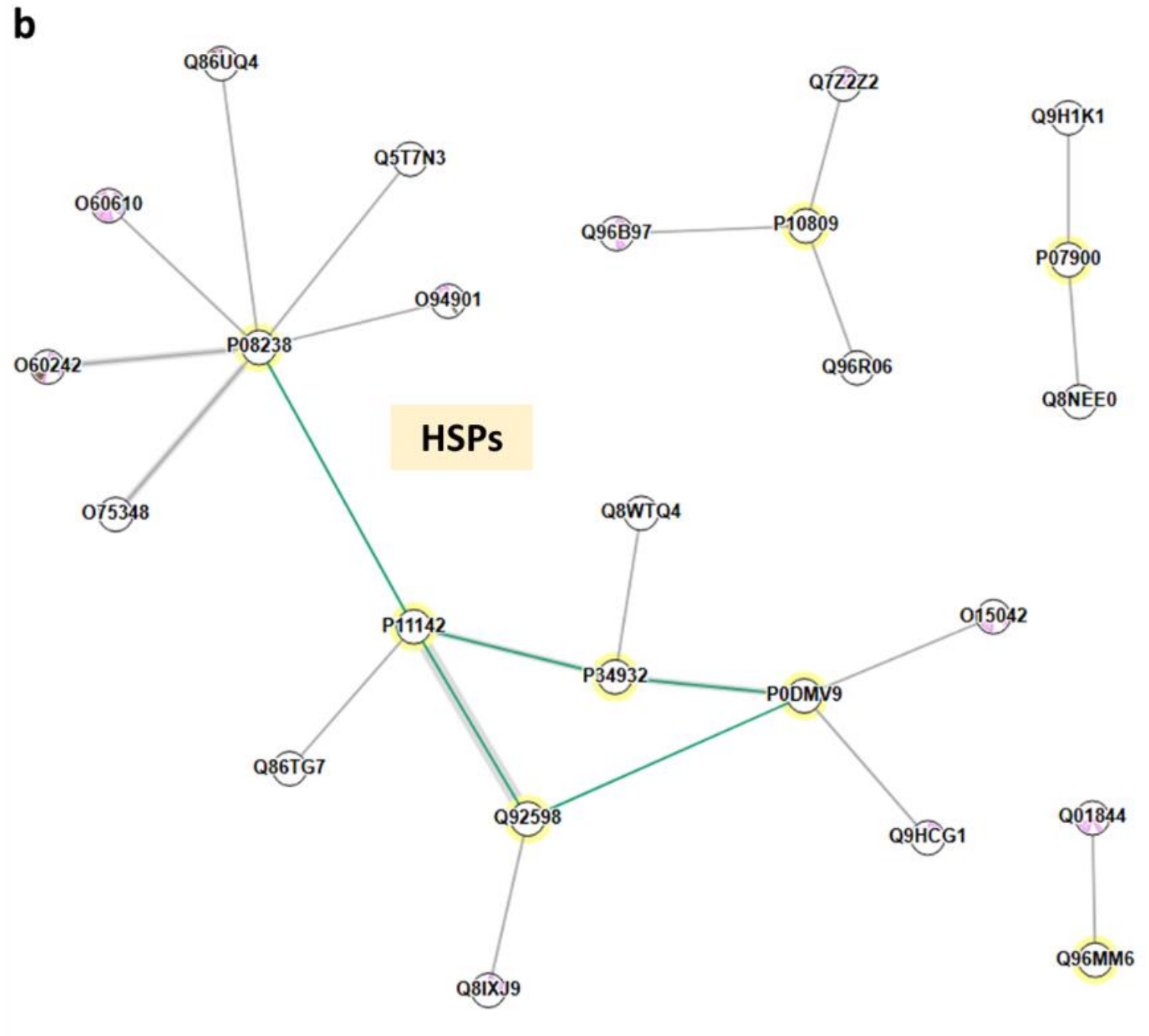

Protein 1	Protein 2	Potential biological Implications
HSP90AB1	DNAJB1	Suggests crosstalk between Hsp40 co-chaperones and Hsp90 chaperone systems.
HSPA1B	U2SURP	May suggest regulation of RNA splicing via Hsp70 interactions.
HSP90AB1	DNAJC7	DNAJC7 links Hsp70 and Hsp90 systems; suggests multi-chaperone assembly.
HSP90AB1	DIAPH1	Suggests role of Hsp90 in cytoskeletal regulation.
HSP90AA1	DNAJC3	May link Hsp90 to ER stress pathways.
HSPB1	DNAJB1	Possible collaboration between small HSP and Hsp40 in stress response.
HSPA1B	HSPA4	May represent functional interaction between Hsp70 isoforms.
HSPA4	DNAJC10	May suggest ER-based folding roles.
HSP90AB1	DNAJC3	ER-related co-chaperone possibly linking to Hsp90 pathway.
HSP90AB1	DNAJC13	May implicate Hsp90 in vesicle trafficking processes.

Supplementary Figure 18. Interaction networks of (a) ENO1 and (b) Heat-shock proteins. Interactions reported in STRING database are represented in green, known protein domains in Uniprot are highlighted in pink, transmembrane regions are highlighted in brown. Tables describe unreported interactions and their potential biological implications.

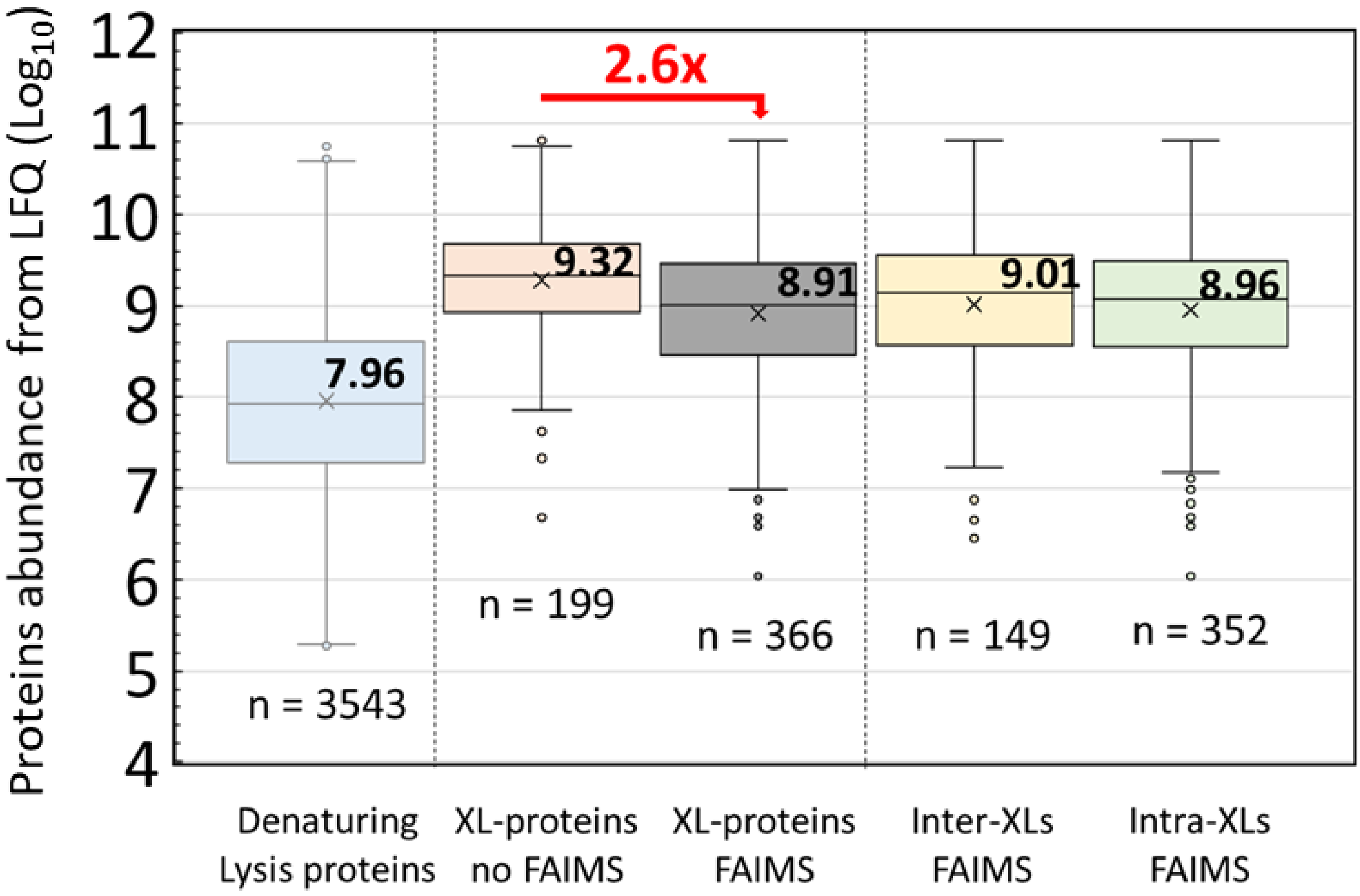

Supplementary Figure 19. Box-plot compares the LFQ protein abundance in whole cell lysate and abundances ofcross-linked proteins. Compared to the analysis without FAIMS, 11-CV GPF allows to reach proteins of 2.6 times less abundant. No major difference was found between abundance of protein with intra and inter-XLs. Values come from merged biological replicates and n displays number of values that could be plotted per category.

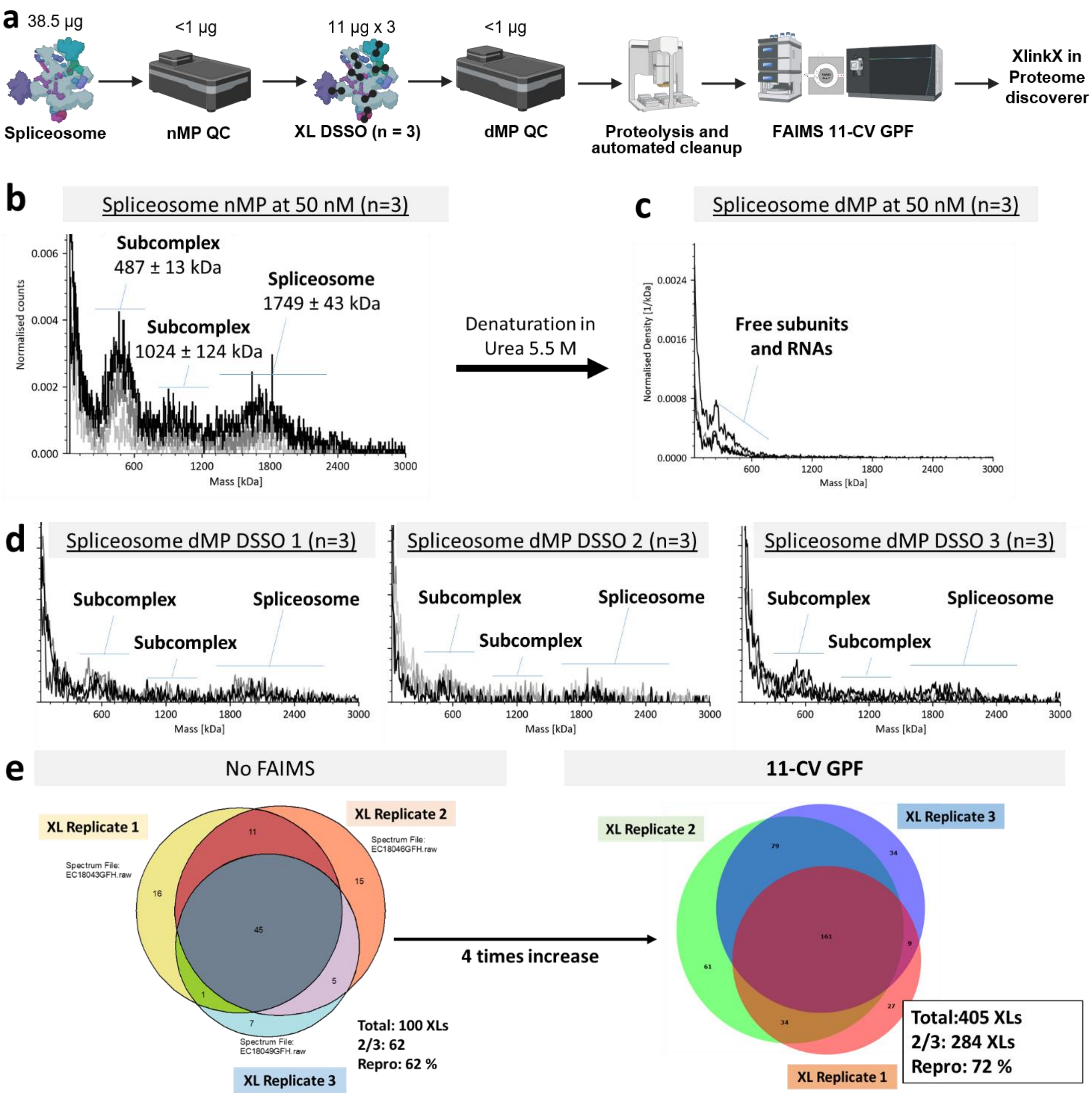

Supplementary Figure 20. Description and validation of spliceosome XL-MS dataset. (a) Worfkflow used for the FAIMS-based XL-MS triplicate experiments on spliceosome. Created in BioRender. Hoernel, C. (2026) https://BioRender.com/t6nnuji (b) nMP analysis of intact spliceosome highlights the co-existence of two subcomplexes. **(c)** dMP control of non-XL spliceosome shows the complete denaturation into RNA and protein subunits. **(d)** dMP control of XL reaction replicates highlights the presence of XL-stabilizedsub-complexes and whol spliceosome in each replicates. **(e)** On the left, venn diagram shows theoverlap between unique XLs identified in XL replicates analyzed without FAIMS. On the right, venn diagram shows the overlap between unique XLs identified in XL replicates analyzed using 11-CV FAIMS GPF. Reproducibility is increased by 10 % when using FAIMS-GPF and number of all XLs as well as reproducible XLs is multiplied by >4.

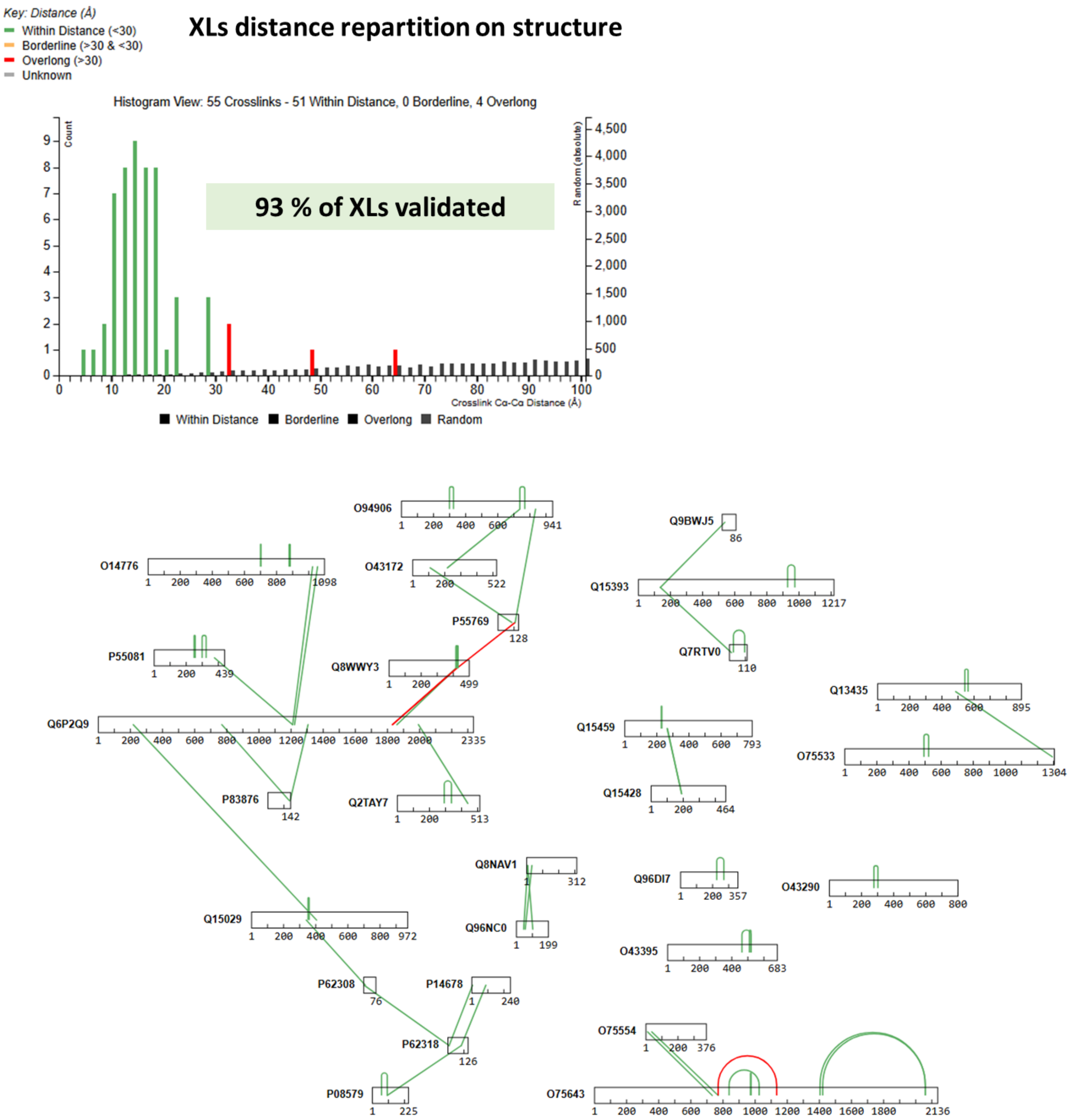

Supplementary Figure 21. Details of XLs mapping on B-complex structure. On the top, XLs distance repartition. On the bottom, network of XLs that could be mapped on 8QO9 PDB structure with violated distances indicated in red. This highlights the ability of FAIMS-GPF to retrieve XLs of high structural relevance and specificity.

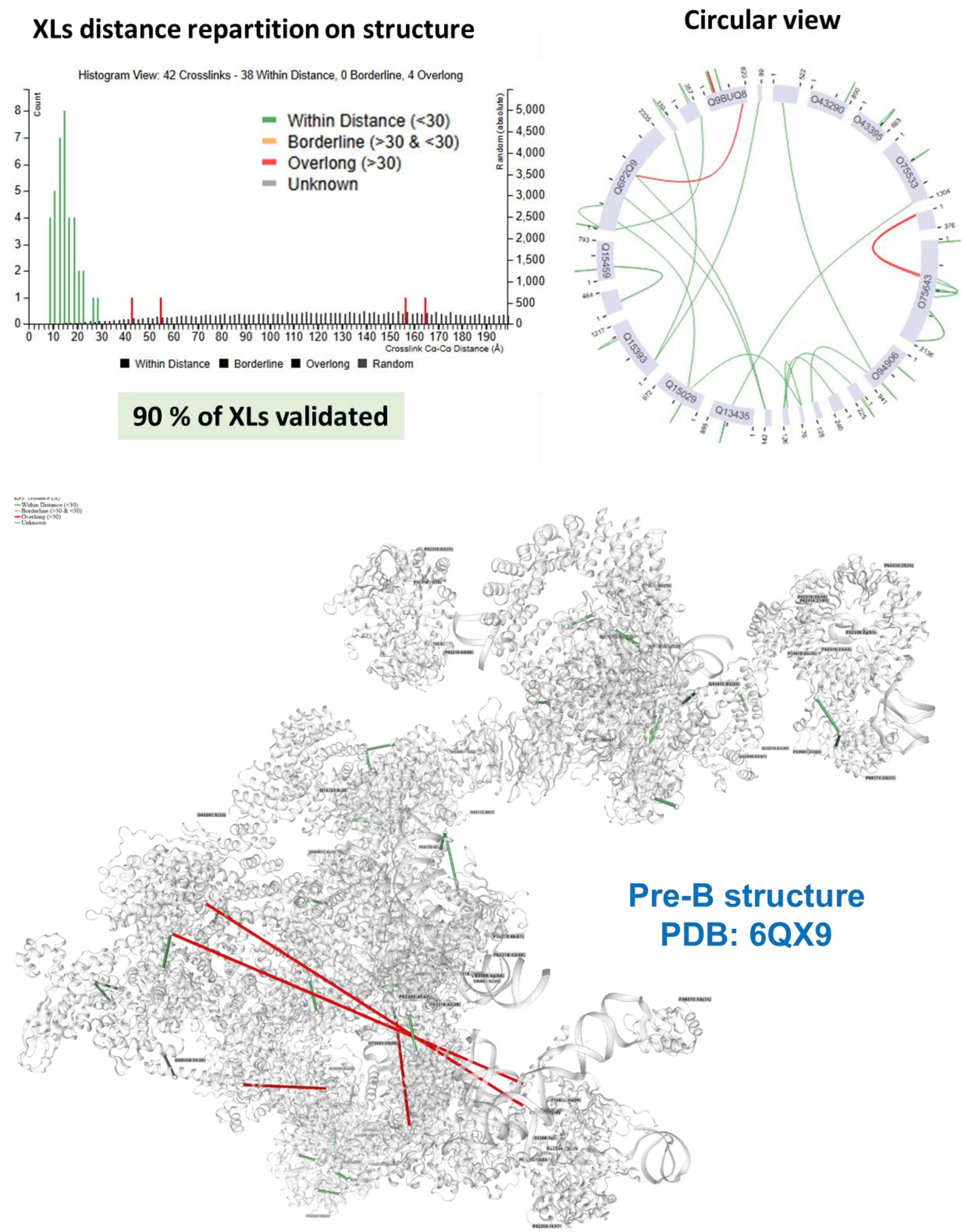

Supplementary Figure 22. Distance-validation of reproducible XLs on different spliceosome structures potentially co-existing in the sample. Among the 42 XLs mapped in the human pre-B-act-1 structure (PDB: 7ABG), 90% could be distance validated. Mapping XLs on human pre-B (PDB: 6QX9) structure yielded to higher rate of distance violated identification consistent with the expected nature of the analyzed sample.

Site 1	Protein 1	Site 2	Protein 2
7	Cdk7	52	Cdk7
6	Cdk7	14	Cdk7
14	Cdk7	32	Cdk7
6	Cdk7	32	Cdk7
32	Cdk7	52	Cdk7
32	Cdk7	328	Cdk7
139	Cdk7	160	Cdk7
139	Cdk7	328	Cdk7
52	Cdk7	164	Cdk7
6	Cdk7	328	Cdk7
52	Cdk7	328	Cdk7
328	Cdk7	342	Cdk7
14	Cdk7	328	Cdk7
6	Cdk7	342	Cdk7
52	Cdk7	274	CyclinH
164	Cdk7	78	CyclinH
328	Cdk7	8	CyclinH
32	Cdk7	189	CyclinH
342	Cdk7	8	CyclinH
6	Cdk7	8	CyclinH
52	Cdk7	8	CyclinH
6	Cdk7	33	CyclinH
6	Cdk7	39	CyclinH
6	Cdk7	72	CyclinH
52	Cdk7	72	CyclinH
52	Cdk7	121	CyclinH
6	Cdk7	189	CyclinH
52	Cdk7	189	CyclinH
6	Cdk7	292	CyclinH
6	Cdk7	295	CyclinH
32	Cdk7	295	CyclinH
52	Cdk7	295	CyclinH
161	Cdk7	78	CyclinH
164	Cdk7	189	CyclinH
32	Cdk7	72	CyclinH
8	CyclinH	239	CyclinH
274	CyclinH	295	CyclinH
8	CyclinH	189	CyclinH
39	CyclinH	295	CyclinH
8	CyclinH	72	CyclinH
72	CyclinH	189	CyclinH
189	CyclinH	274	CyclinH
8	CyclinH	14	CyclinH
32	Cdk7	70	Mat1
8	CyclinH	70	Mat1
72	CyclinH	70	Mat1
189	CyclinH	70	Mat1
274	CyclinH	70	Mat1

Supplementary Table 1. XLs identified (FT 2/3) after XL-MS experiments on CAK without FAIMS.

Site 1	Protein 1	Site 2	Protein 2
6	Cdk7	32	Cdk7
6	Cdk7	41	Cdk7
6	Cdk7	52	Cdk7
6	Cdk7	291	Cdk7
6	Cdk7	342	Cdk7
7	Cdk7	52	Cdk7
14	Cdk7	32	Cdk7
14	Cdk7	328	Cdk7
28	Cdk7	32	Cdk7
32	Cdk7	52	Cdk7
32	Cdk7	328	Cdk7
32	Cdk7	342	Cdk7
41	Cdk7	52	Cdk7
52	Cdk7	160	Cdk7
52	Cdk7	164	Cdk7
52	Cdk7	170	Cdk7
52	Cdk7	291	Cdk7
52	Cdk7	328	Cdk7
52	Cdk7	342	Cdk7
139	Cdk7	328	Cdk7
160	Cdk7	328	Cdk7
160	Cdk7	342	Cdk7
328	Cdk7	342	Cdk7
6	Cdk7	8	CyclinH
6	Cdk7	14	CyclinH
6	Cdk7	39	CyclinH
6	Cdk7	72	CyclinH
6	Cdk7	189	CyclinH
6	Cdk7	274	CyclinH
6	Cdk7	292	CyclinH
6	Cdk7	295	CyclinH
32	Cdk7	8	CyclinH
32	Cdk7	72	CyclinH
32	Cdk7	189	CyclinH
32	Cdk7	274	CyclinH
32	Cdk7	295	CyclinH
52	Cdk7	39	CyclinH
52	Cdk7	72	CyclinH
52	Cdk7	121	CyclinH
52	Cdk7	189	CyclinH
52	Cdk7	274	CyclinH
52	Cdk7	295	CyclinH
160	Cdk7	72	CyclinH
164	Cdk7	189	CyclinH
291	Cdk7	189	CyclinH
328	Cdk7	8	CyclinH
342	Cdk7	8	CyclinH

Site 1	Protein 1	Site 2	Protein 2
6	Cdk7	70	Mat1
6	Cdk7	80	Mat1
6	Cdk7	135	Mat1
32	Cdk7	70	Mat1
52	Cdk7	70	Mat1
52	Cdk7	127	Mat1
160	Cdk7	70	Mat1
291	Cdk7	70	Mat1
328	Cdk7	70	Mat1
342	Cdk7	70	Mat1
8	CyclinH	12	CyclinH
8	CyclinH	14	CyclinH
8	CyclinH	189	CyclinH
8	CyclinH	239	CyclinH
8	CyclinH	253	CyclinH
33	CyclinH	295	CyclinH
39	CyclinH	189	CyclinH
39	CyclinH	292	CyclinH
39	CyclinH	295	CyclinH
72	CyclinH	189	CyclinH
72	CyclinH	295	CyclinH
189	CyclinH	274	CyclinH
189	CyclinH	295	CyclinH
190	CyclinH	295	CyclinH
274	CyclinH	295	CyclinH
8	CyclinH	70	Mat1
8	CyclinH	167	Mat1
72	CyclinH	70	Mat1
189	CyclinH	70	Mat1
189	CyclinH	127	Mat1
189	CyclinH	167	Mat1
274	CyclinH	70	Mat1
70	Mat1	80	Mat1
70	Mat1	176	Mat1
127	Mat1	135	Mat1

Supplementary Table 2. XLs identified (FT 2/3) after XL-MS experiments on CAK with 4-CV GPF FAIMS method.
