# Supplementary material for: FAIMS-GPF XL-MS: crosslinking-mass spectrometry based on gas-phase fractionation
Source: Nat Commun. 2026 Jul 23;17:8985. doi: 10.1038/s41467-026-75736-9 (PMC13507205; doi:10.1038/s41467-026-75736-9)
Supplement: Supplementary file 3 [file 41467_2026_75736_MOESM3_ESM.pdf]

nature portfolio

Peer Review File

FAIMS-GPF XL-MS: crosslinking-mass spectrometry based on gas-phase fractionation

Corresponding Author: Dr Sarah Cianférani

Parts of this Peer Review File have been redacted as indicated to remove third-party material.

This file contains all reviewer reports in order by version, followed by all author rebuttals in order by version.

Attachments originally included by the reviewers as part of their assessment can be found at the end of this file.

Version 0:

Reviewer comments:

Reviewer #1

(Remarks to the Author)
Reviewer comments:
The authors of this publication introduce a crosslinking-MS pipeline that integrates FAIMS-based gas-phase fractionation (GPF) to enhance detection sensitivity of crosslinked peptides. The workflow reportedly offers superior coverage of protein-protein interactions (PPIs), reduces required sample quantities, and cuts acquisition time. Case studies include CAK (purified), HeLa lysate, and the spliceosome, benchmarking the 11-CV FAIMS-GPF protocol against unfractionated setups.

Major concerns:
Can the authors include a control experiment injecting a sample 11 times and compare the difference between those numbers with their 11CV-GPF workflow? It is known that DDA data produces more data with each injection of a technical replicate and with each injection the numbers of identified features will increase. This is due to the "random sampling" of DDA experiments. This is also true for crosslinking experiments. By injecting a sample two times and injecting the sample 10 times the identification rate increases by approximately 50%. It would be important to show that gain for CV fractionation really results from the CVs instead of injecting a sample 11 times. This can be easily tested by selecting the best working CV and injecting the sample 11 times and see how much each technical replicate increases the total number of crosslinks. If the gain of identification for both methods is the same, then this experimental set up does not give new insides.
"In comparison, similar performance using existing 284 methods would require extensive in-solution fractionations (> 20 fractions) using high amounts of starting 285 material (> 100 µg per sample, usually around 500 µg)27,28,62–64." This statement is not true, since peptide fractionation can be also carried out with 50ug of stating material. I would also argue that 3 days of measurement time for one sample is a lot of time and should be not over exaggerated as benefit here.
In the following publication the authors needed 1 day per fractionation experiment, which resulted in over 4000 unique links and about 300 PPIs (https://doi.org/10.1101/2025.02.25.640081). It would be good to rephrase the paragraph and but the current developed workflow into perspective.
I would like to point to another publication showing high PPIs for very low starting material amounts, which also shows higher numbers as reported here:
https://onlinelibrary.wiley.com/doi/epdf/10.1002/anie.202501845 (1426 crosslink sites per 1e6 SY5Y cells
).
I am missing the reasoning for the newly 11CV-GPF developed method, it seems that the literature already outperforms the method here in terms of numbers and low starting material. Could the authors clarify or refine the benefit of their method?

- Numerous PPIs reported as novel—particularly those involving ENO1 and heat shock proteins—are not functionally validated by any orthogonal method. The claim that these represent biologically meaningful discoveries remains speculative in the absence of biochemical, genetic, or imaging-based evidence. This limits the strength of the manuscript's biological conclusions, especially for readers interested in translational relevance or mechanistic follow-up.
- A significant fraction of the crosslinks mapped onto structures (e.g., 26% in the CAK cryo-EM model) violate the commonly accepted 30 Å distance constraint for DSSO. The causes of these violations are not systematically examined—e.g., missing structure, domain flexibility, or model inaccuracy. Moreover, several mapped crosslinks rely on AlphaFold-predicted

structures, but no confidence metrics or uncertainty considerations are provided for these models.
- why different settings were used between the analysis? Sometimes linkage sites are set on K,S,T,Y sometimes only on K? This inconsistency makes comparisons between datasets problematic and raises concerns about methodological uniformity. The decision to use broader linkage rules in one case is not explained or controlled for.
- if you report unique crosslinks the FDR should be also calculated on this level. CSM level FDR of 1% is not enough and produces most likely false identification on PPI level. It needs to be proven whether the novel links identified in this manuscript hold true if the FDR is calculated on PPI or at least on residue pair level. See have a look at the following publication:
DOI: 10.1038/s41467-021-23666-z
-t he manuscript highlights multiple novel interactions that are not present in STRING or other databases, but provides limited interpretation of their functional significance. These interactions are not placed within known pathways, cellular compartments, or network modules. Moreover, there is no analysis of whether these links reflect novel biology or potential false positives due to incomplete reference data.

Minor issues:
• Abbreviations such as “XL,” “GPF,” and “CV” are introduced without definition or used inconsistently between text and figures.
• Several figure panels (e.g., Figure 3b, Figure 5a) have small fonts, complex color schemes, or insufficient axis labeling, which reduces interpretability.
• Network and structure mapping figures use inconsistent or ambiguous color codes without clear legends or explanations in captions.
• The use of a 30 Å cutoff for Cα–Cα validation is not defended with theoretical or empirical rationale. Please cite at least a paper where the threshold was used before or better calculate the real distance cut of by using the length of the amino acid side chains (here lysine, STY depending on what the data is based on) and the crosslinker spacer length. A bit flexibility towards distance cut offs is possible but usually the crosslink distances are chosen to be overlength, which is an old artifact from the beginning of the crosslinking aera.
• The Eclipse is a high mass accuracy instrument (when well calibrated), 10ppm MS1 and 20ppm MS2 seems to be too loose.
• What is the rational to set the charge states to +2 to +7 for a linear proteomics MS method?

Reviewer #2

(Remarks to the Author)
The study presented by Gizardin-Fredon et al outlines the utility of gas-phase fractionation for mass spec crosslinking analysis. Improving dynamic range/sensitivity of detection of crosslinked peptides/proteins is a hot topic currently and there is a need for new crosslinker chemistries as well as analytical methods. There are several publications which demonstrated the utility of gas fractionation using FAIMS device. Moreover, such a method using several FAIMS CVs in one run(internal CV stepping), is considered a standard method for XL-MS to improve dynamic range usually in combination with enrichment, in-solution offline fractionation. Authors suggested that single FAIMS CV fractionation- external CV stepping method provides alternative solution and enables the same depth of analysis as multiple CVs FAIMS method in combination with offline fractionation. Overall, this study highlights the use of FAIMS for crosslinking analysis and provides some novelty which can be beneficial for the community after addressing the following points:
Major concerns
What is exactly the role of Quality Control(QC) by Mass Photometry(MP) prior crosslinking? Why denatured MP was performed vs denatured intact MS analysis. Looks like native /denatured MS analysis will provide the same QC data and was actually used in some experiments.
Maunscript will benefit if authors provide a direct comparison of the two methods at least for CAK samples: 4 internal CV method vs 4 external CV method using the same CVs with similar gradient length of 4 hrs and similar sample load. Authors did some comparison as in supplementary figure 1, but in this case internal CV gradient was equal to 1 external CV method or twice shorter and reported difference was probably due to instrument speed.
Authors highlighted low amount of sample and short instrument time as additional benefits for new developed strategy and compared number of proteome level identified XL with obtained from SEC or SCX fractionation using higher amount of stating material as on page 13: At a proteome-wide level, the 11-CV FAIMS-GPF protocol using a total of 20 µg of starting material was able to provide 1269 XLs and 213 PPIs in less than 3 days, numbers that were previously only reached after extensive in-solution fractionation (e.g. using ≥ 20 strong cation exchange fractions for a total of ~500 µg of starting material and requiring weeks of sample prep and instrument time)27,28,62,63,81. Maybe this is accurate statement for the earlier results as in references 27& 28 but number of identified crosslinkers for example in reference 63 is significantly higher – it reported 4260 unique crosslinkers from 1357 proteins in 24 hrs of instrument time using 1 SEC fraction and 12 High pH fractions – please revise lines 371-373 accordingly to reflect current status of in-solution fractionation with/without FAIMS for proteome wide crosslinking analysis

Additionally, I have few minor comments:

This sentence on page 4 needs to be corrected “such as sulfoxide-based DSSO22 and DSBU23”, DSBU crosslinker is not sulfoxide based.
References 30 &36 are identical. Reference 30 is a preprint of reference 36 and needs to be replaced/merged by 36.
Page 9 line 235-236 – how many runs of unfractionated HeLa without FAIMS were performed 3 per biological replicate or just one
Page 9 line 240-241 – typo 2.8 times increase not time same for line 241- 3.6 times increase not time

Version 1:

Reviewer comments:

Reviewer #1

(Remarks to the Author)
I would like to express my sincere thanks to the authors for their detailed and extensive work in answering my questions as thoroughly as possible.
With the exception of question 11, I am very satisfied with the answers. Thank you again for your efforts.

Now I would like to explain my arguments for question 11 in more detail.
The software node used in Proteome Discoverer is called XlinkX and was originally developed by Fan Liu's group.

Several publications, including those by the Rappsilber group, have shown that the FDR of XlinkX is very high at nearly 40%. As a result, an improved version was developed, which, however, still has a very high FDR rate.

It is also a fact that the original developer of the software no longer uses their own software, which was published several years ago, but instead uses newly developed software at her lab called Scout. https://github.com/theliulab/Scout

In response to the authors' reply, I would also like to note that best practice in 2018 will no longer be best practice in 2026, as the field has also developed enormously.
Anal. Chem. 2021, 93, 11442–11450; Nat. Protoc. 2018, 13, 2964–2990
I would therefore ask you to search for the data using a second independent software node called MS Anika, which was also developed for Proteome Discoverer, and compare the results.
https://www.nature.com/articles/s42004-024-01386-x

Since MS-Anika is integrated as simple node into Proteome Discoverer, re-analysis should not be too difficult, and the additional data can answer my above question to my complete satisfaction.

Reviewer #2

(Remarks to the Author)
The authors provided detailed responses to my concerns, and overall I can see improvements in the clarity of the writing and the results. However, some of my concerns and typographical errors were not completely addressed or corrected.

Major concerns
1) What is exactly the role of Quality Control (QC) by Mass Photometry (MP) prior crosslinking? Why denatured MP was performed vs denatured intact MS analysis. Looks like native /denatured MS analysis will provide the same QC data and was actually used in some experiments.
Answer: We would like to thank the reviewer for this question. In this study we first use mass photometry in native conditions as an initial sample quality assessment. Thus, before cross-linking reaction, mass photometry is carried on intact spliceosome in its native buffer to assess its integrity. It is very useful as an alternative to native-MS, which is highly challenging for high-mass complexes (>1 MDa) with RNA bound and requires extensive buffer exchange that might induce dissociation.
To control the XL reactions, SDS-PAGE is usually the reference method because it is fast and robust visual method. However, for such samples it is also not very precise. Denaturing intact mass spectrometry is theoretically a possible approach for XL reaction control, but results in practice in highly heterogeneous and complex mass spectra for the following reasons: (i) high mass complexes (> 200 proteins, including PTMs) makes the MS data treatment complicated (overlapping signals of proteins and proteoforms), (ii) ionization issues related to high mass complexes’ analysis, even more with XL reagents and (iii) heterogeneity of the cross-linking reaction (multiple cross-linker additions). To tackle these issues, we recently developed and applied an approach called denaturing MP if to confirm the XL reaction as an alternative (Gizardin-Fredon et al. 2024, Nat. Comm.). It gives a fast readout over an extended mass range, with a low sample consumption and high throughput.

This explanation is acceptable; however, I note that Figure 3 (panels a and b) and Supplementary Figure 3 contain the same MP results for CAK sample. I suggest removing Supplementary Figure 3. For clarification, MP was performed for both the CAK sample and the spliceosome sample. Additionally, the Figure S20 legend contains a typographical error in panel d.

2) Manuscript will benefit if authors provide a direct comparison of the two methods at least for CAK samples: 4 internal CV method vs 4 external CV method using the same CVs with similar gradient length of 4 hrs and similar sample load. (same amount global length of injection). Authors did some comparison as in supplementary figure 1, but in this case internal CV gradient was equal to 1 external CV method or twice shorter and reported difference was probably due to instrument speed.

Answer: We thank the reviewer for this constructive suggestion. Following this remark, we performed additional experiments

to directly compare internal versus external CV stepping under equivalent analytical LC conditions. Specifically, we analyzed a newly prepared DSSO-cross-linked HeLa lysate using both 2-CV and 4-CV methods, ensuring comparable total separation times (2 h for 2-CV internal stepping and 4 h for 4-CV external stepping) and identical sample loading.
The results confirm that external CV stepping provides a consistent performance advantage even under matched acquisition times. We observed on average 25% increase in unique cross-link identifications for the 2-CV setup and a 50% increase for the 4-CV setup when using external stepping compared to internal stepping (see new graph in Supplementary Figure 2). While the magnitude of improvement is slightly reduced relative to shorter gradients, likely reflecting the greater overall sampling depth at longer gradient lengths, the benefit of external stepping remains significant and reproducible, reinforcing our conclusion that CV fractionation across independent runs provides superior identification depth.

I greatly appreciate that the authors considered my suggestion and performed 2- and 4-internal stepping CV experiments using comparable instrument time. However, after carefully reviewing the Methods section, I noticed that some experimental parameters do not appear to be optimal for internal stepping experiments. It would be helpful if the authors could confirm whether a dynamic exclusion setting of 60 seconds/CV was used for both types of experiments.
For reference, the recommended dynamic exclusion setting for FAIMS internal CV experiments is the global option, where a single global inclusion list is created, as this improves the identification of unique precursors. Additionally, it would be beneficial to include the number of CSMs in Supplementary Figure 2 to allow comparison of the overall identification rate and reinforce the main conclusion of this manuscript - the benefit of external stepping remains significant and reproducible.
One additional comment regarding the experimental setup: for the Orbitrap Eclipse operated at 300 nL/min, the recommended FAIMS total carrier gas flow is 3.9–4.0 L/min, rather than 4.6 L/min as stated in the manuscript.

3) Authors highlighted low amount of sample and short instrument time as additional benefits for new developed strategy and compared number of proteome level identified XL with obtained from SEC or SCX fractionation using higher amount of stating material as on page 13: At a proteome-wide level, the 11-CV FAIMS-GPF protocol using a total of 20 µg of starting material was able to provide 1269 XLs and 213 PPIs in less than 3 days, numbers that were previously only reached after extensive in-solution fractionation (e.g. using ≥ 20 strong cation exchange fractions for a total of ~500 µg of starting material and requiring weeks of sample prep and instrument time)27,28,62,63,81. Maybe this is accurate statement for the earlier results as in references 27& 28 but number of identified crosslinkers for example in reference 63 is significantly higher – it reported 4260 unique crosslinkers from 1357 proteins in 24 hrs of instrument time using 1 SEC fraction and 12 High pH fractions – please revise lines 371-373 accordingly to reflect current status of in-solution fractionation with/without FAIMS for proteome wide crosslinking analysis

Answer: We thank reviewer for this comment, we modified the text accordingly: “e.g. using ≥ 10 to 20 chromatography fractions for a total of > 500 to 100 µg of starting material and requiring days to weeks of sample prep and instrument time”. Additionally, the most sensitive protocol published, starting from 5 ug of HeLa lysate, identified 307 cross-linked peptides with DSSO using MICROFASP and tips-based SCX fractionation. Our FAIMS-GPF clearly outperforms (4-fold improvement) these results with similar or lower starting material amounts, while removing in-solution fractionation. This reference and sentences regarding these results were added in main text and in discussion (l.296: “Notably, our approach is the first to remove in-solution fractionation in proteome-wide protocols while outperforming by four folds the most sensitive workflow published up to now”; l.395: Finally, our approach also outperforms by 4-fold the most sensitive proteome-wide XL-MS protocol published up to now, which identified 307 XL-peptides from 5 µg of HeLa lysate using O-MICROFASP and tips-based SCX66)

Thank you for your explanation; however, the original text at line 294 still states “using >20 chromatography fractions.” In addition, for the comment at line 395 (“from 5 µg of HeLa lysate using O-MICROFASP and tip-based SCX66”), please correct the reference from 66 to 67.
Moreover, while it is indeed a remarkable result that more than 1,000 unique cross-linked peptides were identified without enrichment or in-solution fractionation from an injection of 5.5 µg total, the sample was prepared from 100 µg of HeLa cell lysate and therefore cannot be considered comparable to similar or lower starting material amounts mentioned above (Ref. 67). A starting material of 100 µg represents more favorable conditions for all sample prep steps, including cross-linking, reduction/alkylation, and digestion, compared with experiments performed using limited sample amounts.
I suggest comparing the proposed workflow with the workflow described in Ref. 63, as the starting material amount and overall experimental time are similar.

My minor comments were in general addressed except

- References 30 &36 are identical. Reference 30 is a preprint of reference 36 and needs to be replaced/merged by 36.
We thank reviewer for this remark, this has been modified in the text.
Unfortunately, there are still two identical references (References 30 and 33). Please replace Reference 30 with Reference 33(per review published manuscript).
In addition, I suggest carefully proofreading the manuscript, as there are still a significant number of typographical errors.

Version 2:

Reviewer comments:

Reviewer #1

(Remarks to the Author)
The authors answered all my questions and responded to my suggestions and subsequent experiments to my complete satisfaction. I wish them all the best.

Reviewer #2

(Remarks to the Author)

Reviewer #3

(Remarks to the Author)
I co-reviewed this manuscript with one of the reviewers who provided the listed reports. This is part of the Nature Communications initiative to facilitate training in peer review and to provide appropriate recognition for Early Career Researchers who co-review manuscripts.

Open Access This Peer Review File is licensed under a Creative Commons Attribution 4.0 International License, which permits use, sharing, adaptation, distribution and reproduction in any medium or format, as long as you give appropriate credit to the original author(s) and the source, provide a link to the Creative Commons license, and indicate if changes were made.
In cases where reviewers are anonymous, credit should be given to 'Anonymous Referee' and the source.
The images or other third party material in this Peer Review File are included in the article's Creative Commons license, unless indicated otherwise in a credit line to the material. If material is not included in the article's Creative Commons license and your intended use is not permitted by statutory regulation or exceeds the permitted use, you will need to obtain permission directly from the copyright holder.
To view a copy of this license, visit https://creativecommons.org/licenses/by/4.0/

Reviewer #1 (Remarks to the Author):

The authors of this publication introduce a crosslinking-MS pipeline that integrates FAIMS-based gas-phase fractionation (GPF) to enhance detection sensitivity of crosslinked peptides. The workflow reportedly offers superior coverage of protein-protein interactions (PPIs), reduces required sample quantities, and cuts acquisition time. Case studies include CAK (purified), HeLa lysate, and the spliceosome, benchmarking the 11-CV FAIMS-GPF protocol against unfractionated setups.

Major concerns:

1) Can the authors include a control experiment injecting a sample 11 times and compare the difference between those numbers with their 11CV-GPF workflow? It is known that DDA data produces more data with each injection of a technical replicate and with each injection the numbers of identified features will increase. This is due to the "random sampling" of DDA experiments. This is also true for crosslinking experiments. By injecting a sample two times and injecting the sample 10 times the identification rate increases by approximately 50%. It would be important to show that **gain for CV fractionation really results from the CVs instead of injecting a sample 11 times**. This can be easily tested by selecting the best working CV and injecting the sample 11 times and see how much each technical replicate increases the total number of crosslinks. If the gain of identification for both methods is the same, then this experimental set up does not give new insides.

Answer: We would like to thank Reviewer 1 for this insightful comment. It is indeed true that due to the stochastic nature of DDA sampling, performing multiple injection replicates can increase the number of identifications, particularly for low-abundance precursors such as cross-linked peptides. To evaluate this effect, we performed additional experiments under similar conditions and compared the 11-CV GPF method with two control setups: (i) 11 technical injection replicates acquired without FAIMS, and (ii) 11 injection replicates acquired using the best-performing single CV value (−55 V), see below.

Using a new batch of Hela cell lysates and using a comparable LC gradient length (90 min), we first identified 314 unique cross-links (XLs) without FAIMS and 401 with FAIMS in single injections. By performing 11 technical injection replicates, we identified 588 unique XLs without FAIMS and 675 using only the best-performing CV (−55 V). Notably, our 11-CV GPF approach substantially outperformed both conditions, yielding approximately a 3-fold increase compared to the FAIMS-free single injection and a 1.52-fold increase relative to the 11 replicate injections (1026 unique XLs identified). These results clearly demonstrate that the gain in identifications arises primarily from the CV fractionation strategy rather than from repeated sample injections. This Figure has been added as Supplementary Figure 12., and we added a related sentence in the text (l.230): "To ensure the impact of our approach we compared 11 injection replicates with 11-CV GPF, highlighting that improvement in identification comes mainly from GPF (**Supplementary Fig. 12**)."

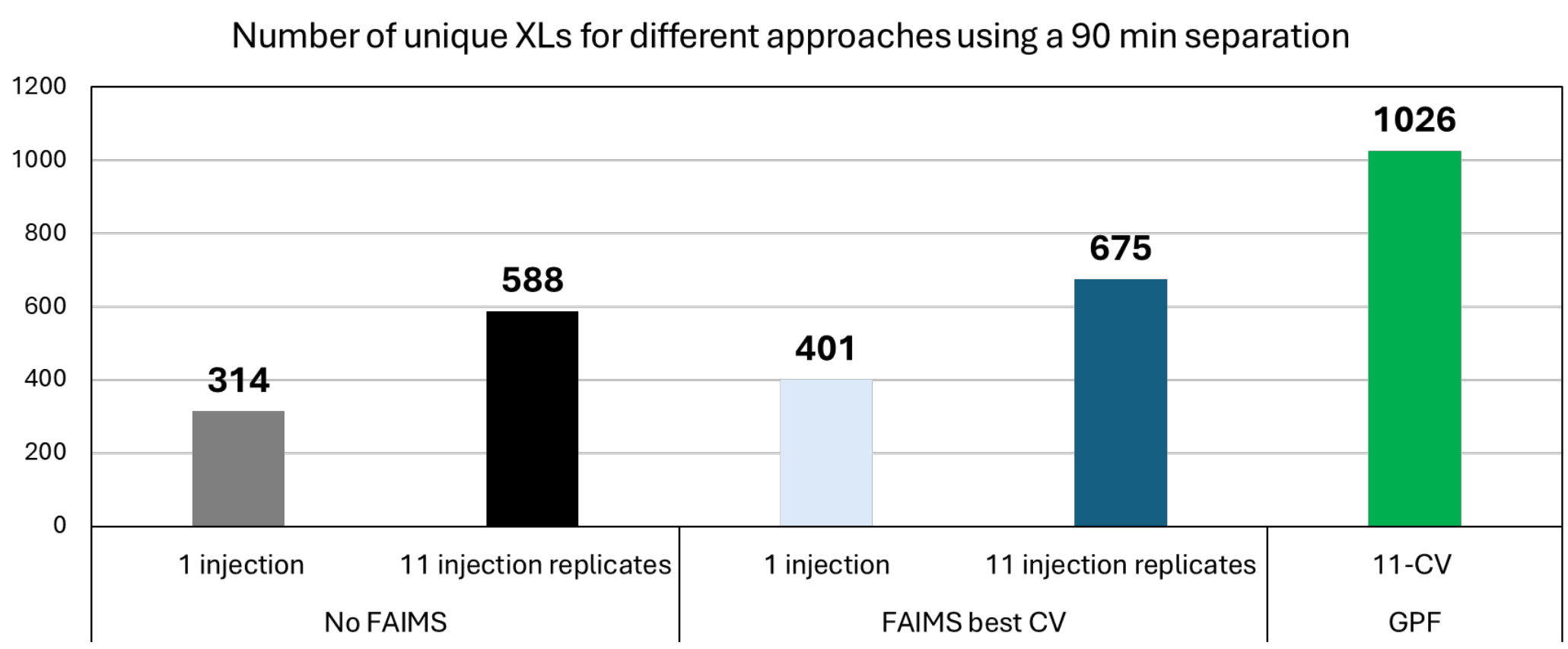

2) In comparison, similar performance using existing methods would require extensive in-solution

fractionations (> 20 fractions) using high amounts of starting material (> 100 µg per sample, usually around 500 µg)27,28,62–64." This statement is not true, since peptide fractionation can be also carried out with 50ug of stating material.

Answer: We thank Reviewer 1 for this remark. We fully agree that peptide fractionation can indeed be performed from relatively small amounts of starting material, down to a few micrograms in some classical proteomics workflows. However, the situation is notably different for cross-linked peptides. As demonstrated in the major XL-MS protocols published to date, substantially higher peptide quantities are typically required to achieve sufficient identification depth. Our comparison therefore focuses specifically on XL-MS workflows, not on standard proteomics fractionation setups.

For example, recent state-of-the-art XL-MS studies have used:

- Two-dimensional fractionation for proteome-wide XL-MS analysis (Jiao et al., Anal. Chem. 2022): **250 µg of peptides per replicate**.
- In vivo crosslinking and effective 2D enrichment for proteome-wide interactome studies (Bräuer et al., Commun. Chem., 2025): **1 mg per replicate.**
- Tips-based of LC-based SCX fractionation (Klykov et al., Nat. Comm, 2018): **100 µg minimum, with 500 µg** recommended for optimal recovery of low-abundance cross-linked peptides.
- SCX and bRPLC fractionation protocols (Wu et al., Anal. Chim. Acta 2021): **1 mg of dried peptides used for each separation step**.

These references have been added to the manuscript.

While some automated or tip-based microfractionation setups could, in principle, operate with ~50 µg of peptides, this remains an exception and is typically not suitable for proteome-wide XL-MS, especially when using microLC systems. Moreover, 50 µg is still roughly ten times higher than the amount required in our workflow, which also eliminates the need for in-solution fractionation, a key advantage, as this step is both sample-intensive and technically demanding. **In summary, our statement was made in the context of XL-MS methodologies, where large sample inputs are still the standard practice according to the published literature.**

Off note, the most sensitive protocol published, starting from 5 µg, identified 307 cross-linked peptides with DSSO using MICROFASP and SCX fractionation on lysates. Our FAIMS-GPF clearly outperforms (4-fold improvement) these results with similar or lower starting material amounts, while removing in-solution fractionation. This reference and a sentence regarding this reference was added in main text and in discussion (l.296: "Notably, our approach is the first to remove in-solution fractionation in proteome-wide protocols while outperforming by four folds the most sensitive workflow published up to now"; *l.395:* Finally, our approach also outperforms by 4-fold the most sensitive proteome-wide XL-MS protocol published up to now, which identified 307 XL-peptides from 5 µg of HeLa lysate using O-MICROFASP and tips-based SCX[66])

3) I would also argue that 3 days of measurement time for one sample is a lot of time and should be not over exaggerated as benefit here.

Answer: We would like to thank the reviewer for this remark. We first would like to add that usual proteome-wide experiments do not carry technical or biological replicates as we did. If we only consider one XL replicate, the instrument time is less than one day in our case, which is very short compared to published protocols using similar reagents:

- Liu et al. Nat. Comm. 2015: 20 SCX fractions, 60 hours per replicate
- Liu et al., Nat. Methd 2017: 21 SCX fractions, 63 hours per replicate
- Klykov et al., Nat. Prot., 2019: 20 SCX fractions, 24 h per replicate
- Yugandhar et al., Mol. and Cell. Prot., 2020 >300 fractions: 500 hours of instrument time

4) In the following publication the authors needed 1 day per fractionation experiment, which resulted in over 4000 unique links and about 300 PPIs (https://doi.org/10.1101/2025.02.25.640081).

Answer: We thank the reviewer for highlighting this recent study. We agree that this work achieved higher identification numbers. However, although the total experimental time is comparable, their workflow relies on a complex two-dimensional enrichment and in-solution fractionation strategy. In contrast, our approach does not aim to outperform such multi-step protocols in terms of absolute identification numbers, but rather to replace these labor-intensive and sample-demanding steps with a much simpler and more accessible sample preparation procedure. Our method thus prioritizes simplicity, reproducibility, and applicability over maximum depth obtained through extensive fractionation. A sentence has been added in the revised version of the manuscript.

5) I would like to point to another publication showing high PPIs for very low starting material amounts, which also shows higher numbers as reported here:
https://onlinelibrary.wiley.com/doi/epdf/10.1002/anie.202501845 (1426 crosslink sites per 1e6 SY5Y cells).

Answer: We thank the reviewer for bringing this publication to our attention. The numbers reported in this study are indeed comparable to those obtained with our workflow. However, the authors used a specialized, enrichable cross-linker that is not commercially available yet, whereas our approach relies solely on standard reagents and MS-based methods without any enrichment or in-solution fractionation. This comparison further underscores the strength of our workflow, which achieves similar identification depth (similar magnitude order: 1250 vs 1462) using a simpler, fully accessible, and broadly applicable protocol.

6) I am missing the reasoning for the newly 11CV-GPF developed method, it seems that the literature already outperforms the method here in terms of numbers and low starting material. Could the authors clarify or refine the benefit of their method?

Answer: We thank the reviewer for this important comment, to precise our rational, we added the following paragraph in discussion (l. 376): "While several recent protocols achieve impressive depth in proteome-wide XL-MS, their applicability remains limited by several key factors: (i) the large amounts of starting material required, (ii) complex and highly sample-specific preparation procedures that demand significant expertise and optimization, (iii) reliance on specialized enrichable reagents that are not always commercially available or easily adaptable, and (iv) extended instrument times due to extensive in-solution fractionation and long chromatographic gradients."

Our goal with the 11CV-GPF method is not to surpass existing protocols in absolute numbers of identifications, but rather to overcome these major practical limitations. The proposed workflow provides a versatile, fractionation-free, and easily transferable approach that can be applied to a wide range of sample types and complexities using only a few micrograms of material. This makes the method particularly valuable for non-expert laboratories, new XL-MS users, and core facilities where traditional in-solution fractionation or specialized reagents are not feasible.

As explained above, our approach outperforms by four folds the most sensitive workflow published up to now.

Importantly, to our knowledge, this represents the first proteome-wide XL-MS method that eliminates the need for any in-solution fractionation, while still achieving identification levels comparable to protocols that rely on complex multi-step workflows.

7) Numerous PPIs reported as novel—particularly those involving ENO1 and heat shock proteins—are not functionally validated by any orthogonal method. The claim that these represent biologically meaningful discoveries remains speculative in the absence of biochemical, genetic, or imaging-based evidence. This limits the strength of the manuscript's biological conclusions, especially for readers interested in translational relevance or mechanistic follow-up.

Answer: We fully agree that functional validation by orthogonal methods would be required to confirm the biological significance of the newly observed PPIs. However, the primary goal of this study is the development and validation of a methodological framework. Exhaustive biological

validation of specific interactions is out of the scope of the present study. Our discussion of novel PPIs, such as those involving ENO1 and heat shock proteins, is therefore intended to illustrate the depth and potential discovery of the 11CV-GPF approach, not to claim definitive biological findings.

Importantly, the biological relevance and robustness of our workflow are demonstrated through its application to well-characterized systems, including the CAK complex for structural biology applications and the spliceosome to bridge structural biology and interactomics. They serve as proof-of-concept examples supporting the accuracy and reproducibility of our method.

Some of the significant methodological publications in proteome-wide XL-MS where done using HeLa cells or other cell lines as HEK … and did not aim at drawing biological conclusions. They only used these cell lines as high-complexity sample as a proof of concept. We can cite the following references.

o Liu, F., Rijkers, D., Post, H. *et al.* Proteome-wide profiling of protein assemblies by cross-linking mass spectrometry. *Nat Methods* **12**, 1179–1184 (2015). - *HeLa cells*

o Klykov, O., Steigenberger, B., Pektaş, S. *et al.* Efficient and robust proteome-wide approaches for cross-linking mass spectrometry. *Nat Protoc* **13**, 2964–2990 (2018). - *PC9 cells*

o Jiao F., et al. Two-Dimensional Fractionation Method for Proteome-Wide Cross-Linking Mass Spectrometry Analysis. Anal. Chem. 2022, 94, 10, 4236–4242. - *HEK293*

o Liu, F., Lössl, P., Scheltema, R. *et al.* Optimized fragmentation schemes and data analysis strategies for proteome-wide cross-link identification. *Nat Commun* **8**, 15473 (2017). - *HeLa or E. coli*

o A. Wheat, C. Yu, X. Wang, A.M. Burke, I.E. Chemmama, R.M. Kaake, P. Baker, S.D. Rychnovsky, J. Yang, & L. Huang, Protein interaction landscapes revealed by advanced in vivo cross-linking–mass spectrometry, Proc. Natl. Acad. Sci. U.S.A. 118 (32) e2023360118, https://doi.org/10.*1073/pnas.2023360118 (2021). - HEK293*

Usually, in similar method development studies (see references above), authors do not use the results for biological interpretation but rather as an application proof-of-concept, linking known structures to XL-MS dataset, showing that they could recover known interaction and discover potential new ones.

8) A significant fraction of the crosslinks mapped onto structures (e.g., 26% in the CAK cryo-EM model) violate the commonly accepted 30 Å distance constraint for DSSO. The causes of these violations are not systematically examined—e.g., missing structure, domain flexibility, or model inaccuracy.

Answer: We thank reviewer 1 for his remark. It is indeed true that a few XLs violate the 30 A distance, which in absolute number only represents 5 XLs. First of all, we would like to point out that the used structure is incomplete which translate into relatively low number of XLs mapped. XL-MS experiments, especially for purified proteins, typically capture mixtures of conformations, while cryo-EM structures and predictions provide the most abundant and/or most probable one. Distance validation in structural biology works, even for highly pure or stable proteins/complexes never reach 100 %. In the revised version, we now examine with more details the different violated crosslinks, sentence added in the text (l. 181): "Of these, the K52-S164 XL can be explained by the flexibility of the T-loop of Cdk7 which can adopt by an alternative (inactive) conformation observed in the crystal structure of the isolated kinase (Lolli et al., 2004) and predicted in the AF model. The four remaining distance violations involve residues in regions well defined in the cryo-EM structure of CAK. As they cannot be explained by domain flexibility they probably arise from alternative and transient conformations captures in XL-MS experiments, which were not selected during EM images selection and model building.

9) Moreover, several mapped crosslinks rely on AlphaFold-predicted structures, but no confidence metrics or uncertainty considerations are provided for these models.

Answer: We thank the reviewer for this remark, we have added as a **Supp. Figure 6** the obtained confidence scores (pLDDT, pTM and iPTM) as well as coverage map and pLDDT per position obtained after AlphaFold prediction of CAK.

10) why different settings were used between the analysis? Sometimes linkage sites are set on K,S,T,Y sometimes only on K? This inconsistency makes comparisons between datasets problematic and raises concerns about methodological uniformity. The decision to use broader linkage rules in one case is not explained or controlled for.

Answer: We thank the reviewer for this important comment. We would like to clarify that we did not intend to directly compare the CAK, HeLa, and spliceosome datasets, as they serve distinct purposes within the study.

For purified protein complexes, we allowed cross-links on K, S, T, and Y residues (KSTY) since these datasets contain a limited number of cross-links that can be manually verified to ensure accurate site assignment (Iacobucci C., et al. Nat. Prot., 2018). Although lysine (K) remains the most probable reactive residue for DSSO, including additional potential sites (S, T, Y) provides a more comprehensive structural interpretation at this smaller scale, which is particularly interesting in structural biology.

In contrast, for proteome-wide experiments, we restricted linkage sites to lysine (K) only, following the practice established in several reference XL-MS protocols (Klykov et al. Nat. Meth., 2018; Clasen et al. Nat. Meth., 2025). This decision was made for three main reasons:

- To reduce computational and data analysis time, which becomes critical at proteome-wide scale.
- Because manual validation of linkage sites is not feasible with the large number of XLs and CSMs typically identified, and K linkage are the most probable.
- Recent studies (e.g., Cao et al., J. Proteome Res., 2023) have also shown that many S/T/Y assignments in large-scale datasets are likely false positives (usually corresponding to K-K cross-links from the same peptide pairs). In a recent preprint, Memetimin et al. (BiorXiv, 2025) could estimate that approximately 18% of STY cross-links identified in large-scale datasets are likely correct. Because the XLs cannot be checked be manually we preferred not to consider them in the data analysis of complex samples.

We included following sentences and references in the Materials and Methods section of the revised manuscript to clarify this rationale and ensure transparency in the parameter choices between datasets (l. 579): "For CAK we considered K, S, T, Y as DSSO linkage sites due to the relatively low number of spectra allowing manual validation. As recommended[28,64], due to the high number of spectra and to avoid false positives[87,88], only K was considered for spliceosome and proteome-wide samples."

11) if you report unique crosslinks the FDR should be also calculated on this level. CSM level FDR of 1% is not enough and produces most likely false identification on PPI level. It needs to be proven whether the novel links identified in this manuscript hold true if the FDR is calculated on PPI or at least on residue pair level. See have a look at the following publication:DOI: 10.1038/s41467-021-23666-z.

Answer: We thank the reviewer for this important remark. In our workflow, false discovery rate (FDR) control was applied at multiple levels using Proteome Discoverer (Thermo Fisher Scientific). Specifically, FDR was filtered at 1% for cross-link spectrum matches (CSMs), peptide pairs, and protein groups, as implemented in accordance with the widely accepted protocol by Klykov et al. (Nat. Protoc. 2018). These points have now been explicitly stated in the Materials and Methods section and clarified in the relevant figure legends.

While we acknowledge that residue-pair or PPI-level FDR estimation has been proposed (as discussed in the cited work (Lenz et al., Nat. Comm., 2021, DOI: 10.1038/s41467-021-23666-z), the 1% FDR threshold at the CSM/peptide level remains the standard practice in most recent large-scale XL-MS studies (e.g., Ihling et al., Anal. Chem. 2021; Klykov et al., Nat. Protoc. 2018).

We would also like to emphasize that applying 1% FDR at CSM, peptide, and PPI levels is fully aligned with the current best practices adopted in recent large-scale XL-MS studies (e.g., Anal. Chem. 2021, 93, 11442–11450; Nat. Protoc. 2018, 13, 2964–2990).

Moreover, we would like to point out that PPI-level FDR is usually not applicable for structural biology samples as it requires a high number of proteins to be relevant.

12) the manuscript highlights multiple novel interactions that are not present in STRING or other databases, but provides limited interpretation of their functional significance. These interactions are not placed within known pathways, cellular compartments, or network modules. Moreover, there is no analysis of whether these links reflect novel biology or potential false positives due to incomplete reference data.
Answer: **see also point (7)** - We would like to emphasize that the primary objective of this study is methodological development, rather than comprehensive biological interpretation. The mention of novel interactions not reported in STRING or other databases serves to illustrate the discovery potential and depth of the 11CV-GPF workflow, rather than to claim definitive biological findings.

To better highlight the biological applicability of our approach, we have expanded the discussion of the spliceosome dataset, which represents the main biological use case in this work. In addition, the CAK complex analysis is used as a benchmark to demonstrate the structural and quantitative accuracy of our method. Together, these examples show that our workflow can generate biologically meaningful data suitable for downstream functional studies, while the present manuscript focuses on establishing and validating the methodological framework.

Minor issues:
• Abbreviations such as "XL," "GPF," and "CV" are introduced without definition or used inconsistently between text and figures.

We thank the reviewer for pointing out inconsistencies. We have carefully revised the manuscript and made sure that these abbreviations are properly introduced.

• Several figure panels (e.g., Figure 3b, Figure 5a) have small fonts, complex color schemes, or insufficient axis labeling, which reduces interpretability.

We thank the reviewer for pointing out the readability issues, we refined our figures (including 3b and 5a) to increase the fonts and improve readability.

• Network and structure mapping figures use inconsistent or ambiguous color codes without clear legends or explanations in captions.

We thank the reviewer for pointing out the readability issues, we refined our figures and their to improve readability.

• The use of a 30 Å cutoff for Cα–Cα validation is not defended with theoretical or empirical rationale. Please cite at least a paper where the threshold was used before or better calculate the real distance cut of by using the length of the amino acid side chains (here lysine, STY depending on what the data is based on) and the crosslinker spacer length. A bit flexibility towards distance cut offs is possible but usually the crosslink distances are chosen to be overlength, which is an old artifact from the beginning of the crosslinking area.

Answer: We thank the reviewer for this insightful comment. As previously mentioned by the reviewer, using a 30 Å Cα-Cα cutoff for DSSO cross-link validation is indeed a **commonly accepted threshold within the XL-MS community**, as supported by multiple established and recent studies. This value has been consistently applied in key methodological papers and large-scale cross-linking workflows, including:

- **Merkley et al., Protein Sci. 2014** - provided a theoretical and empirical rationale through molecular dynamics simulations, showing that lysine-lysine distances compatible with closely related NHS-ester cross-linkers typically fall within a 25–30 Å range once side-chain flexibility is considered.
- **Klykov et al., Nat. Protoc. 2018** - the main DSSO protocol using the same software environment and validation approach.
- **Jiao et al., Anal. Chem. 2022** - from the Huang group, which originally developed DSSO, applying the same 30 Å criterion.
- **Hevler et al. open Biol. 2023** - This threshold was used by Heck´s group in a structural biology work

We have now added these citations in the manuscript to explicitly justify the use of the 30 Å threshold (l. 592)

In addition, several **recent large-scale XL-MS studies** have even used distance threshold with **more relaxed cutoffs (up to 40 Å)**:

- **Zhu et al., Nat. Commun. 2024** - applied a 40 Å cutoff for DSSO.
- **Bogdanow et al., Nat. Microbiol. 2023** - used a similar 40 Å limit.

These examples illustrate that a modest flexibility in distance cutoffs is now broadly recognized as appropriate for in-solution conformational variability.

Finally, we note that **cross-links involving serine, threonine, or tyrosine (STY)** residues have **shorter side chains than lysine**, and therefore the corresponding Cα-Cα distances will inherently fall within, or below, this 30 Å limit.

In summary, our use of a 30 Å threshold is well supported both theoretically and empirically, and consistent with recent practice in the field.

• The Eclipse is a high mass accuracy instrument (when well calibrated), 10ppm MS1 and 20ppm MS2 seems to be too loose.

Answer: We thank the reviewer for this remark. We agree with the reviewer that for classical quantitative proteomics lower mass tolerances are usually applied. However, and due to the different nature of XL peptides, the **10 ppm (MS1)** and **20 ppm (MS2)** mass tolerances were chosen in accordance with the **current best practices for XL-MS data analysis** on Orbitrap Tribrid instruments, including the Eclipse platform. These exact parameters have been applied in several recent **reference studies** published in major journals using comparable instrument configurations:

- **Klykov et al., Nat. Protoc. 2018**: Orbitrap Fusion Tribrid using MS1 set to 20 ppm and MS2 set to 20 ppm
- **Bartolec et al., PNAS 2020:** Orbitrap Fusion Tribrid using 10 ppm in MS1 and 20 ppm in MS2
- **Clasen et al., Nat. Methods 2024**, Orbitrap Fusion Lumos mass spectrometer with FAIMS Pro, using 10 ppm for MS1 and 20 ppm for MS2
- **Zhu et al., Nat. Commun. 2024**, Orbitrap Fusion Lumos using 10 ppm for MS1 and 20 ppm for MS2

We therefore consider our parameters to be fully in line with current community standards. References were added in the methods sections too (l. 582).

• What is the rational to set the charge states to +2 to +7 for a linear proteomics MS method? **Answer**: We thank the reviewer for this question. The charge-state range of **+2 to +7** was chosen to ensure comprehensive detection of both **tryptic peptides** and **cross-linked or partially digested species** under identical LC-MS conditions.

In proteomics workflows, **+2** is universally used as the lower bound, as doubly charged ions represent the predominant charge state for linear tryptic peptides and are essential for reliable MS/MS identification. The **upper limit of +7** was selected to accommodate potential **higher-charged species** that can arise from (i) missed cleavages, (ii) longer peptides, or (iii) cross-linked peptides present in the same sample preparation.

While +7 ions are relatively rare in linear peptide datasets, including them **does not negatively affect acquisition speed or cycle time**, and ensures that no valid identifications are excluded a priori. This charge-state window is also **consistent with parameters used in recent large-scale XL-MS and proteomics workflows** on Orbitrap Tribrid instruments (e.g., Klykov et al., Nat. Protoc. 2018; Clasen et al., Nat. Methods 2024).

Thus, the +2 to +7 setting was chosen as a **broad but standard range** that guarantees full coverage across both conventional linear peptides and potential cross-linked species generated in the same samples.

Reviewer #2:
The study presented by Gizardin-Fredon et al outlines the utility of gas-phase fractionation for mass spec crosslinking analysis. Improving dynamic range/sensitivity of detection of crosslinked peptides/proteins is a hot topic currently and there is a need for new crosslinker chemistries as well as analytical methods. There are several publications which demonstrated the utility of gas fractionation using FAIMS device. Moreover, such a method using several FAIMS CVs in one run(internal CV stepping), is considered a standard method for XL-MS to improve dynamic range usually in combination with enrichment, in-solution offline fractionation. Authors suggested that single FAIMS CV fractionation- external CV stepping method provides alternative solution and enables the same depth of analysis as multiple CVs FAIMS method in combination with offline fractionation. Overall, this study highlights the use of FAIMS for crosslinking analysis and provides some novelty which can be beneficial for the community after addressing the following points:

Major concerns
1) What is exactly the role of Quality Control (QC) by Mass Photometry (MP) prior crosslinking? Why denatured MP was performed vs denatured intact MS analysis. Looks like native /denatured MS analysis will provide the same QC data and was actually used in some experiments.

Answer: We would like to thank the reviewer for this question. In this study we first use mass photometry in native conditions as an initial sample quality assessment. Thus, before cross-linking reaction, mass photometry is carried on intact spliceosome in its native buffer to assess its integrity. It is very useful as an alternative to native-MS, which is highly challenging for high-mass complexes (>1 MDa) with RNA bound and requires extensive buffer exchange that might induce dissociation.

To control the XL reactions, SDS-PAGE is usually the reference method because it is fast and robust visual method. However, for such samples it is also not very precise. Denaturing intact mass spectrometry is theoretically a possible approach for XL reaction control, but results in practice in highly heterogeneous and complex mass spectra for the following reasons: (i) high mass complexes (> 200 proteins, including PTMs) makes the MS data treatment complicated (overlapping signals of proteins and proteoforms), (ii) ionization issues related to high mass complexes' analysis, even more with XL reagents and (iii) heterogeneity of the cross-linking reaction (multiple cross-linker additions). To tackle these issues, we recently developed and applied an approach called denaturing MP if to confirm the XL reaction as an alternative (Gizardin-Fredon et al. 2024, Nat. Comm.). It gives a fast readout over an extended mass range, with a low sample consumption and high throughput.

2) Manuscript will benefit if authors provide a direct comparison of the two methods at least for CAK samples: 4 internal CV method vs 4 external CV method using the same CVs with similar gradient length of 4 hrs and similar sample load. (same amount global length of injection). Authors did some comparison as in supplementary figure 1, but in this case internal CV gradient was equal to 1 external CV method or twice shorter and reported difference was probably due to instrument speed.

Answer: We thank the reviewer for this constructive suggestion. Following this remark, we performed additional experiments to directly compare internal versus external CV stepping under equivalent analytical LC conditions. Specifically, we analyzed a newly prepared DSSO-cross-linked HeLa lysate using both 2-CV and 4-CV methods, ensuring comparable total separation times (2 h for 2-CV internal stepping and 4 h for 4-CV external stepping) and identical sample loading.

The results confirm that external CV stepping provides a consistent performance advantage even under matched acquisition times. We observed on average 25% increase in unique cross-link identifications for the 2-CV setup and a 50% increase for the 4-CV setup when using external stepping compared to internal stepping (see new graph in **Supplementary Figure 2**).

While the magnitude of improvement is slightly reduced relative to shorter gradients, likely reflecting the greater overall sampling depth at longer gradient lengths, the benefit of external stepping remains significant and reproducible, reinforcing our conclusion that CV fractionation across independent runs provides superior identification depth.

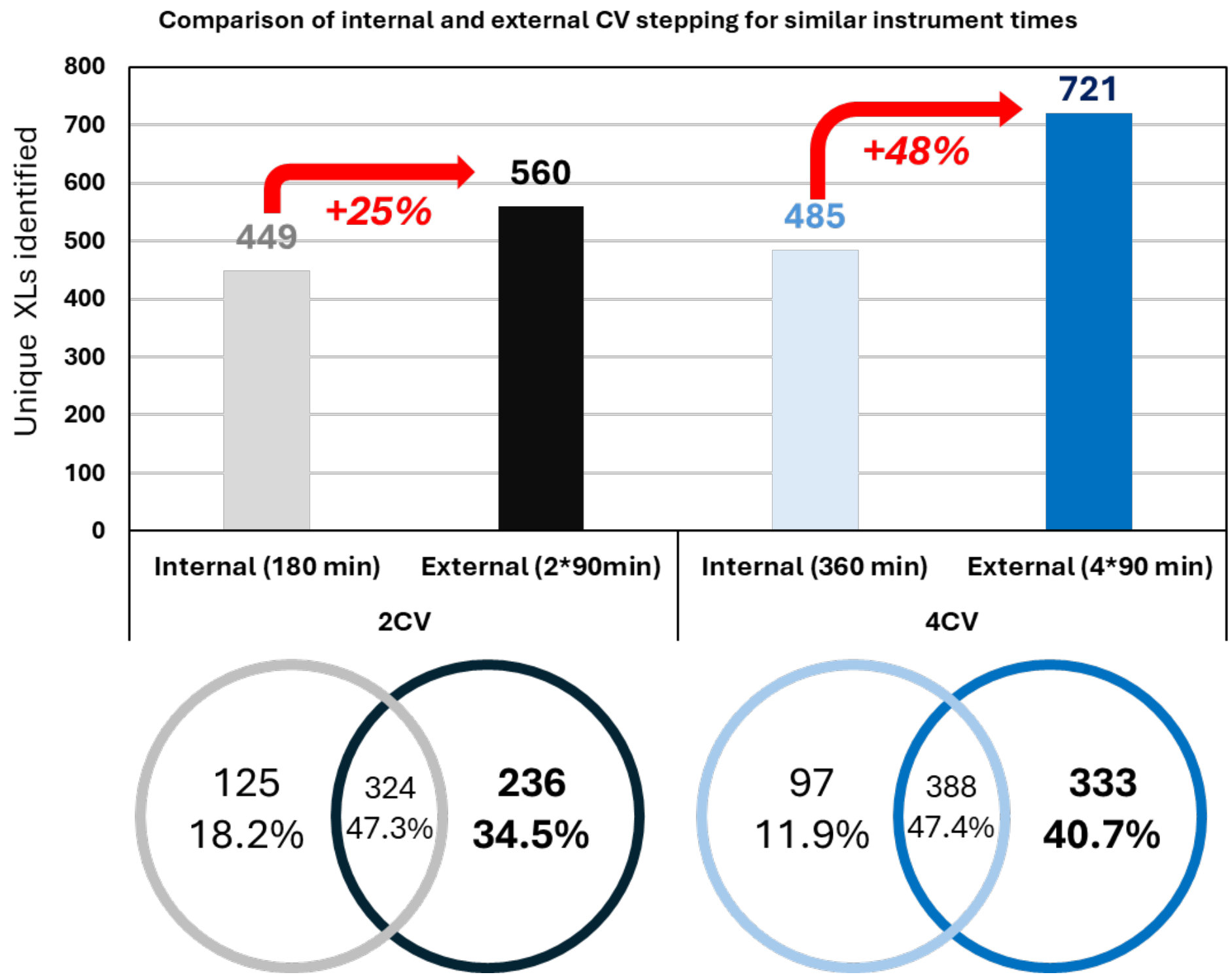

3) Authors highlighted low amount of sample and short instrument time as additional benefits for new developed strategy and compared number of proteome level identified XL with obtained from SEC or SCX fractionation using higher amount of stating material as on page 13: At a proteome-wide level, the 11-CV FAIMS-GPF protocol using a total of 20 µg of starting material was able to provide 1269 XLs and 213 PPIs in less than 3 days, numbers that were previously only reached after extensive in-solution fractionation (e.g. using ≥ 20 strong cation exchange fractions for a total of ~500 µg of starting material and requiring weeks of sample prep and instrument time)27,28,62,63,81. Maybe this is accurate statement for the earlier results as in references 27& 28 but number of identified crosslinkers for example in reference 63 is significantly higher – it reported 4260 unique crosslinkers from 1357 proteins in 24 hrs of instrument time using 1 SEC fraction and 12 High pH fractions – please revise lines 371-373 accordingly to reflect current status of in-solution fractionation with/without FAIMS for proteome wide crosslinking analysis

Answer: We thank reviewer for this comment, we modified the text accordingly: "e.g. using ≥ 10 to 20 chromatography fractions for a total of > 500 to 100 µg of starting material and requiring days to weeks of sample prep and instrument time". Additionally, the most sensitive protocol published, starting from 5 ug of HeLa lysate, identified 307 cross-linked peptides with DSSO using MICROFASP and tips-based SCX fractionation. Our FAIMS-GPF clearly outperforms (4-fold improvement) these results with similar or lower starting material amounts, while removing in-solution fractionation. This reference and sentences regarding these results were added in main text and in discussion (l.296: "Notably, our approach is the first to remove in-solution fractionation in proteome-wide protocols while outperforming by four folds the most sensitive workflow published up to now"; *l.395:* Finally, our approach also outperforms by 4-fold the most sensitive proteome-wide XL-MS protocol published up to now, which identified 307 XL-peptides from 5 µg of HeLa lysate using O-MICROFASP and tips-based SCX[66])

Additionally, I have few **minor comments**:
- This sentence on page 4 needs to be corrected "such as sulfoxide-based DSSO22 and DSBU23", DSBU crosslinker is not sulfoxide based.

We thank reviewer for this remark, this has been modified in the text.

- References 30 &36 are identical. Reference 30 is a preprint of reference 36 and needs to be replaced/merged by 36.

We thank reviewer for this remark, this has been modified in the text.

- Page 9 line 235-236 – how many runs of unfractionated HeLa without FAIMS were performed 3 per biological replicate or just one

We thank reviewer for this question, one LC-MS/MS **run was done per XL replicate.**

- Page 9 line 240-241 – typo 2.8 times increase not time same for line 241- 3.6 times increase not time.
We thank reviewer for this remark, this has been modified in the text.

REVIEWER COMMENTS

Reviewer #1 (Remarks to the Author):

I would like to express my sincere thanks to the authors for their detailed and extensive work in answering my questions as thoroughly as possible.
With the exception of question 11, I am very satisfied with the answers. Thank you again for your efforts.

Now I would like to explain my arguments for question 11 in more detail.
The software node used in Proteome Discoverer is called XlinkX and was originally developed by Fan Liu's group.
We thank the reviewer for their positive assessment of our revisions and for further elaborating on their concerns regarding FDR estimation. We fully agree that accurate FDR control is critical for ensuring reliable cross-link identifications.

Several publications, including those by the Rappsilber group, have shown that the FDR of XlinkX is very high at nearly 40%. As a result, an improved version was developed, which, however, still has a very high FDR rate.
Regarding the ability of XlinkX to properly control FDRs, we would like to provide additional context to clarify its applicability to our study. As correctly pointed out, XlinkX was originally developed by Fan Liu during her time in Albert Heck's laboratory but was integrated into Proteome Discoverer through contributions from Albert Heck and Richard Scheltema. We agree that 2 studies from the Rappsilber group, have reported elevated FDRs under certain conditions (Lutz et al. 2025 bioRxiv https://doi.org/10.1101/2025.04.27.649519; Lutz, Rappsilber, 2018 PLOS One https://doi.org/10.1371/journal.pone.0196672).
However, an important aspect to consider is that these studies were performed under experimental settings that differ significantly from ours. In particular, the Rappsilber group evaluated specifically non-cleavable cross-linkers such as EDC or SDA, which are known to provide lower linkage specificity and therefore inherently higher false-positive rates. In contrast, our study exclusively employs the MS-cleavable cross-linker DSSO that has been shown to significantly improve identification confidence (Kolbowski et al., 2022, Anal. Chem. https://doi.org/10.1021/acs.analchem.1c05266, Müller et al., 2010, Anal. Chem. https://doi.org/10.1021/ac101241t). To be noted that XlinkX applies distinct algorithms depending on whether cleavable or non-cleavable cross-linkers are used (Fig. 1), making a direct comparison between these datasets less straightforward.

[editorial note: third party material redacted]

Figure 1: XlinkX algorithms in PD

We also note that one key recommendation from the Rappsilber group is to control FDR separately for intra- and inter-protein cross-links (https://doi.org/10.1038/s41467-021-23666-z). This strategy was implemented in our workflow and represents an important factor in improving FDR accuracy. Notably, this separation was not consistently applied in the preprint supposed to be referenced by the reviewer.
Furthermore, recent work from Fan Liu's group (Bogdanow et al., 2021, Nat. Comm. https://doi.org/10.1038/s44320-024-00079-w) provides additional support for our approach. In

this study, reanalysis of multiple datasets - including DSSO-based experiments - demonstrated that when intra- and inter-links are treated as separate populations within a concatenated target-decoy strategy, the estimated FDR closely matches the experimentally observed FDR. Interestingly, the authors also report that false negatives tend to be higher than false positives under these filtering conditions, suggesting that such workflows may be conservative rather than permissive (Fig. 2)

[editorial note: third party material redacted]

Figure 2

Similarly, benchmarking work using synthetic ribosomal proteins (Matzinger et al., 2022, Nat. Comm. https://doi.org/10.1038/s41467-022-31701-w) evaluated experimental FDR in DSSO datasets analyzed with XlinkX. Even without separating intra- and inter-links and using only CSM-level filtering, the observed experimental FDR did not exceed 5% when applying a 1% FDR threshold (Fig. 3)

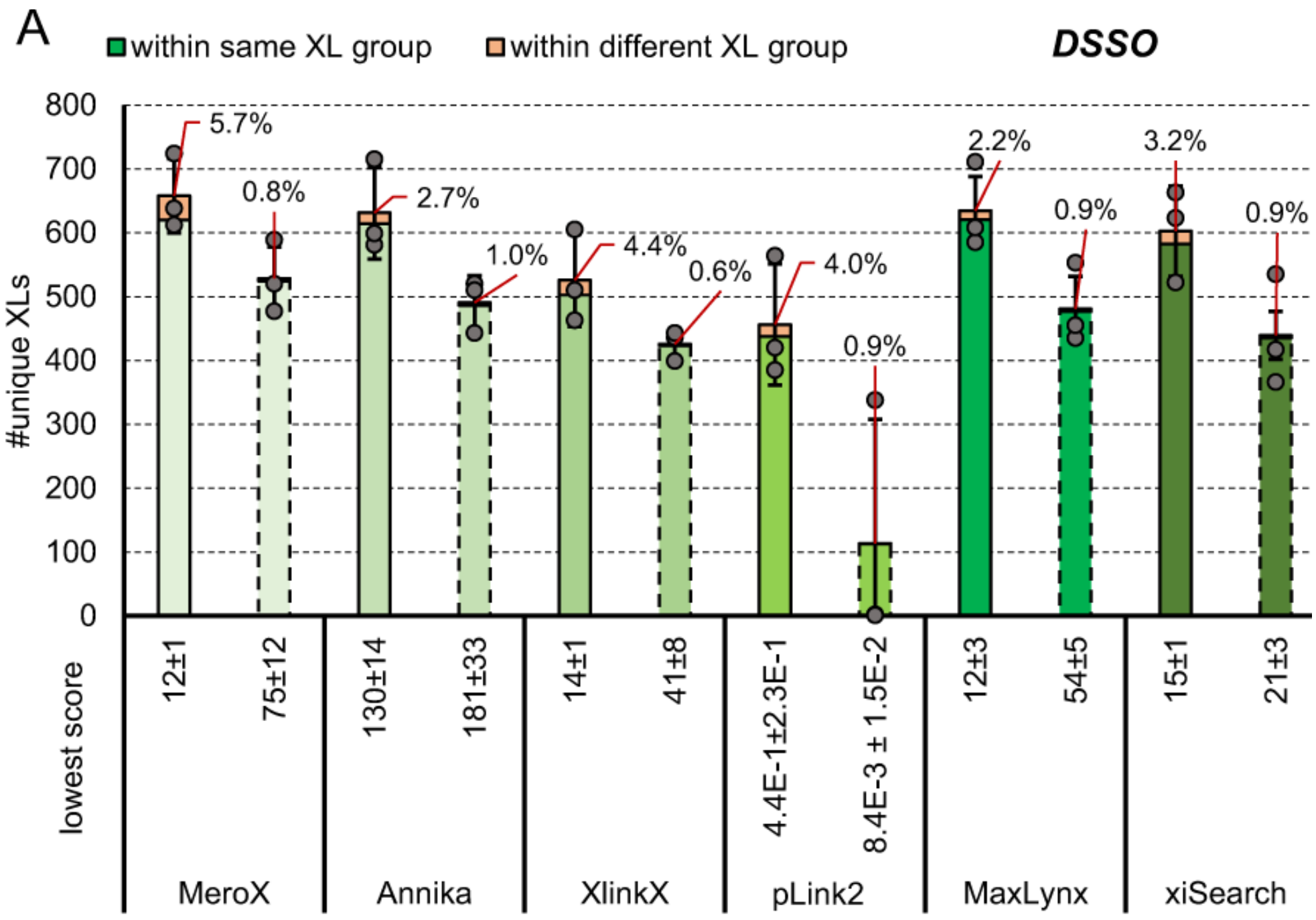

Figure 3

Taken together, these studies indicate that, for DSSO-based datasets and when applying appropriate FDR strategies, including separation of intra- and inter-links, XlinkX provides reliable and confident cross-link identifications, with experimental FDR matching requirements. Therefore, while we appreciate the reviewer's concern, we believe that the conclusions drawn from studies using non-cleavable cross-linkers are not directly transferable to our experimental setup.

In summary, we are confident that our FDR control strategy is appropriate, in line with current best practices, and supports the robustness of our results.

It is also a fact that the original developer of the software no longer uses their own software,

which was published several years ago, but instead uses newly developed software at her lab called Scout. https://github.com/theliulab/Scout
We thank the reviewer for raising this important point regarding the evolution of software tools in cross-linking mass spectrometry.
We acknowledge that the Liu laboratory has developed newer tools, such as Scout, which reflects the natural progression of computational methods in the field. The development of alternative software solutions is common and contributes to improving data analysis strategies.
However, we would like to emphasize that XlinkX remains widely used and actively applied in recent high-impact studies, including from the Heck and Scheltema laboratories, which were central to its development and continued optimization. For example, XlinkX has been used in several recent publications:

- Kadavá et al., *EMBO Journal* (2024)
- Leung et al., *Cell* (2023)
- Milenkovic et al., *Cell Metabolism* (2023)
- Hevler et al., *Open Biology* (2023)
- recent preprint on ATP synthase crosslinking, 2025

These examples illustrate that XlinkX remains a validated and trusted tool within the community, including by groups closely associated with its development.
More broadly, the adoption of newer software (such as Scout) does not invalidate previous tools, but rather reflects ongoing methodological innovation. Different tools may offer complementary strengths depending on the dataset, experimental design, and specific analysis goals. In our case, XlinkX is fully integrated into Proteome Discoverer, supports MS-cleavable cross-linkers such as DSSO, and provides a well-established and reproducible workflow aligned with our study design.
Therefore, while we recognize the emergence of newer tools, we consider XlinkX to remain an appropriate, robust, and community-validated choice for our analysis.

In response to the authors' reply, I would also like to note that best practice in 2018 will no longer be best practice in 2026, as the field has also developed enormously.
We thank the reviewer for this important remark and fully agree that the field of cross-linking mass spectrometry has evolved considerably in recent years, including both experimental and computational aspects.
At the same time, while several community-driven efforts have aimed to improve reliability, transparency, and reporting standards (e.g., A Roadmap for Improving Reliability and Data Sharing in Crosslinking Mass Spectrometry; Toward Increased Reliability, Transparency, and Accessibility in Cross-linking Mass Spectrometry), these works primarily focus on data quality, FDR control, and reporting practices. To our knowledge, they do not establish specific recommendations regarding the mandatory use of particular search engines or software tools. Thus, despite the rapid development of new computational approaches, there is currently no broadly accepted community guideline defining a “best practice” in terms of software choice for cross-link identification. Instead, the emphasis remains on the appropriate use of validated workflows, transparent reporting, and robust error control strategies.
In this context, we believe that our analysis, based on a well-established and widely used workflow with appropriate FDR control, is consistent with current standards in the field.

I would therefore ask you to search for the data using a second independent software node called MS Anika, which was also developed for Proteome Discoverer, and compare the

results.
Since MS-Anika is integrated as simple node into Proteome Discoverer, re-analysis should not be too difficult, and the additional data can answer my above question to my complete satisfaction.

We thank the reviewer for this constructive suggestion and for emphasizing the continuous evolution of best practices in cross-linking mass spectrometry.

Based on our previous responses, we would like to clarify that the concern regarding a potentially high FDR in our dataset appears to rely on assumptions that may not fully apply to our experimental conditions, particularly given our use of DSSO and dedicated FDR control strategies (including separation of intra- and inter-links). As discussed above, recent studies suggest that, under such conditions, the estimated FDR closely reflects the experimental error rate.

Nevertheless, we took the reviewer's suggestion seriously and still performed an exploratory re-analysis of our dataset using MS Annika within Proteome Discoverer. This analysis resulted in 986 cross-links identified with MS Annika compared to 1278 with XlinkX. While a subset of high-confidence cross-links is consistently identified by both tools, the overall overlap remains limited. As an example, regarding inter-XLs, we retrieved an overlap of 16 % between the 213 identified in XlinkX and the 62 identified in MS Annika. We also remarked that a more detailed comparison between both algorithms, which is not the topic of our article, would be needed as XlinkX and MS Anika outputs are different and do not allow us to draw useful conclusions as they are. Importantly, this discrepancy reflects inherent differences between search engines, including scoring functions, identification strategies, specific linkage sites, and FDR estimation procedures. As a result, direct comparison between outputs is not straightforward and does not in itself allow conclusions to be drawn regarding the correctness of identifications or the underlying FDR without a dedicated benchmarking framework.

We would also like to point out that current community recommendations (e.g., A Roadmap for Improving Reliability and Data Sharing in Crosslinking Mass Spectrometry; Toward Increased Reliability, Transparency, and Accessibility in Cross-linking Mass Spectrometry) emphasize appropriate FDR control and transparency, but do not require the use of multiple search engines as standard practice.

Finally, our study is focused on assessing the impact of FAIMS on cross-link identification. In this context, maintaining a consistent data analysis workflow is essential to ensure comparability across conditions, and introducing a second search engine would not directly address the reviewer's concern regarding FDR. That is why we prefer not to include the MS Annika search results in the supplementary data of this article

Taken together, while we appreciate the reviewer's suggestion and have explored it, the differences observed between software outputs make it difficult to draw meaningful conclusions regarding FDR from such a comparison alone. A systematic evaluation of search engines would require a dedicated study beyond the scope of the present work. We are, however, be happy to make our raw data available to support such future benchmarking efforts.

Reviewer #2 (Remarks to the Author):

The authors provided detailed responses to my concerns, and overall I can see improvements in the clarity of the writing and the results. However, some of my concerns and typographical errors were not completely addressed or corrected.

Major concerns

1) What is exactly the role of Quality Control (QC) by Mass Photometry (MP) prior crosslinking? Why denatured MP was performed vs denatured intact MS analysis. Looks like native /denatured MS analysis will provide the same QC data and was actually used in some experiments.

We thank the reviewer for this important question and the opportunity to clarify the role of mass photometry (MP) in our workflow.

In this study, mass photometry under native conditions is used as an initial quality control step prior to cross-linking. Specifically, MP allows us to assess the integrity, homogeneity, and assembly state of the intact spliceosome directly in its native buffer. This is particularly advantageous for very large ribonucleoprotein complexes (>1 MDa), for which native mass spectrometry remains technically challenging due to requirements for extensive buffer exchange and the risk of complex dissociation.

Following cross-linking, the objective of quality control is different: rather than assessing structural integrity, the goal is to monitor the extent and consistency of the cross-linking reaction. While SDS-PAGE is commonly used for this purpose due to its speed and robustness, its resolution is limited for very large and heterogeneous assemblies such as the spliceosome. In principle, denaturing intact mass spectrometry could be used to monitor cross-linking. However, in practice, it is poorly suited for this application in our system due to the additional heterogeneity introduced by cross-linking (variable numbers of modifications per protein), which further complicates spectral interpretation.

To address these limitations, we employed denaturing mass photometry that we recently described (Gizardin-Fredon et al., 2024, *Nature Communications*). This approach provides a rapid and global readout of the mass distribution of the sample before and after cross-linking, enabling us to directly observe mass shifts associated with cross-linker incorporation. In this context, MP offers a simple, low-sample-consumption, and high-throughput method to monitor cross-linking efficiency across a broad mass range.

In summary, native MP is used to assess sample integrity prior to cross-linking, while denaturing MP provides a practical and informative quality control of the cross-linking reaction itself. Although mass spectrometry remains more precise for the analysis of non-cross-linked samples, it is not well adapted for routine monitoring of cross-linking reactions in highly complex assemblies such as the spliceosome.

This explanation is acceptable; however, I note that Figure 3 (panels a and b) and Supplementary Figure 3 contain the same MP results for CAK sample. I suggest removing Supplementary Figure 3. For clarification, MP was performed for both the CAK sample and the spliceosome sample. Additionally, the Figure S20 legend contains a typographical error in panel d.

We thank the reviewer for this remark this has been corrected.

2) Manuscript will benefit if authors provide a direct comparison of the two methods at least for CAK samples: 4 internal CV method vs 4 external CV method using the same CVs with similar gradient length of 4 hrs and similar sample load. (same amount global length of injection). Authors did some comparison as in supplementary figure 1, but in this case internal CV gradient was equal to 1 external CV method or twice shorter and reported difference was probably due to instrument speed.

Previous answer: We thank the reviewer for this constructive suggestion. Following this remark, we performed additional experiments to directly compare internal versus external CV stepping under equivalent analytical LC conditions. Specifically, we analyzed a newly prepared DSSO-cross-linked HeLa lysate using both 2-CV and 4-CV methods, ensuring comparable total separation times (2 h for 2-CV internal stepping and 4 h for 4-CV external stepping) and identical sample loading.
The results confirm that external CV stepping provides a consistent performance advantage even under matched acquisition times. We observed on average 25% increase in unique cross-link identifications for the 2-CV setup and a 50% increase for the 4-CV setup when using external stepping compared to internal stepping (see new graph in Supplementary Figure 2). While the magnitude of improvement is slightly reduced relative to shorter gradients, likely reflecting the greater overall sampling depth at longer gradient lengths, the benefit of external stepping remains significant and reproducible, reinforcing our conclusion that CV fractionation across independent runs provides superior identification depth.

I greatly appreciate that the authors considered my suggestion and performed 2- and 4-internal stepping CV experiments using comparable instrument time. However, after carefully reviewing the Methods section, I noticed that some experimental parameters do not appear to be optimal for internal stepping experiments. It would be helpful if the authors could confirm whether a dynamic exclusion setting of 60 seconds/CV was used for both types of experiments. For reference, the recommended dynamic exclusion setting for FAIMS internal CV experiments is the global option, where a single global inclusion list is created, as this improves the identification of unique precursors.
We thank the reviewer for this helpful remark, which indeed improves the clarity and reproducibility of our experimental description.
We confirm that a dynamic exclusion time of 60 seconds was used consistently across all experiments, including both 2-CV and 4-CV internal stepping acquisitions.
We would like to clarify that, in our setup, dynamic exclusion is applied at the level of the data-dependent acquisition (TopN method) rather than being defined independently for each compensation voltage (CV). As such, the exclusion time is not CV-specific but instead governs the reselection of precursor ions across the full acquisition cycle.
Our choice of a 60 s exclusion time is in line with previously reported FAIMS-DDA workflows on Orbitrap platforms and was selected as a balanced parameter to limit repeated sequencing while maintaining sufficient sampling depth. We agree that the use of a global exclusion strategy is beneficial in FAIMS experiments to improve the sampling of unique precursors across different CVs. In our setup, this behavior is inherently achieved through the instrument control of dynamic exclusion within the acquisition cycle.
Regarding cycle timing, under our acquisition conditions (Top10 method and selected resolution), a full MS/MS cycle is on the order of ~1 second. This ensures efficient precursor sampling while the 60 s exclusion window effectively prevents repeated sequencing of the same ions within every CV steps.
Overall, we believe that our settings provide an appropriate balance between depth of coverage and redundancy control and are well suited to the FAIMS internal stepping strategy used in this study.

Additionally, it would be beneficial to include the number of CSMs in Supplementary Figure 2 to allow comparison of the overall identification rate and reinforce the main conclusion of this manuscript - the benefit of external stepping remains significant and reproducible.

We thank the reviewer for his remark and added the number of CSMs in the Supplementary Figure 2.

One additional comment regarding the experimental setup: for the Orbitrap Eclipse operated at 300 nL/min, the recommended FAIMS total carrier gas flow is 3.9–4.0 L/min, rather than 4.6 L/min as stated in the manuscript.

We thank the reviewer for pointing out the recommended FAIMS carrier gas flow range for this configuration.

We agree that a flow rate of 3.9-4.0 L/min is commonly reported for Orbitrap Eclipse systems operated at similar nanoLC flow rates. However, in our laboratory, we already systematically optimized FAIMS parameters for our specific experimental setup, including column configuration, gradient conditions, and sample type. These optimizations, performed during the PhD work of Dr. Corentin Beaumal and subsequently validated in our lab, identified a slightly higher carrier gas flow (~4.7 L/min) as providing improved performance in terms of signal intensity and cross-link identification yield under our conditions. This setting has been consistently used in our recent work (e.g., Beaumal *et al*. Proteomics 2023).

We would like to emphasize that FAIMS performance is known to be sensitive to experimental conditions, and optimal parameters may therefore vary between laboratories and setups. In this context, the value reported in our manuscript reflects an empirically optimized condition rather than a generic recommendation.

3) Authors highlighted low amount of sample and short instrument time as additional benefits for new developed strategy and compared number of proteome level identified XL with obtained from SEC or SCX fractionation using higher amount of stating material as on page 13: At a proteome-wide level, the 11-CV FAIMS-GPF protocol using a total of 20 µg of starting material was able to provide 1269 XLs and 213 PPIs in less than 3 days, numbers that were previously only reached after extensive in-solution fractionation (e.g. using ≥ 20 strong cation exchange fractions for a total of ~500 µg of starting material and requiring weeks of sample prep and instrument time)27,28,62,63,81. Maybe this is accurate statement for the earlier results as in references 27& 28 but number of identified crosslinkers for example in reference 63 is significantly higher – it reported 4260 unique crosslinkers from 1357 proteins in 24 hrs of instrument time using 1 SEC fraction and 12 High pH fractions – please revise lines 371-373 accordingly to reflect current status of in-solution fractionation with/without FAIMS for proteome wide crosslinking analysis

Previous answer: We thank reviewer for this comment, we modified the text accordingly: “e.g. using ≥ 10 to 20 chromatography fractions for a total of > 500 to 100 µg of starting material and requiring days to weeks of sample prep and instrument time”. Additionally, the most sensitive protocol published, starting from 5 ug of HeLa lysate, identified 307 cross-linked peptides with DSSO using MICROFASP and tips-based SCX fractionation. Our FAIMS-GPF clearly outperforms (4-fold improvement) these results with similar or lower starting material amounts, while removing in-solution fractionation. This reference and sentences regarding these results were added in main text and in discussion (l.296: “Notably, our approach is the first to remove in-solution fractionation in proteome-wide protocols while outperforming by four folds the most sensitive workflow published up to now”; l.395: Finally, our approach also outperforms by 4-fold the most sensitive proteome-wide XL-MS protocol published up to now, which identified 307 XL-peptides from 5 µg of HeLa lysate using O-MICROFASP and tips-based SCX66).

Thank you for your explanation; however, the original text at line 294 still states “using >20 chromatography fractions.” In addition, for the comment at line 395 (“from 5 µg of HeLa lysate using O-MICROFASP and tip-based SCX66”), please correct the reference from 66 to 67.
We thank the reviewer for pointing out this reference number, the mistake has been corrected.

Moreover, while it is indeed a remarkable result that more than 1,000 unique cross-linked peptides were identified without enrichment or in-solution fractionation from an injection of 5.5 µg total, the sample was prepared from 100 µg of HeLa cell lysate and therefore cannot be considered comparable to similar or lower starting material amounts mentioned above (Ref. 67). A starting material of 100 µg represents more favorable conditions for all sample prep steps, including cross-linking, reduction/alkylation, and digestion, compared with experiments performed using limited sample amounts.
I suggest comparing the proposed workflow with the workflow described in Ref. 63, as the starting material amount and overall experimental time are similar.
We thank the reviewer for this important remark. We agree that a direct comparison based solely on injection amount is not fully appropriate, as the use of higher starting material can facilitate several sample preparation steps, including cross-linking efficiency and digestion. We would like to clarify, however, that in most published studies, including Ref. 63, the exact amount of starting material is not explicitly reported. Typically, these workflows are performed starting from full cell culture dishes, which, for HeLa cells, generally correspond to protein amounts of ≥1 mg, i.e., substantially higher than the 100 µg used in our study. Therefore, while we agree that starting material is an important parameter, our workflow is in fact performed on comparatively limited material relative to common practice.

My minor comments were in general addressed except

- References 30 &36 are identical. Reference 30 is a preprint of reference 36 and needs to be replaced/merged by 36.
Unfortunately, there are still two identical references (References 30 and 33). Please replace Reference 30 with Reference 33 (per review published manuscript).
In addition, I suggest carefully proofreading the manuscript, as there are still a significant number of typographical errors.
We thank the reviewer for these remarks: we have corrected the references mistakes and proofread carefully the manuscript.

The authors provided detailed responses to my concerns, and overall I can see improvements in the clarity of the writing and the results. However, some of my concerns and typographical errors were not completely addressed or corrected.

Major concerns

1) What is exactly the role of Quality Control (QC) by Mass Photometry (MP) prior crosslinking? Why denatured MP was performed vs denatured intact MS analysis. Looks like native /denatured MS analysis will provide the same QC data and was actually used in some experiments.

Answer: We would like to thank the reviewer for this question. In this study we first use mass photometry in native conditions as an initial sample quality assessment. Thus, before cross-linking reaction, mass photometry is carried on intact spliceosome in its native buffer to assess its integrity. It is very useful as an alternative to native-MS, which is highly challenging for high-mass complexes (>1 MDa) with RNA bound and requires extensive buffer exchange that might induce dissociation.

To control the XL reactions, SDS-PAGE is usually the reference method because it is fast and robust visual method. However, for such samples it is also not very precise. Denaturing intact mass spectrometry is theoretically a possible approach for XL reaction control, but results in practice in highly heterogeneous and complex mass spectra for the following reasons: (i) high mass complexes (> 200 proteins, including PTMs) makes the MS data treatment complicated (overlapping signals of proteins and proteoforms), (ii) ionization issues related to high mass complexes' analysis, even more with XL reagents and (iii) heterogeneity of the cross-linking reaction (multiple cross-linker additions). To tackle these issues, we recently developed and applied an approach called denaturing MP if to confirm the XL reaction as an alternative (Gizardin-Fredon et al. 2024, Nat. Comm.). It gives a fast readout over an extended mass range, with a low sample consumption and high throughput.

This explanation is acceptable ; however, I note that Figure 3 (panels a and b) and Supplementary Figure 3 contain the same MP results for CAK sample. I suggest removing Supplementary Figure 3. For clarification, MP was performed for both the CAK sample and the spliceosome sample. Additionally, the Figure S20 legend contains a typographical error in panel d.

2) Manuscript will benefit if authors provide a direct comparison of the two methods at least for CAK samples: 4 internal CV method vs 4 external CV method using the same CVs with similar gradient length of 4 hrs and similar sample load. (same amount global length of injection). Authors did some comparison as in supplementary figure 1, but in this case internal CV gradient was equal to 1 external CV method or twice shorter and reported difference was probably due to instrument speed.

Answer: We thank the reviewer for this constructive suggestion. Following this remark, we performed additional experiments to directly compare internal versus external CV stepping under equivalent analytical LC conditions. Specifically, we analyzed a newly prepared DSSO-cross-linked HeLa lysate using both 2-CV and 4-CV methods, ensuring comparable total separation times (2 h for 2-CV internal stepping and 4 h for 4-CV external stepping) and identical sample loading.

The results confirm that external CV stepping provides a consistent performance advantage even under matched acquisition times. We observed on average 25% increase in unique cross-link identifications for the 2-CV setup and a 50% increase for the 4-CV setup when using external stepping compared to internal stepping (see new graph in **Supplementary Figure 2**).

While the magnitude of improvement is slightly reduced relative to shorter gradients, likely reflecting the greater overall sampling depth at longer gradient lengths, the benefit of external stepping remains significant and reproducible, reinforcing our conclusion that CV fractionation across independent runs provides superior identification depth.

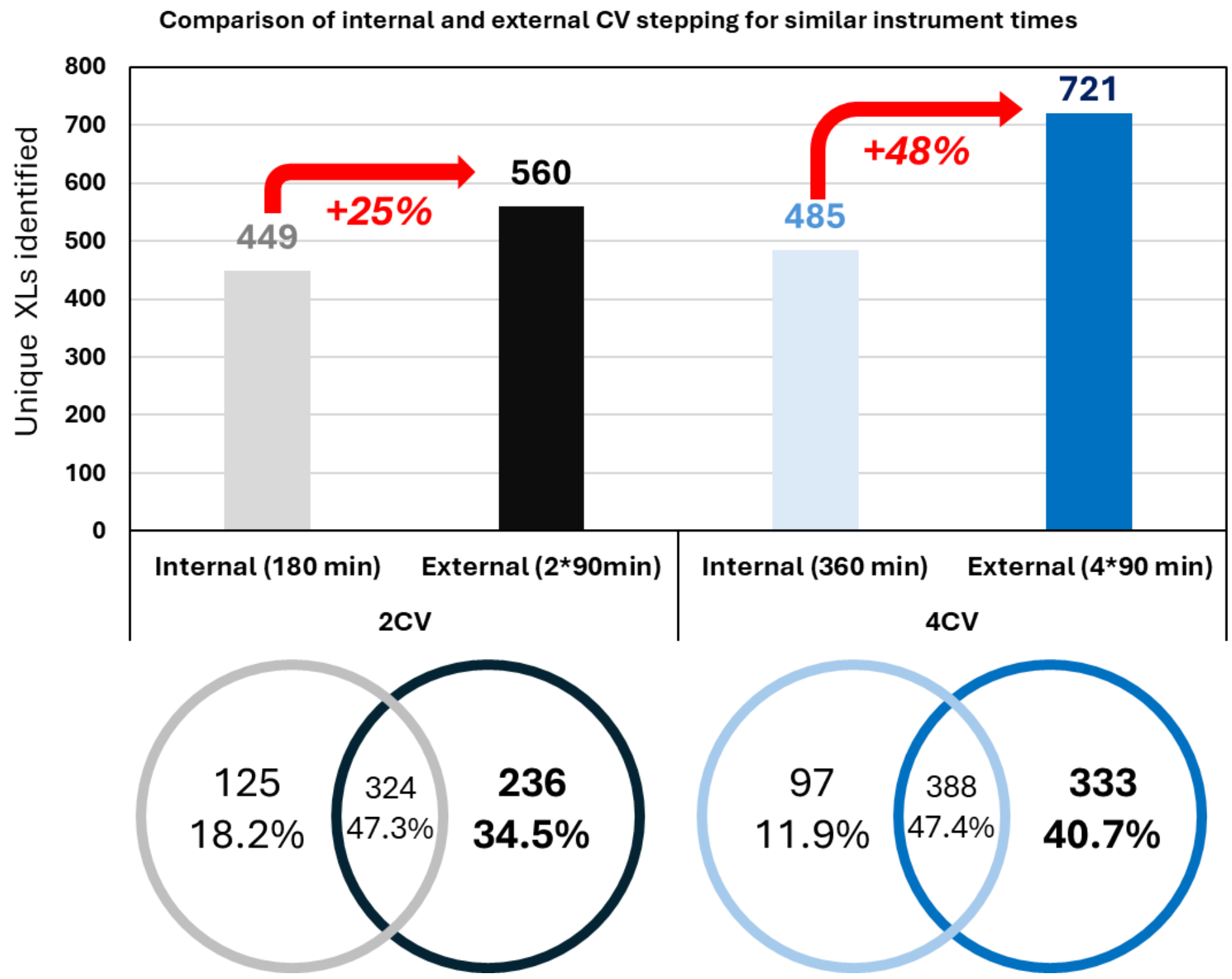

I greatly appreciate that the authors considered my suggestion and performed 2- and 4- internal stepping CV experiments using comparable instrument time. However, after carefully reviewing the Methods section, I noticed that some experimental parameters do not appear to be optimal for internal stepping experiments. It would be helpful if the authors could confirm whether a dynamic exclusion setting of 60 seconds/CV was used for both types of experiments.

For reference, the recommended dynamic exclusion setting for FAIMS internal CV experiments is the global option, where a single global inclusion list is created, as this improves the identification of unique precursors. Additionally, it would be beneficial to include the number of CSMs in Supplementary Figure 2 to allow comparison of the overall identification rate and reinforce the main conclusion of this manuscript - the benefit of external stepping remains significant and reproducible.

One additional comment regarding the experimental setup: for the Orbitrap Eclipse operated at 300 nL/min, the recommended FAIMS total carrier gas flow is 3.9–4.0 L/min, rather than 4.6 L/min as stated in the manuscript.

3) Authors highlighted low amount of sample and short instrument time as additional benefits for new developed strategy and compared number of proteome level identified XL with obtained from SEC or SCX fractionation using higher amount of stating material as on page 13: At a proteome-wide level, the 11-CV FAIMS-GPF protocol using a total of 20 µg of starting material was able to provide 1269 XLs and 213 PPIs in less than 3 days, numbers that were previously only reached after extensive in-solution fractionation (e.g. using ≥ 20 strong cation exchange fractions for a total of ~500 µg of starting material and requiring weeks of sample prep and instrument time)27,28,62,63,81. Maybe this is accurate statement for the earlier results as in references 27& 28 but number of identified crosslinkers for example in reference 63 is significantly higher – it reported 4260 unique crosslinkers from 1357 proteins in 24 hrs of instrument time using 1 SEC fraction and 12 High pH fractions –

please revise lines 371-373 accordingly to reflect current status of in-solution fractionation with/without FAIMS for proteome wide crosslinking analysis

Answer: We thank reviewer for this comment, we modified the text accordingly: "e.g. using ≥ 10 to 20 chromatography fractions for a total of > 500 to 100 µg of starting material and requiring days to weeks of sample prep and instrument time". Additionally, the most sensitive protocol published, starting from 5 ug of HeLa lysate, identified 307 cross-linked peptides with DSSO using MICROFASP and tips-based SCX fractionation. Our FAIMS-GPF clearly outperforms (4-fold improvement) these results with similar or lower starting material amounts, while removing in-solution fractionation. This reference and sentences regarding these results were added in main text and in discussion (l.296: "Notably, our approach is the first to remove in-solution fractionation in proteome-wide protocols while outperforming by four folds the most sensitive workflow published up to now*"; l.395:* Finally, our approach also outperforms by 4-fold the most sensitive proteome-wide XL-MS protocol published up to now, which identified 307 XL-peptides from 5 µg of HeLa lysate using O-MICROFASP and tips-based SCX[66])

Thank you for your explanation; however, the original text at line 294 still states "using >20 chromatography fractions." In addition, for the comment at line 395 ("from 5 µg of HeLa lysate using O-MICROFASP and tip-based SCX66"), please correct the reference from 66 to 67.

Moreover, while it is indeed a remarkable result that more than 1,000 unique cross-linked peptides were identified without enrichment or in-solution fractionation from an injection of 5.5 µg total, the sample was prepared from 100 µg of HeLa cell lysate and therefore cannot be considered comparable to similar or lower starting material amounts mentioned above (Ref. 67). A starting material of 100 µg represents more favorable conditions for all sample prep steps, including cross-linking, reduction/alkylation, and digestion, compared with experiments performed using limited sample amounts.

I suggest comparing the proposed workflow with the workflow described in Ref. 63, as the starting material amount and overall experimental time are similar.

My minor comments were in general addressed except

- References 30 &36 are identical. Reference 30 is a preprint of reference 36 and needs to be replaced/merged by 36.

We thank reviewer for this remark, this has been modified in the text.

Unfortunately, there are still two identical references (References 30 and 33). Please replace Reference 30 with Reference 33(per review published manuscript).

In addition, I suggest carefully proofreading the manuscript, as there are still a significant number of typographical errors.
